# Lactones from the Sponge-Derived Fungus *Talaromyces rugulosus*

**DOI:** 10.3390/md15110359

**Published:** 2017-11-14

**Authors:** Lisa Küppers, Weaam Ebrahim, Mona El-Neketi, Ferhat C. Özkaya, Attila Mándi, Tibor Kurtán, Raha S. Orfali, Werner E. G. Müller, Rudolf Hartmann, Wenhan Lin, Weiguo Song, Zhen Liu, Peter Proksch

**Affiliations:** 1 Institute of Pharmaceutical Biology and Biotechnology, Heinrich-Heine-Universität Düsseldorf, 40225 Düsseldorf, Germany; li-kue@gmx.de (L.K.); weaam.ebrahim@uni-duesseldorf.de (W.E.); fcanozkaya@gmail.com (F.C.Ö.); 2 Department of Pharmacognosy, Faculty of Pharmacy, Mansoura University, Mansoura 35516, Egypt; monaneketi@yahoo.com; 3 Department of Organic Chemistry, University of Debrecen, 4032 Debrecen, Hungary; mandi.attila@science.unideb.hu (A.M.); kurtan.tibor@science.unideb.hu (T.K.); 4 Department of Pharmacognosy, Faculty of Pharmacy, King Saud University, Riyadh 11451, Saudi Arabia; rom2leen@gmail.com; 5 Institute of Physiological Chemistry, Universitätsmedizin der Johannes Gutenberg-Universität Mainz, 55128 Mainz, Germany; wmueller@uni-mainz.de; 6 Institute of Complex Systems: Strukturbiochemie, ForschungszentrumJuelich, 52428 Juelich, Germany; r.hartmann@fz-juelich.de; 7 State Key Laboratory of Natural and Biomimetic Drugs, Peking University, Beijing 100191, China; whlin@bjmu.edu.cn; 8 FukangPharma, North-East of Dongwaihuan Road, Dongcheng Industrial Area, Shouguang City 262700, China; songwg@139.com

**Keywords:** *Talaromyces rugulosus*, lactones, ECD calculation, cytotoxicity

## Abstract

The marine-derived fungus *Talaromyces rugulosus* isolated from the Mediterranean sponge *Axinella cannabina* and cultured on solid rice medium yielded seventeen lactone derivatives including five butenolides (**1**–**5**), seven (3*S*)-resorcylide derivatives (**6**–**12**), two butenolide-resorcylide dimers (**13** and **14**), and three dihydroisocoumarins (**15**–**17**). Among them, fourteen compounds (**1**–**3**, **6**–**16**) are new natural products. The structures of the isolated compounds were elucidated by 1D and 2D NMR (Nuclear Magnetic Resonance) spectroscopy as well as by ESI-HRMS (ElectroSpray Ionization-High Resolution Mass Spectrometry). TDDFT-ECD (Time-Dependent Density Functional Theory-Electronic Circular Dichroism) calculations were performed to determine the absolute configurations of chiral compounds. The butenolide-resorcylide dimers talarodilactones A and B (**13** and **14**) exhibited potent cytotoxicity against the L5178Y murine lymphoma cell line with IC_50_ values of 3.9 and 1.3 µM, respectively.

## 1. Introduction

Marine-derived fungi are prolific sources for bioactive secondary metabolites, such as the famous antibiotic cephalosporin C obtained from the fungus *Acremonium chrysogenum* [1], or halimide derived from an algiculous fungus, which gave rise to the semisynthetic metabolite plinabulin that is now in Phase II clinical studies for its anticancer potential against either solid tumours or lymphomas [2,3].

During our ongoing search for new bioactive secondary metabolites from fungal origin [4,5,6,7], we investigated *Talaromyces rugulosus*, which was isolated from the Mediterranean sponge *Axinella cannabina*. The fungal genus *Talaromyces* is known to produce diverse bioactive secondary metabolites including tetraene lactones [8], polyketides [9,10], alkaloids and peptides with some of them exhibiting antibiotic, cytotoxic and anti-HBV (Hepatitis B Virus) activities [11]. Only a few research papers are devoted to the chemical constituents of *Talaromyces rugulosus*. Yilmaz et al. reported the isolation of skyrin, endocrocin, emodin, prugosins A-C and a bis-anthraquinoid pigment, rugulosin A, which showed antibacterial activity against *Staphylococcus aureus* [12]. This encouraged us to investigate the natural products of the sponge-derived strain of *T. rugulosus*.

**Figure 1:**
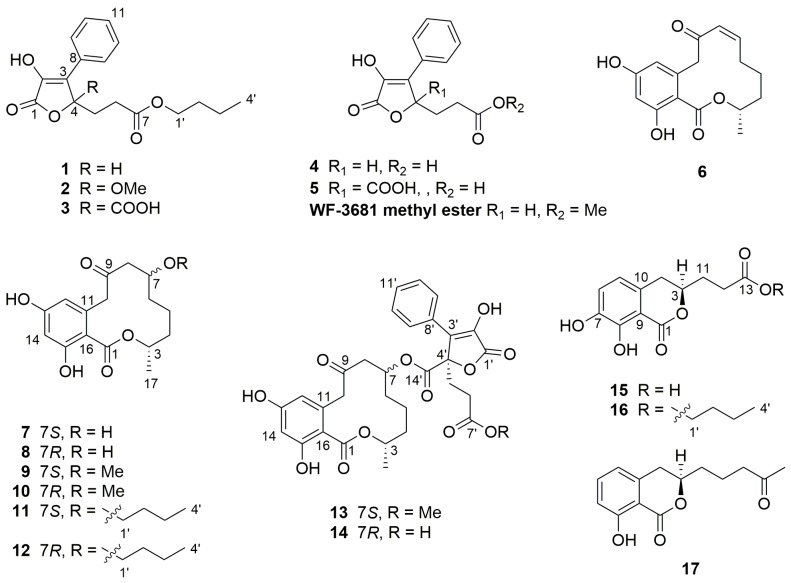
Structures of the compounds isolated from *Talaromyces rugulosus*.

The EtOAc (ethyl acetate) extract of the fungus when fermented on solid rice medium yielded three new butenolides (**1**–**3**), seven new resorcylide derivatives (**6**–**12**), two new butenolide-resorcylide dimers (**13** and **14**) and two new dihydroisocoumarins (**15** and **16**) as well as three known analogues (**4**, **5** and **17**) (Figure 1). The structure elucidation including the absolute configuration assignment and biological activities of the isolated compounds are discussed in this report.

## 2. Results

### 2.1. Structure Elucidation

Compound **1** was isolated as a brown amorphous solid. It has the molecular formula C_17_H_20_O_5_ as determined by ESI-HRMS (ElectroSpray Ionization-High Resolution Mass Spectrometry) corresponding to eight degrees of unsaturation. Its UV absorbances at *λ*_max_ 207, 218 and 288 nm were similar to those of the co-isolated known butenolide derivative lactone acid (**4**) [13]. The NMR (Nuclear Magnetic Resonance) data of compound **1** (Table 1) were likewise comparable to those of **4** and included typical signals for a *mono*-substituted benzene ring at *δ*_H_ 7.75 (2H, d, *J* = 7.5 Hz, H-9 and H-13), 7.44 (2H, t, *J* = 7.5 Hz, H-10 and H-12) and 7.35 (1H, t, *J* = 7.5 Hz, H-11), an oxygenated methine proton at *δ*_H_ 5.52 (H-4), as well as two methylene groups at *δ*_H_ 2.45, 1.75 (H_ab_-5) and 2.44 (H_2_-6). The COSY (Correlation SpectroscopY) correlations between H-4/H_2_-5 and H_2_-5/H_2_-6 together with the HMBC (Heteronuclear Multiple Bond Correlation) correlations from H-4 to C-1 (*δ*_C_ 171.0), C-2 (*δ*_C_ 139.5), C-3 (*δ*_C_ 130.6) and C-8 (*δ*_C_ 131.9) and from H_ab_-5 and H_2_-6 to C-7 (*δ*_C_ 174.6) indicated that compound **1** shared the same core structure as **4**. In addition, signals of a methyl at *δ*_H_ 0.94 (Me-4′) and three methylene groups at *δ*_H_ 1.38 (H_2_-3′), 1.60 (H_2_-2′), 4.08 (H-1′a) and 4.04 (H-1′b) were observed. The COSY correlations between Me-4′/H_2_-3′/H_2_-2′/H_ab_-1′ and the HMBC correlation from H_ab_-1′ to C-7 confirmed a *n*-butyl side chain to be attached to C-7 through an ester bond. Thus, the planar structure of **1** was elucidated as an unsaturated γ-lactone with propanoic acid *n*-butyl ester side chain. The absolute configuration of **1** was elucidated by comparing its experimental ECD spectrum with that of WF-3681 methyl ester [14], which can be considered a close analogue of **1**. Based on the good overall agreement, **1** has (*S*) absolute configuration.

The UV (UltraViolet) spectrum of compound **2** was similar to that of **1**. The ESI-HRMS data revealed the molecular formula C_18_H_22_O_6_, suggesting the presence of an additional methoxy group in **2** compared to **1**, which was observed at *δ*_H_ 3.18 and *δ*_C_ 50.7, respectively. The disappearance of the oxygenated methine proton along with the HMBC correlation from the protons of the methoxy group to C-4 (*δ*_C_ 109.8) indicated this additional methoxy group to be located at the C-4 position. The remaining structure of **2** was elucidated to be identical to that of **1** as indicated by detailed analysis of the 2D NMR spectra of **2**. Thus, compound **2** was identified as 4-methoxylactone acid *n*-butyl ester. The ECD spectrum of **2** was rather weak and noisy, but similar to that of chaetobutenolide C [14], which can be used for ECD correlation and indicated a non-racemic mixture of (*R*) and (*S*) isomers with a slight excess of the (*R*) enantiomer.

The molecular formula of **3** was deduced as C_18_H_20_O_7_ from ESI-HRMS data. The UV and NMR data (Table 1) of **3** resembled those of the co-isolated lactone diacid (**5**) [13], except for the observation of an additional *n*-butyl chain as described for **1**. In the HMBC spectrum of **3**, H_2_-5 (*δ*_H_ 2.68 and 2.48), H_2_-6 (*δ*_H_ 2.25 and 2.15) and H_2_-1′ (*δ*_H_ 4.00 and 3.94) exhibited correlations to C-7 (*δ*_C_ 174.2) while only H_2_-5 showed correlation to C-14 (*δ*_C_ 171.9), indicating the *O*-*n*-butyl group to be attached to C-7. Thus, compound **3** was elucidated as lactone diacid 7-*O*-*n*-butyl ester. Compound **3** decomposed before ECD measurements. Its absolute configuration can be tentatively assigned upon biosynthetic considerations as (*R*) similarly to **5**.

**Figure 2:**
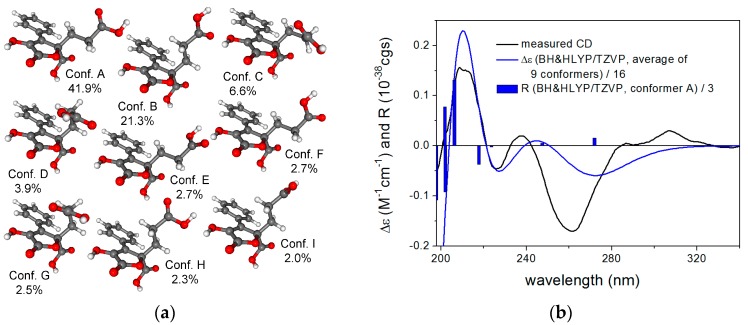
(**a**) structures and populations of the low-energy B3LYP/6-31G(d) in vacuo conformers (≥2%) of (*R*)-**5**; (**b**) experimental ECD spectrum of **5** in MeCN compared with the Boltzmann-weighted BH&HLYP/TZVP ECD spectrum of (*R*)-**5** computed for the B3LYP/6-31G(d) in vacuo conformers. Bars represent the rotational strength values of the lowest-energy conformer.

To elucidate the absolute configuration of the known natural product **5**, the solution TDDFT-ECD (Time-Dependent Density Functional Theory-Electronic Circular Dichroism) protocol was applied on the arbitrarily chosen (*R*) enantiomer [15,16]. Merck Molecular Force Field (MMFF) conformational search with an implicit solvent model for CHCl_3_ resulted in 149 conformers in a 21 kJ/mol energy window. DFT reoptimization of these yielded 9, 12 and 12 low-energy (≥2%) conformers at the B3LYP/6-31G(d) in vacuo, the B97D/TZVP PCM (Polarizable Continuum Model)/MeCN and the CAM-B3LYP/TZVP PCM/MeCN levels, respectively. Boltzmann-averaged ECD spectra of (*R*)-**5** resembled the experimental ECD spectrum allowing elucidation of the absolute configuration as (*R*) (Figure 2).

**Figure 3:**
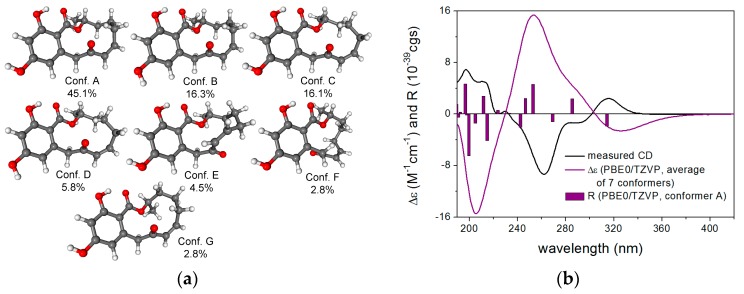
(**a**) structures and populations of the low-energy B3LYP/6-31G(d) in vacuo conformers (≥2%) of (*R*)-**6**; (**b**) experimental ECD spectrum of **6** in MeCN compared with the Boltzmann-weighted PBE0/TZVP ECD spectrum of (*R*)-**6** computed for the B3LYP/6-31G(d) in vacuo conformers. Bars represent the rotational strength values of the lowest-energy conformer.

The NMR data of compounds **6**–**10** were found to be identical to those of (3*R*)-*cis*-resorcylide, (3*R*,7*R*)-7-hydroxyresorcylide, (3*R*,7*S*)-7-hydroxyresorcylide, (3*R*,7*R*)-7-methoxyresorcylide and (3*R*,7*S*)-7-methoxyresorcylide, respectively (Table 2 and Table 3). These resorcylides were first reported by Barrow et al. and the absolute configuration at C-3 was suggested to be *S* in consideration of their biosynthesis [17]. Later, it was revised to (3*R*) by chemical derivatization, ECD calculation and X-ray diffraction analysis [18,19,20]. However, the resorcylides isolated in this study exhibited mirror-imaged CD (Circular dichroism) curves compared to those of the corresponding compounds found in the literature, indicating that they should be enantiomers of the previously reported resorcylides derivatives and possess 3*S* configuration. The above-mentioned TDDFT-ECD protocol was carried out on the arbitrarily chosen (*R*) enantiomer of **6** yielding 33 MMFF conformers, and 7 and 12 low-energy conformers at the B3LYP/6-31G(d) and the B97D/TZVP levels. Boltzmann averaged ECD spectra computed for both sets of conformers gave mirror-image agreement with the experimental spectrum, allowing elucidation of the absolute configuration as (*S*) in line with the literature (Figure 3).

Similar ECD pattern was observed in the experimental ECD spectrum of **9**, and good agreement of the experimental and TDDFT-ECD spectra allowed determining the absolute configuration as (3*S*,7*S*)-**9** (Figure S92). The calculations also confirmed that the stereochemistry of the C-3 lactone carbon has dominant contribution to the ECD spectra. Thus, compounds **6**–**10** are determined as (3*S*)-*cis*-resorcylide, (3*S*,7*S*)-7-hydroxyresorcylide, (3*S*,7*R*)-7-hydroxyresorcylide, (3*S*,7*S*)-7-methoxy-resorcylide and (3*S*,7*R*)-7-methoxyresorcylide, respectively.

Based on ESI-HRMS data, the molecular formula of compound **11** was determined to be C_20_H_28_O_6_, containing an additional C_4_H_8_ unit when compared to **7**. Comparison of the ^1^H and ^13^C NMR data of **7** and **11** (Table 4) revealed the existence of an additional *n*-butyl group in **11**, which is further confirmed by the COSY correlations between Me-4′ (*δ*_H_ 0.95)/H_2_-3′ (*δ*_H_ 1.42), H_2_-3′/H_2_-2′ (*δ*_H_ 1.56), H_2_-2′/H_a_-1′ (*δ*_H_ 3.63) and H_2_-2′/H_b_-1′ (*δ*_H_ 3.51). Examination of the 2D NMR spectra of **11** indicated it to share the same resorcylide ring structure as found in compounds **7**–**10**. Key HMBC correlations from H_ab_-1′ to C-7 (*δ*_C_ 75.4) and from H-7 (*δ*_H_ 4.04) to C-1′ (*δ*_C_ 69.7) confirmed the linkage between C-7 and the additional *n*-butyl group via an ether bond in **11**. Thus, the planar structure of **11** was elucidated as 7-*O*-*n*-butylresorcylide. Compound **12** shared the same gross structure as **11** as confirmed by 2D NMR and ESI-HRMS data. The structural difference between **11** and **12** is confined to the configuration at C-7 as already found for **7** and **8**, and for **9** and **10**. Comparison of the NMR data of **11** and **12**, especially of the coupling constants between H-7 and H_ab_-8 and of the chemical shifts of C-3, H-3 and C-17 (Table 5), indicated that **11** shared the same relative configuration as **7** and **9,** whereas **12** shared the same relative configuration as **8** and **10**. Moreover, the CD data of **11** were comparable to those of **7** and **9,** whereas the ECD data of **12** resembled those of **8** and **10**. The above findings revealed compounds **11** and **12** to be (3*S*,7*S*)-7-*O*-*n*-butylresorcylide and (3*S*,7*R*)-7-*O*-*n*-butylresorcylide, respectively.

Compound **13** was isolated as a brown amorphous solid. Its molecular formula was established as C_31_H_32_O_12_ from ESI-HRMS data. Interestingly, the ^1^H and ^13^C NMR spectra of **13** (Table 6) showed two sets of signals corresponding to the macrolide (3*R*,7*R*)-7-hydroxyresorcylide (**7**) and the butenolide lactone diacid (**5**), which were co-isolated in the present study. For example, the two *meta*-coupling aromatic protons at *δ*_H_ 6.26 (d, *J* = 2.5 Hz, H-14) and 6.14 (d, *J* = 2.5 Hz, H-12), the two oxygenated methine protons at *δ*_H_ 5.54 (m, H-7) and 4.93 (m, H-3), the isolated methylene protons at *δ*_H_ 4.67 (d, *J* = 18.8 Hz, H_a_-10) and 3.78 (d, *J* = 18.8 Hz, H_b_-10) and the doublet methyl at *δ*_H_ 1.28 (d, *J* = 6.2 Hz, Me-17) represented characteristic signals for 7-hydroxyresorcylide. On the other side, signals of the *mono*-substituted benzene ring at *δ*_H_ 7.73 (d, *J* = 7.7 Hz, H-9′ and H-13′), 7.44 (2H, t, *J* = 7.7 Hz, H-10′ and H-12′) and 7.37 (1H, t, *J* = 7.7 Hz, H-11′) along with those of two methylene groups at *δ*_H_ 2.77, 2.59 (H_ab_-5′) and 2.29, 2.22 (H_ab_-6′) indicated the butenolide lactone diacid (**5**).

Detailed analysis of the 2D NMR spectra of **13** established the two subunits of resorcylide and butenolide as shown. The HMBC correlations from H-7 and H_ab_-5′ to C-14′ (*δ*_C_ 169.9) indicated the connection of these two subunits through the C(7)-O-C(14′) ester bond. In addition, the attachment of a methoxy group (*δ*_H_ 3.58 and *δ*_C_ 52.3) at C-7′ was deduced by the HMBC correlation from the protons of the methoxy group to C-7′ (*δ*_C_ 174.4). Thus, the structure of compound **13** was elucidated as shown, representing a butenolide-resorcylide dimer, for which the name talarodilactone A is proposed. Comparison of its NMR data with those of other resorcylide derivatives (Table 5) indicated that compound **13** shared the same relative configuration as **7**, **9** and **11** regarding the macrolactone part. The ECD spectrum of **13** was found to be similar to those of resorcylide monomers, which suggested that the ECD spectrum of **13** is mainly governed by the C-3 chiral center of the resorcylide moiety and thus the absolute configuration of this chiral center could be unambiguously elucidated as (3*S*). On the basis of the (3S*,7S*) relative configuration determined by NMR experiments, the absolute configuration of the RAL subunit of **13** was determined as (3*S*,7*S*). Considering the shape of the ECD spectrum of **13,** it more resembles those of **7** and **9** than those of **8** and **10** supporting the assignment of C-7 as (*S*). The absolute configuration of the butenolide part can be tentatively assiged as (*R*) on the basis of biosynthetic considerations.

The molecular formula of compound **14** was determined to be C_30_H_30_O_12_, suggesting the loss of one methoxy group compared to **13**, which was confirmed by the disappearance of the respective signal in the NMR spectra of **14**. Furthermore, comparison of the NMR data of **14** with those of the other resorcylide derivatives isolated in this study (Table 5) revealed that **14** shared the same relative configuration as **8**, **10** and **12** at C-3 and C-7. Similarly to **13**, the (3*S*) absolute configuration of **14** can be unambiguously deduced from the ECD spectrum, while that of C-7 can be determined as (*R*) by utilizing the experimental NMR data. The C-4′ chiral center can be only tentatively assigned as (*R*) on the basis of biosynthetic considerations.

Compound **15** was isolated as a brown amorphous solid, exhibiting UV absorbance maxima at 220, 258 and 334 nm. Its molecular formula was found to be C_12_H_12_O_6_ as confirmed by ESI-HRMS data, indicating seven degrees of unsaturation. The ^1^H NMR spectrum of **15** (Table 7) showed two *ortho*-coupling protons at *δ*_H_ 7.01 (d, *J* = 8.0 Hz, H-6) and 6.63 (d, *J* = 8.0 Hz, H-5), suggesting the presence of a 1,2,3,4-tetrasubstituted benzene ring. A spin system containing an oxygenated methine at *δ*_H_ 4.63 (H-3) and three aliphatic methylenes at *δ*_H_ 2.94 and 2.83 (H_ab_-4), 1.96 (H_2_-11) and 2.42 (H_2_-12) was identified by their COSY correlations. In addition, three hydroxy groups at *δ*_H_ 12.21 (s, OH-13), 10.82 (s, OH-8) and 9.33 (s, OH-7) were observed. The HMBC correlations from H-5 to C-7 (*δ*_C_ 144.4) and C-9 (*δ*_C_ 108.4), from H-6 to C-8 (*δ*_C_ 149.9) and C-10 (*δ*_C_ 129.1), from OH-7 to C-6 (*δ*_C_ 121.6), C-7 and C-8, and from OH-8 to C-7, C-8 and C-9 established a benzene ring with two hydroxy groups at C-7 and C-8. The HMBC correlations from H-5 to C-4 (*δ*_C_ 31.2), from H_ab_-4 to C-5 (*δ*_C_ 117.5), C-9 and C-10 as well as from H_2_-11 and H_2_-12 to C-13 (*δ*_C_ 173.8) indicated a 5-carboxypentyl chain to be attached at the C-10 position. Furthermore, the presence of an additional six-membered lactone ring fused with the benzene ring was confirmed by the HMBC correlations from H-5 and H-3 to C-1 (*δ*_C_ 169.6) combined with its molecular formula. The above findings concluded the structure of **15** as a new dihydroisocoumarin derivative as shown, for which the trivial name talumarin A is proposed.

Compound **16** shares almost identical UV absorptions with **15**, suggesting the dihydroisocoumarin nature of the latter. The ESI-HRMS data established the molecular formula C_16_H_20_O_6_ for **16**. The NMR data of **16** were likewise similar to those of **15** (Table 7). However, signals of an additional *n*-butyl moiety were observed in the NMR spectra of **16**, which was further confirmed by the COSY correlations between Me-4′ (*δ*_H_ 0.95)/H_2_-3′ (*δ*_H_ 1.40), H_2_-3′/H_2_-2′ (*δ*_H_ 1.63), and H_2_-2′/H_2_-1′ (*δ*_H_ 4.11). In the HMBC spectrum, H_2_-1′ showed correlation to C-12 (*δ*_C_ 174.4), indicating the attachment of the *n*-butyl moiety to C-12 through an ester bond. The remaining structure of **16** was found to be identical to that of **15** by detailed analysis of the 2D NMR data of **16**.

**Figure 4:**
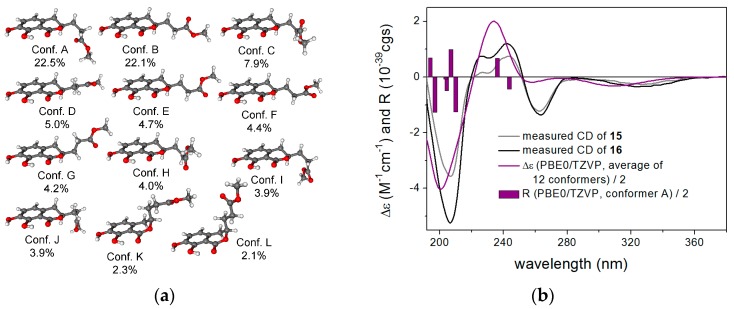
(**a**) structures and populations of the low-energy CAM-B3LYP/TZVP PCM/MeCN conformers (≥2%) of the truncated model compound of (*R*)-**16**; (**b**) experimental ECD spectra of **15** and **16** in MeCN compared with the Boltzmann-weighted PBE0/TZVP PCM/MeCN ECD spectrum of the truncated model compound of (*R*)-**16** computed for the CAM-B3LYP/TZVP PCM/MeCN conformers. Bars represent the rotational strength values of the lowest-energy conformer.

To elucidate the absolute configuration of **15** and **16**, TDDFT-ECD calculations were performed on a truncated model compound of **16** (R = Me). MMFF conformational search of the (*R*) enantiomer resulted in 42 conformers in a 21 kJ/mol energy window—DFT reoptimization of which yielded 8, 18 and 12 low-energy conformers at the B3LYP/6-31G(d) in vacuo, the B97D/TZVP PCM/MeCN and the CAM-B3LYP/TZVP PCM/MeCN levels. ECD calculations performed for each set of conformers gave moderate to good overall agreement with the experimental spectra of **15** and **16,** allowing elucidation of the absolute configuration of **15** and **16** as (*R*) (Figure 4). The result of the ECD calculation corroborated with the previous ECD studies of the dihydroisocoumarin chromophore well [21,22,23], which located the n-π* ECD transition in the range of 250–270 nm and correlated the *P*/*M* helicity of the condensed heteroring with *positive/negative* n-π* Cotton effect, respectively. In the case of **15** and **16**, the heteroring adopts *M* helicity with equatorial C-3 substituent, which gives rise to a negative n-π* Cotton effect at 263 and 262 nm, respectively.

The remaining known compounds were identified as lactone acid (**4**) [13], lactone diacid (**5**) [13], and aspergillumarin A (**17**) by comparison of their spectroscopic data with those in the literature [24,25].

### 2.2. Biological Activities

All isolated compounds (**1**–**17**) were evaluated for their cytotoxic activity against the L5178Y mouse lymphoma cell line. Only the two butenolide-resorcylide dimmers talarodilactones A and B (**13** and **14**) exhibited potent cytotoxicity with IC_50_ (half maximal Inhibitory Concentration) values of 3.9 and 1.3 µM, respectively. Interestingly, the monomeric building blocks of **13** and **14,** which were also isolated in this study, were inactive in comparison.

Moreover, all compounds were tested for their antimicrobial activities against *Mycobacterium tuberculosis* (H37Rv), *Staphylococcus aureus* (ATCC 25923) and *Acinetobacter baumannii* (ATCC BAA-1605) using a broth micro dilution assay. However, none of them was active at a dose of 25 µg/mL.

## 3. Discussion

In conclusion, analysis of the sponge-derived fungus *T. rugulosus* afforded seventeen lactone derivatives divided into four groups: five butenolides (**1**–**5**), seven resorcylide derivatives (**6**–**12**), two butenolide-resorcylide dimers (**13** and **14**) and three dihydroisocoumarins (**15**–**17**).Compounds **1**–**3**, **11**, **12** and **16** exhibited a butyl side chain in their structures. Many butylated natural products have been isolated from fungi [26,27,28] and plants [29,30]. In our study, all butylated derivatives were clearly detected in the original crude fungal extract by LC-MS. Moreover, the extraction and isolation processes did not involve *n*-BuOH. After incubation of the corresponding non-butylated compounds with *n*-BuOH for one week, no butylation of these compounds was detected by LC-MS. These results confirm that the butylated compounds isolated in the present study are natural compounds.

Numerous (3*R*)-resorcylide derivatives have been reported [17,18,19,20]. Some of them that were initially presumed to possess 3*S* configuration were later revised to have 3*R* configuration instead. However, all resorcylide derivatives (**6**–**14**) isolated in the present study were unambiguously assigned as (3*S*)-series by CD analysis. To the best of our knowledge, this is the first report of (3*S*)-resorcylide derivatives in nature. In addition, talarodilactones A and B (**13** and **14**) represent a new class of butenolide-resorcylide dimers that showed significant cytotoxicity against the L5178Y murine lymphoma cell line with IC_50_ values of 3.9 and 1.3 µM, respectively.

## 4. Materials and Methods

### 4.1. General Experimental Procedures

Optical rotations were measured using a PerkinElmer-241 MC polarimeter (Waltham, MA, USA). Bruker AVANCE DMX 300 or 600 NMR spectrometers (Karlsruhe, Germany) were employed to record ^1^H, ^13^C and 2D NMR spectra. A Thermo Finnigan LCQ Deca LC-MS system (San Jose, CA, USA) was used to analysis the crude extract. ESI-HRMS data were obtained by a FT-HRMS-Orbitrap (Thermo Finnigan, San Jose, CA, USA) mass spectrometer. A Dionex P580 system (Germering, Germany) coupled to a photodiode array detector (UVD340S) was used to perform HPLC analysis. A Europhere 10 C_18_ (125 × 4 mm, L × ID, Knauer, Germany) was used for analytical separation. HPLC separation was carried out with a Lachrom-Merck Hitachi semi-preparative HPLC system (Darmstadt, Germany) (Pump L7100; UV detector L7400; column: Europhere 100 C_18_, 300 × 8 mm, Knauer, Germany). Merck MN silica gel 60 M (0.04–0.063 mm, Dueren, Germany) or Sephadex LH-20 (Darmstadt, Germany) were applied for column chromatography as stationary phases. Pre-coated silica gel 60 F_254_ plates (Merck) were used for TLC (Thin layer chromatography) analysis under detection at 254 and 366 nm and/or using anisaldehyde as spray-reagent. Spectral grade solvents were used for spectroscopic measurements while distilled solvents were used for column chromatographic separations. ECD spectra were recorded on a J-810 spectropolarimeter.

### 4.2. Fungal Material

The fungal strain was isolated from the healthy inner tissues of the sponge *A. cannabina* collected and identified by Semih Engin at Sığaçık-İzmir, Turkey. It was identified as *Talaromyces rugulosus* (GenBank accession No. KT071708) according to DNA amplification and sequencing of the fungal ITS region as described before [31].

### 4.3. Fermentation, Extraction and Isolation

The fungal strain was cultivated on solid rice medium in twelve 1L Erlenmeyer flasks (autoclaving 100 g of rice and 110 mL of 3.5% sea salt solution in each Erlenmeyer flask) at room temperature under static conditions. After 30 d, each flask was extracted overnight with ethyl acetate (3 × 400 mL), followed by filtration and evaporation. The obtained crude extract (5 g) was then partitioned between *n*-hexane and 90% aqueous MeOH. The MeOH extract was then subjected to vacuum liquid chromatography (VLC) on silica gel 60 using a gradient solvent elution system of *n*-hexane/EtOAc and CH_2_Cl_2_/MeOH to obtain 16 fractions (Fr.1 to Fr.16).

Fr.4 (509 mg) was fractionated on a Sephadex LH-20 column using MeOH as eluent to give ten subfractions (Fr.4-1 to Fr.4-10), among which subfraction Fr.4-6 was found to be the pure compound **6** (14.0 mg). Subfraction Fr.4-5 (119 mg) was further purified by semi-preparative HPLC with 60% MeOH/H_2_O as eluting system to afford **1** (9.9 mg), **11** (2.1mg), **12** (2.5 mg) and **16** (2.0 mg).

Fr.5 (2.4 g) was subjected to a Sephadex LH-20 column with MeOH as mobile phase to yield nine subfractions (Fr.5-1 to Fr.5-9). Compounds **14** (3.9 mg) and **15** (3.1 mg) were obtained from subfraction Fr.5-6 (50.5 mg) by semi-preparative HPLC with a gradient of MeOH/H_2_O as eluent. Subfraction Fr.5-4(570 mg) was chromatographed over a Sephadex LH-20 column using acetone to afford eight subfractions (Fr.5-4-1 to Fr.5-4-8). After purification by semi-preparative RP (Reversed Phase)-HPLC using a gradient elution of MeOH/H_2_O, subfractions Fr.5-4-5 (18.9 mg) and Fr.5-4-6 (13.1 mg) yielded compounds **8** (1.2 mg) and **4** (6.2 mg) ,respectively. Subfraction Fr.5-3 (788 mg) was fractionated over a Sephadex LH-20column using MeOH as eluent to give five subfractions (Fr.5-3-1 to Fr.5-3-5). SubfractionFr.5-3-2 (47.2 mg) was purified by semi-preparative RP-HPLC using a gradient elution of MeOH/H_2_O to yield **17** (9.3 mg). Following the same procedure as described for Fr.5-3-2, compounds **2** (2.2 mg) **3** (16.7 mg) and **5** (2.4 mg) were obtained from Fr.5-3-3 (71.5 mg) while compounds **7** (1.8 mg), **9** (1.5 mg), **10** (1.4 mg) and **13** (1.6 mg) were obtained from Fr.5-3-4 (53.7 mg).

*Lactone acid n-butyl ester* (**1**): brown amorphous solid; ${\left[\alpha \right]}_{D}^{23}$ +2 (*c* 0.54, EtOH); UV *λ*_max_ 207, 217 and 288 nm; ECD {MeCN, λ [nm] (Δε), c = 3.29 × 10^−4^ M} 281 (+0.30), 231sh (−0.07); ^1^H and ^13^C NMR, see Table 1; ESI-HRMS *m*/*z* 327.1206 [M + Na]+ (calcd. for C_17_H_20_O_5_Na, 327.1203).

*4-Methoxylactone acid n-butyl ester* (**2**): brown amorphous solid; ${\left[\alpha \right]}_{D}^{23}$ −4 (*c* 0.33, EtOH); UV *λ*_max_ 203, 220 and 292 nm; ECD {MeCN, λ [nm] (Δε), c = 2.47 × 10^−4^ M} 263 (−0.32), 222 (−0.76), 210 (+0.16); ^1^H and ^13^C NMR, see Table 1; ESI-HRMS *m*/*z* 357.1308 [M + Na]+ (calcd. for C_18_H_22_O_6_Na, 357.1309).

*Lactone diacid 7-O-n-butyl ester* (**3**): brown amorphous solid; ${\left[\alpha \right]}_{D}^{23}$ −4 (*c* 0.53, EtOH); UV *λ*_max_ 204, 220 and 292 nm; ^1^H and ^13^C NMR, see Table 1; ESI-HRMS *m*/*z* 371.1101 [M + Na]+ (calcd. for C_18_H_20_O_7_Na, 371.1101).

*Lactone diacid* (**5**): brown amorphous solid; ${\left[\alpha \right]}_{D}^{23}$ −3 (*c* 0.53, EtOH); UV *λ*_max_ 219 and 292 nm; ECD {MeCN, λ [nm] (Δε), c = 3.51 × 10^−4^ M} 307 (+0.03), 262 (−0.17), 238 (+0.02), 226 (−0.05), 209 (+0.16), 198 (−0.05); LC-MS *m/z* 315.0 [M + Na]^+^, 291.3 [M − H]^−^.

*(3S)-cis-Resorcylide* (**6**): brown amorphous solid; ${\left[\alpha \right]}_{D}^{23}$ +2 (*c* 0.10, MeOH); UV *λ*_max_ 216, 266 and 303 nm; ECD {MeCN, λ [nm] (Δε), c = 3.88 × 10^−4^ M} 316 (+2.41), 288sh (−1.37), 262 (−9.35), 230sh (+0.42), 211sh (+5.08), 198 (+6.90); ^1^H and ^13^C NMR, see Table 2; ESI-HRMS *m*/*z* 291.1233 [M + H]^+^ (calcd. for C_16_H_19_O_5_, 291.1227).

*(3S,7S)-7-Hydroxyresorcylide* (**7**): brown amorphous solid; ${\left[\alpha \right]}_{D}^{23}$ +18 (*c* 0.11 MeOH); UV *λ*_max_ 212, 264 and 302 nm; ECD {MeCN, λ [nm] (Δε), c = 2.27 × 10^−4^ M} 306 (+1.11), 286sh (+0.15), 261 (−5.71), 228sh (−1.99), 212 (+5.82); ^1^H and ^13^C NMR, see Table 2; ESI-HRMS *m*/*z* 309.1333 [M + H]^+^ (calcd. for C_16_H_21_O_6_, 309.1333).

*(3S,7R)-7-Hydroxyresorcylide* (**8**): brown amorphous solid; ${\left[\alpha \right]}_{D}^{23}$ −8 (*c* 0.13 MeOH); UV *λ*_max_ 213, 263 and 302 nm; ECD {MeCN, λ [nm] (Δε), c = 3.24 × 10^−4^ M} 308 (+0.28), 285sh (−0.54), 260 (−3.33), 228sh (−1.81), 211 (+5.46); ^1^H and ^13^C NMR, see Table 2; ESI-HRMS *m*/*z* 309.1327 [M + H]^+^ (calcd. for C_16_H_21_O_6_, 309.1333).

*(3S,7S)-7-Methoxyresorcylide* (**9**): brown amorphous solid; ${\left[\alpha \right]}_{D}^{23}$ +4 (*c* 0.13, MeOH); UV *λ*_max_ 213, 263 and 304 nm; ECD {MeCN, λ [nm] (Δε), c = 3.72 × 10^−4^ M} 302 (+1.62), 284sh (+0.78), 260 (−6.22), 228sh (−1.53), 212 (+3.34), 191 (−5.60); ^1^H and ^13^C NMR, see Table 3; ESI-HRMS *m*/*z* 323.1495 [M + H]^+^ (calcd. for C_17_H_23_O_6_, 323.1489.

*(3S,7R)-7-Methoxyresorcylide* (**10**): brown amorphous solid; ${\left[\alpha \right]}_{D}^{23}$ −8 (*c* 0.12, MeOH); UV *λ*_max_ 213, 265 and 298 nm; ECD {MeCN, λ [nm] (Δε), c = 1.86 × 10^−4^ M} 308 (+0.50), 287sh (−0.79), 260 (−5.47), 228sh (−2.77), 211 (+9.62); ^1^H and ^13^C NMR, see Table 3; ESI-HRMS *m*/*z* 323.1493 [M + H]^+^ (calcd. for C_17_H_23_O_6_, 323.1489).

*(3S,7S)-7-O-n-Butylresorcylide* (**11**): brown amorphous solid; ${\left[\alpha \right]}_{D}^{23}$ +5 (*c* 0.20, MeOH); UV *λ*_max_ 213, 265 and 303 nm; ECD {MeCN, λ [nm] (Δε), c = 1.58 × 10^−4^ M} 302 (+6.88), 284sh (+3.87), 261 (−21.91), 230sh (−5.75), 212 (+16.12), 191 (−22.28); ^1^H and ^13^C NMR, see Table 4; ESI-HRMS *m*/*z* 365.1964 [M + H]^+^ (calcd. for C_20_H_29_O_6_, 365.1959).

*(3S,7R)-7-O-n-Butylresorcylide* (**12**): brown amorphous solid; ${\left[\alpha \right]}_{D}^{23}$ −17 (*c* 0.20, MeOH); UV *λ*_max_ 213, 264 and 303 nm; ECD {MeCN, λ [nm] (Δε), c = 2.88 × 10^−4^ M} 309 (+0.23), 287sh (−0.74), 260 (−4.38), 227sh (−2.93), 210 (+8.23); ^1^H and ^13^C NMR, see Table 4; ESI-HRMS *m*/*z* 365.1961 [M + H]^+^ (calcd. for C_20_H_29_O_6_, 365.1959).

*Talarodilactone A* (**13**): brown amorphous solid; ${\left[\alpha \right]}_{D}^{23}$ +9 (*c* 0.21, MeOH); UV *λ*_max_ 212, 269 and 291 nm; ECD {MeCN, λ [nm] (Δε), c = 1.68 × 10^−4^ M} 296sh (+1.05), 284 (+1.11), 260 (−4.22), 226sh (−1.51), 209 (+3.86); ^1^H and ^13^C NMR, see Table 6; ESI-HRMS *m*/*z* 619.1794 [M + Na]^+^ (calcd. for C_31_H_32_O_12_Na, 619.1786).

*Talarodilactone B* (**14**): brown amorphous solid; ${\left[\alpha \right]}_{D}^{23}$ −22 (*c* 0.21, MeOH); UV *λ*_max_ 212, 268 and 291 nm; ECD {MeCN, λ [nm] (Δε), c = 2.49 × 10^−4^ M} 298 (+0.79), 285sh (+0.31), 259 (−5.69), 235 (+0.25), 225 (−4.24), 208 (+11.95); ^1^H and ^13^C NMR, see Table 6; ESI-HRMS *m*/*z* 605.1625 [M + Na]^+^ (calcd. for C_30_H_30_O_12_Na, 605.1629).

*Talumarin A* (**15**): brown amorphous solid; ${\left[\alpha \right]}_{D}^{23}$ −18 (*c* 0.27, CHCl_3_); UV *λ*_max_ 220, 258 and 334 nm; ECD {MeCN, λ [nm] (Δε), c = 9.91 × 10^−5^ M} 324 (−0.35), 263 (−1.36), 242 (+1.20), 226sh (+0.75), 207 (−5.24); ^1^H and ^13^C NMR, see Table 7; ESI-HRMS *m*/*z* 275.0528 [M + Na]^+^ (calcd. for C_12_H_12_O_6_Na, 275.0526).

*Talumarin B* (**16**): brown amorphous solid; ${\left[\alpha \right]}_{D}^{23}$ −32 (*c* 0.27, CHCl_3_); UV *λ*_max_ 221, 258 and 330 nm; ECD {MeCN, λ [nm] (Δε), c = 7.09 × 10^−5^ M} 327 (−0.26), 262 (−1.22), 243 (+0.75), 226sh (+0.17), 206 (−3.57); ^1^H and ^13^C NMR, see Table 7; ESI-HRMS *m*/*z* 331.1151 [M + Na]^+^ (calcd. for C_16_H_20_O_6_Na, 331.1151).

### 4.4. Computational Section

Geometry optimizations (B3LYP/6-31G(d) in vacuo, B97D/TZVP [32,33] and CAM-B3LYP/TZVP [34] with PCM solvent model for MeCN), and TDDFT calculations were performed with Gaussian 09 using various functionals (B3LYP, BH&HLYP, CAM-B3LYP and PBE0) and the TZVP basis set [35]. ECD spectra were generated as the sum of Gaussians with 2400 and 3000 cm^−1^ half-height width (corresponding to c.a. 14 and 17 nm at 240 nm), using dipole-velocity computed rotational strengths [36]. Mixed torsional/low mode conformational searches were carried out by means of the Macromodel 10.8.011 software (New York, NY, USA) using Merck Molecular Force Field (MMFF) with implicit solvent model for CHCl_3_ applying a 21 kJ/mol energy window [37]. Boltzmann distributions were estimated from the B3LYP, B97D and CAM-B3LYP energies. In the case of the B3LYP/6-31G(d) in vacuo level, ZPVE corrections were applied. The MOLEKEL software package (New York, NY, USA) was used for visualization of the results [38].

### 4.5. Cytotoxicity Assay

Cytotoxicity against the L5178Y murine lymphoma cell line was tested using the MTT (3-(4,5-dimethylthiazol-2-yl)-2,5-diphenyltetrazolium bromide) method. The depsipeptide kahalalide F was used as positive control [39].

### 4.6. Antimicrobial Assay

The inoculum was obtained by direct colony suspension preparation. Antibacterial activities against *Mycobacterium tuberculosis* (H37Rv), *Staphylococcus aureus* (ATCC 25923) and *Acinetobacter baumannii* (ATCC BAA-1605) were evaluated by broth micro dilution methodology as stated by the recommendations of the Clinical and Laboratory Standards Institute (CLSI) [40].

## Figures and Tables

**Table 1:** ^1^H and ^13^C NMR (Nuclear Magnetic Resonance) Data for compounds **1**–**3**.

No.	1 *^a^*		2 *^a^*		3 *^a^*	
	*δ*_C,_ Type	*δ*_H_ (*J* in Hz)	*δ*_C,_ Type	*δ*_H_ (*J* in Hz)	*δ*_C,_ Type	*δ*_H_ (*J* in Hz)
1	171.0, C		168.3, C		170.0, C	
2	139.5, C		142.7, C		141.6, C	
3	130.6, C		124.6, C		128.7, C	
4	79.3, CH	5.52, dd (8.1, 2.2)	109.8, C		86.8, C	
5	30.5, CH_2_	2.45, m	34.6, CH_2_	2.54, ddd (14.5, 7.9, 6.5)	30.0, CH_2_	2.68, ddd (15.2, 8.7, 6.4)
		1.75, m		2.18, ddd (14.5, 7.9, 6.5)		2.48, ddd (15.2, 8.7, 6.4)
6	30.0, CH_2_	2.44, m	29.4, CH_2_	2.42, ddd (16.0, 7.9, 6.5)	29.1, CH_2_	2.25, ddd (15.8, 8.7, 6.4)
				2.31, ddd (16.0, 7.9, 6.5)		2.15, ddd (15.8, 8.7, 6.4)
7	174.6, C		174.5, C		174.2, C	
8	131.9, C		131.3, C		131.3, C	
9, 13	128.6, CH	7.75, d (7.5)	128.6, CH	7.93, d (7.5)	128.6, CH	7.76, d (7.4)
10, 12	129.7, CH	7.44, t (7.5)	129.8, CH	7.45, t (7.5)	129.7, CH	7.42, t (7.4)
11	129.7, CH	7.35, t (7.5)	129.8, CH	7.36, t (7.5)	129.9, CH	7.35, t (7.4)
14					171.9, C	
1′	65.6, CH_2_	4.08, dt (10.8, 6.6)	65.6, CH_2_	4.02, dt (10.8, 6.7)	65.7, CH_2_	4.00, dt (10.8, 6.7)
		4.04, dt (10.8, 6.6)		3.97, dt (10.8, 6.7)		3.94, dt (10.8, 6.7)
2′	31.7, CH_2_	1.60, m	31.7, CH_2_	1.56, m	31.6, CH_2_	1.54, m
3′	20.2, CH_2_	1.38, m	20.2, CH_2_	1.36, m	20.1, CH_2_	1.34, m
4′	14.0, CH_3_	0.94, t (7.3)	14.0, CH_3_	0.93, t (7.3)	14.0, CH_3_	0.91, t (7.3)
OMe-4			50.7, CH_3_	3.18, s		

*^a^* Recorded at 300 MHz (^1^H) and 75 MHz (^13^C) in CD_3_OD.

**Table 2:** ^1^H and ^13^C NMR Data for compounds **6**–**8**.

No.	6 *^a^*		7 *^b^*		8 *^b^*	
	*δ*_C,_ Type	*δ*_H_ (*J* in Hz)	*δ*_C,_ Type *^c^*	*δ*_H_ (*J* in Hz)	*δ*_C,_ Type *^c^*	*δ*_H_ (*J* in Hz)
1	172.9, C		n.d. *^d^*		n.d. *^d^*	
3	76.9, CH	5.03, m	75.5, CH	4.95, m	73.1, CH	5.19, m
4	32.2, CH_2_	1.87, m	33.1, CH_2_	1.91, m	32.9, CH_2_	1.68, m
		1.65, m		1.75, m		
5	26.4, CH_2_	1.68, m	21.5, CH_2_	1.70, m	18.9, CH_2_	1.58, m
				1.43, m		
6	27.6, CH_2_	2.52, m	36.5, CH_2_	1.58, m	36.8, CH_2_	1.81, m
		2.21, m				1.50, m
7	139.9, CH	5.81, ddd (11.8, 10.0, 5.7)	66.2, CH	4.39, m	66.9, CH	4.36, m
8	132.8, CH	6.51, br d (11.8)	53.5, CH_2_	3.03, dd (13.0, 3.1)	51.3, CH_2_	2.94, dd (15.4, 10.1)
				2.42, dd (13.0, 10.3)		2.61, dd (15.4, 1.6)
9	204.7, C		206.3, C		205.2, C	
10	51.5, CH_2_	4.58, d (18.4)	50.2, CH_2_	4.84, d (18.4)	52.3, CH_2_	4.59, d (18.5)
		3.65, d (18.4)		3.75, d (18.4)		3.90, d (18.5)
11	140.1, C		140.0, C		140.2, C	
12	113.9, CH	6.16, d (2.5)	113.5, CH	6.23, d (2.5)	113.2, CH	6.23, d (2.5)
13	164.1, C		162.7, C		162.9, C	
14	103.0, CH	6.26, d (2.5)	102.2, CH	6.31, d (2.5)	102.3, CH	6.32, d (2.5)
15	167.1, C		165.5, C		166.4, C	
16	106.2, C		106.3, C		105.6, C	
17	21.5, CH_3_	1.28, d (6.2)	20.9, CH_3_	1.31, d (6.1)	18.2, CH_3_	1.29, d (6.4)
7-OMe						

*^a^* Recorded at 600 MHz (^1^H) and 150 MHz (^13^C) in CD_3_OD. *^b^* Recorded at 600 MHz (^1^H) and 150 MHz (^13^C) in CD_3_COCD_3_. *^c^* Data extracted from HSQC (Heteronuclear Single Quantum Correlation) and HMBC. *^d^* n.d. = not detected.

**Table 3:** ^1^H and ^13^C NMR Data for compounds **9** and **10**.

No.	9 *^a^*		10 *^a^*	
	*δ*_C,_ Type	*δ*_H_ (*J* in Hz)	*δ*_C,_ Type	*δ*_H_ (*J* in Hz)
1	172.5, C		172.3, C	
3	76.1, CH	4.93, m	73.8, CH	5.19, m
4	33.8, CH_2_	1.82, m	33.5, CH_2_	1.63, m
		1.75, m		
5	21.7, CH_2_	1.68, m	19.0, CH_2_	1.58, m
		1.40, m		
6	34.4, CH_2_	1.69, m	34.0, CH_2_	1.87, m
		1.50, m		1.49, m
7	77.1, CH	3.95, m	78.2, CH	3.90, m
8	49.6, CH_2_	3.10, dd (13.3, 2.8)	47.4, CH_2_	2.89, dd (15.7, 9.7)
		2.40, dd (13.3, 10.2)		2.71, dd (15.7, 1.0)
9	208.8, C		208.1, C	
10	51.0, CH_2_	4.68, d (18.7)	53.2, CH_2_	4.63, d (18.8)
		3.80, d (18.7)		3.84, d (18.8)
11	139.6, C		139.9, C	
12	113.9, CH	6.14, d (2.5)	113.7, CH	6.13, d (2.5)
13	163.7, C		163.9, C	
14	102.9, CH	6.25, d (2.5)	103.0, CH	6.25, d (2.5)
15	166.0, C		166.9, C	
16	107.0, C		106.4, C	
17	21.1, CH_3_	1.32, d (6.1)	18.5, CH_3_	1.29, d (6.4)
7-OMe	56.7, CH_3_	3.42, s	56.5, CH_3_	3.34, s

*^a^* Recorded at 600 MHz (^1^H) and 150 MHz (^13^C) in CD_3_OD.

**Table 4:** ^1^H and ^13^C NMR Data for compounds **11** and **12**.

No.	11 *^a^*		12 *^b^*	
	*δ*_C,_ Type	*δ*_H_ (*J* in Hz)	*δ*_C,_ Type	*δ*_H_ (*J* in Hz)
1	172.5, C		172.3, C	
3	76.2, CH	4.94, m	73.9, CH	5.19, m
4	33.8, CH_2_	1.80, m	33.6, CH_2_	1.64, m
		1.75, m		
5	21.9, CH_2_	1.69, m	19.1, CH_2_	1.58, m
		1.40, m		
6	34.6, CH_2_	1.66, m	34.3, CH_2_	1.84, m
		1.50, m		1.51, m
7	75.4, CH	4.04, m	76.5, CH	3.98, m
8	50.3, CH_2_	3.09, dd (13.2, 3.0)	48.2, CH_2_	2.90, dd (15.6, 9.8)
		2.40, dd (13.2, 10.3)		2.69, dd (15.6, 1.0)
9	208.9, C		208.3, C	
10	50.9, CH_2_	4.69, d (18.7)	53.1, CH_2_	4.61, d (18.8)
		3.78, d (18.7)		3.84, d (18.8)
11	139.6, C		139.9, C	
12	114.0, CH	6.13, d (2.5)	113.8, CH	6.12, d (2.5)
13	163.7, C		163.9, C	
14	102.9, CH	6.25, d (2.5)	103.1, CH	6.25, d (2.5)
15	166.0, C		166.8, C	
16	107.0, C		106.5, C	
17	21.1, CH_3_	1.31, d (6.1)	18.6, CH_3_	1.29, d (6.4)
1′	69.7, CH_2_	3.63, dt (9.2, 6.4)	69.5, CH_2_	3.51, dt (9.3, 6.5)
		3.51, dt (9.2, 6.4)		3.45, dt (9.3, 6.5)
2′	33.2, CH_2_	1.56, m	33.2, CH_2_	1.53, m
3′	20.4, CH_2_	1.42, m	20.4, CH_2_	1.38, m
4′	14.3, CH_3_	0.95, t (7.3)	14.2, CH_3_	0.93, t (7.3)

*^a^* Recorded at 300 MHz (^1^H) and 75 MHz (^13^C) in CD_3_OD. *^b^* Recorded at 500 MHz (^1^H) and 125 MHz (^13^C) in CD_3_OD.

**Table 5:** Key differences of NMR Data for compounds **7**–**14**.

Compounds	7	9	11	13	8	10	12	14
^3^*J*_7,8a_ (Hz)	3.1	2.8	3.0	3.1	10.1	9.6	9.8	10.4
^3^*J*_7,8b_ (Hz)	10.3	10.2	10.3	10.4	1.6	1.0	1.0	- *^a^*
C-3	75.5	76.1	76.2	76.1	73.1	73.8	73.9	74.0
H-3	4.95	4.93	4.94	4.93	5.19	5.19	5.19	5.13
C-17	20.9	21.1	21.1	20.9	18.2	18.5	18.6	18.9

*^a^* Signals overlapped.

**Table 6:** ^1^H and ^13^C NMR Data for compounds **13** and **14**.

No.	13 *^a^*		14 *^a^*	
	*δ*_C,_ Type	*δ*_H_ (*J* in Hz)	*δ*_C,_ Type	*δ*_H_ (*J* in Hz)
1	172.4, C		172.2, C	
3	76.1, CH	4.93, m	74.0, CH	5.13, m
4	33.4, CH_2_	1.75, m	33.8, CH_2_	1.66, m
		1.61, m		
5	21.2, CH_2_	1.39, m	19.0, CH_2_	1.52, m
		1.31, m		
6	33.2, CH_2_	1.57, m	32.8, CH_2_	1.84, m
				1.56, m
7	72.9, CH	5.54, m	74.2, CH	5.53, m
8	49.2, CH_2_	2.94, dd (13.3, 3.1)	46.9, CH_2_	3.07, dd (15.8, 10.4)
		2.58, dd (13.3, 10.4)		2.49, d (15.8)
9	206.6, C		206.1, C	
10	50.8, CH_2_	4.67, d (18.8)	52.8, CH_2_	4.52, d (18.8)
		3.78, d (18.8)		3.73, d (18.8)
11	139.3, C		139.6, C	
12	114.1, CH	6.14, d (2.4)	113.8, CH	6.07, d (2.5)
13	163.8, C		163.9, C	
14	103.0, CH	6.26, d (2.4)	103.0, CH	6.24, d (2.5)
15	166.2, C		166.8, C	
16	106.7, C		106.2, C	
17	20.9, CH_3_	1.28, d (6.2)	18.9, CH_3_	1.27, d (6.4)
1′	169.8, C		169.7, C	
2′	141.9, C		141.8, C	
3′	128.1, C		128.6, C	
4′	86.6, C		86.6, CH	
5′	30.1, CH_2_	2.77, ddd (15.2, 8.6, 6.4)	30.0, CH_2_	2.72, ddd (15.3, 8.7, 6.5)
		2.59, ddd (15.2, 8.6, 6.4)		2.49, ddd (15.3, 8.7, 6.5)
6′	28.7, CH_2_	2.29, ddd (16.0, 8.6, 6.4)	28.7, CH_2_	2.23, ddd (15.9, 8.7, 6.5)
		2.22, ddd (16.0, 8.6, 6.4)		2.14, ddd (15.9, 8.7, 6.5)
7′	174.4, C		175.9, C	
8′	131.0, C		131.0, C	
9′, 13′	128.6, CH	7.73, d (7.7)	128.5, CH	7.70, d (7.4)
10′, 12′	129.9, CH	7.44, t (7.7)	129.9, CH	7.44, t (7.4)
11′	130.1, CH	7.37, t (7.7)	130.1, CH	7.37, t (7.4)
14′	169.9, C		169.5, C	
OMe	52.3, C	3.58, s		

*^a^* Recorded at 700 MHz (^1^H) and 175 MHz (^13^C) in CD_3_OD.

**Table 7:** ^1^H and ^13^C NMR Data for compounds **15** and **16**.

No.	15 *^a^*		16 *^b^*	
	*δ*_C,_ Type	*δ*_H_ (*J* in Hz)	*δ*_C,_ Type	*δ*_H_ (*J* in Hz)
1	169.6, C		169.5, C	
3	79.3, CH	4.63, m	80.8, CH	4.63, m
4	31.2, CH_2_	2.94, dd (16.4, 3.6)	32.7, CH_2_	2.96, dd (16.1, 4.1)
		2.83, dd (16.4, 10.9)		2.87, dd (16.1, 10.4)
5	117.5, CH	6.63, d (8.0)	118.6, CH	6.65, d (8.0)
6	121.6, CH	7.01, d (8.0)	122.4, CH	7.01, d (8.0)
7	144.4, C		145.7, C	
8	149.9, C		151.0, C	
9	108.4, C		109.4, C	
10	129.1, C		130.7, C	
11	29.3, CH_2_	1.96, m	30.7, CH_2_	2.09, m
12	29.1, CH_2_	2.42, m	30.3, CH_2_	2.58, m
13	173.8, C		174.4, C	
1′			65.3, CH_2_	4.11, t (6.6)
2′			31.6, CH_2_	1.63, m
3′			19.9, CH_2_	1.40, m
4′			13.7, CH_3_	0.95, t (7.4)
OH-7		9.33, s		
OH-8		10.82, s		
OH-13		12.21, s		

*^a^* Recorded at 300 MHz (^1^H) and 75 MHz (^13^C) in DMSO-*d*_6_. *^b^* Recorded at 300 MHz (^1^H) and 75 MHz (^13^C) in CD_3_OD.

## Supplementary Materials

The following are available online at www.mdpi.com/1660-3397/15/11/359/s1, ESI-HRMS, 1D and 2D NMR spectra of all the new compounds as well as ECD spectra of compounds **1**, **2**, **5**–**16** and ECD calculations for compound **9**.
