# Supplementary material for: Lactones from the Sponge-Derived Fungus Talaromyces rugulosus
Source: Mar Drugs. 2017 Nov 14;15(11):359. doi: 10.3390/md15110359 (PMC5706048; doi:10.3390/md15110359)
Supplement: Supplementary file 1 [file marinedrugs-15-00359-s001.pdf]

Supplementary Material

Lactones from the Sponge-Derived Fungus *Talaromyces rugulosus*

Lisa Küppers [1,§], Weaam Ebrahim [1,2,§], Mona El-Neketi [2], Ferhat Can Özkaya [1], Attila Mándi [3], Tibor Kurtán [3], Raha S. Orfali [4], Werner E. G. Müller [5], Rudolf Hartmann [6], Wenhan Lin [7], Weiguo Song [8], Zhen Liu [1,*], Peter Proksch [1,*]

[1] Institute of Pharmaceutical Biology and Biotechnology, Heinrich-Heine-Universität Düsseldorf, 40225 Düsseldorf, Germany; li-kue@gmx.de (L.K.); weaam.ebrahim@uni-duesseldorf.de (W.E.); fcanozkaya@gmail.com (F.C.Ö.)

[2] Department of Pharmacognosy, Faculty of Pharmacy, Mansoura University, Mansoura 35516, Egypt; monaneketi@yahoo.com (M.E.-N.)

[3] Department of Organic Chemistry, University of Debrecen, Debrecen 4032, Hungary; mandi.attila@science.unideb.hu (A.M.); kurtan.tibor@science.unideb.hu (T.K.)

[4] Department of Pharmacognosy, Faculty of Pharmacy, King Saud University, Riyadh, Saudi Arabia; rom2leen@gmail.com (R.S.O.)

[5] Institute of Physiological Chemistry, Universitätsmedizin der Johannes Gutenberg-Universität Mainz, 55128 Mainz, Germany; wmueller@uni-mainz.de (W.E.G.M.)

[6] Institute of Complex Systems: Strukturbiochemie, Forschungszentrum Juelich, 52428 Juelich, Germany; r.hartmann@fz-juelich.de (R.H.)

[7] State Key Laboratory of Natural and Biomimetic Drugs, Peking University, Beijing 100191, People's Republic of China; whlin@bjmu.edu.cn (W.L.)

[8] Fukang Pharma, North-East of Dongwaihuan Road, Dongcheng Industrial Area, 262700 Shouguang City, People's Republic of China; songwg@139.com (W.S.)

* Correspondence: zhenfeizi0@sina.com (Z.L.); proksch@uni-duesseldorf (P.P.); Tel.: +49-211-81-14163

§ L. Küppers and W. Ebrahim contributed equally to this study

Table of Contents

	Page
**S1.** HRESIMS spectrum of compound **1**.	6
**S2.** ^{1}H NMR (300 MHz, CD_3OD) spectrum of compound **1**.	7
**S3.** ^{13}C NMR (75 MHz, CD_3OD) spectrum of compound **1**.	8
**S4**. ^{1}H-^{1}H COSY (300 MHz, CD_3OD) spectrum of compound **1**.	9
**S5**. HSQC (300 and 75 MHz, CD_3OD) spectrum of compound **1**.	10
**S6**. HMBC (300 and 75 MHz, CD_3OD) spectrum of compound **1**.	11
**S7**. HRESIMS spectrum of compound **2**.	12
**S8.** ^{1}H NMR (300 MHz, CD_3OD) spectrum of compound **2**.	13
**S9.** ^{13}C NMR (75 MHz, CD_3OD) spectrum of compound **2**.	14
**S10.** ^{1}H-^{1}H COSY (300 MHz, CD_3OD) spectrum of compound **2**.	15
**S11.** HSQC (300 and 75 MHz, CD_3OD) spectrum of compound **2**.	16
**S12**. HMBC (300 and 75 MHz, CD_3OD) spectrum of compound **2**.	17
**S13**. HRESIMS spectrum of compound **3**.	18
**S14**. ^{1}H NMR (300 MHz, CD_3OD) spectrum of compound **3**.	19
**S15**. ^{13}C NMR (75 MHz, CD_3OD) spectrum of compound **3**.	20
**S16.** ^{1}H-^{1}H COSY (300 MHz, CD_3OD) spectrum of compound **3**.	21
**S17.** HSQC (300 and 75 MHz, CD_3OD) spectrum of compound **3**.	22
**S18.** HMBC (300 and 75 MHz, CD_3OD) spectrum of compound **3**.	23
**S19.** HRESIMS spectrum of compound **6**.	24
**S20**. ^{1}H NMR (600 MHz, CD_3OD) spectrum of compound **6**.	25
**S21**. ^{13}C NMR (150 MHz, CD_3OD) spectrum of compound **6**.	26
**S22**. ^{1}H-^{1}H COSY (600 MHz, CD_3OD) spectrum of compound **6**.	27
**S23.** HSQC (600 and 150 MHz, CD_3OD) spectrum of compound **6**.	28
**S24.** HMBC (600 and 150 MHz, CD_3OD) spectrum of compound **6**.	29
**S25.** ROESY (600 MHz, CD_3OD) spectrum of compound **6**.	30
**S26.** HRESIMS spectrum of compound **7**.	31
**S27.** ^{1}H NMR (600 MHz, CD_3OD) spectrum of compound**7**.	32
**S28.** ^{1}H NMR (600 MHz, CD_3COCD_3) spectrum of compound **7**.	33

S29. ^{1}H-^{1}H COSY (600 MHz, CD_3COCD_3) spectrum of compound **7**. 34
S30. HSQC (600 and 150 MHz, CD_3COCD_3) spectrum of compound **7**. 35
S31. HMBC (600 and 150 MHz, CD_3COCD_3) spectrum of compound **7**. 36
S32. ROESY (600 MHz, CD_3COCD_3) spectrum of compound **7** 37
S33. HRESIMS spectrum of compound **8**. 38
S34. ^{1}H NMR (600 MHz, CD_3COCD_3) spectrum of compound **8**. 39
S35. ^{1}H NMR (600 MHz, CD_3OD) spectrum of compound **8**. 40
S36. ^{1}H-^{1}H COSY (600 MHz, CD_3COCD_3) spectrum of compound **8**. 41
S37. HSQC (600 and 150 MHz, CD_3COCD_3) spectrum of compound **8**. 42
S38. HMBC (600 and 150 MHz, CD_3COCD_3) spectrum of compound **8**. 43
S39. ROESY (600 MHz, CD_3COCD_3) spectrum of compound **8**. 44
S40. HRESIMS spectrum of compound **9**. 45
S41. ^{1}H NMR (600 MHz, CD_3OD) spectrum of compound **9**. 46
S42. ^{1}H-^{1}H COSY (600 MHz, CD_3OD) spectrum of compound **9**. 47
S43. HSQC (600 and 150 MHz, CD_3OD spectrum of compound **9**. 48
S44. HMBC (600 and 150 MHz, CD_3OD) spectrum of compound **9**. 49
S45. ROESY (600 MHz, CD_3OD) spectrum of compound **9**. 50
S46. HRESIMS spectrum of compound **10**. 51
S47. ^{1}H NMR (600 MHz, CD_3OD) spectrum of compound **10**. 52
S48. ^{1}H-^{1}H COSY (600 MHz, CD_3OD) spectrum of compound **10**. 53
S49. HSQC (600 and 150 MHz, CD_3OD spectrum of compound **10**. 54
S50. HMBC (600 and 150 MHz, CD_3OD) spectrum of compound **10**. 55
S51. ROESY (600 MHz, CD_3OD) spectrum of compound **10**. 56
S52. HRESIMS spectrum of compound **11**. 57
S53. ^{1}H NMR (300 MHz, CD_3OD) spectrum of compound **11**. 58
S54. ^{13}C NMR (75 MHz, CD_3OD) spectrum of compound **11**. 59
S55. ^{1}H-^{1}H COSY (300 MHz, CD_3OD) spectrum of compound **11**. 60
S56. HSQC (300 and 75 MHz, CD_3OD spectrum of compound **11**. 61
S57. HMBC (300 and 75 MHz, CD_3OD) spectrum of compound **11**. 62
S58. ROESY (300 MHz, CD_3OD) spectrum of compound **11**. 63
S59. HRESIMS spectrum of compound **12**. 64

S60. ^{1}H NMR (500 MHz, CD_3OD) spectrum of compound **12**. 65
S61. ^{13}C NMR (125 MHz, CD_3OD) spectrum of compound **12**. 66
S62. ^{1}H-^{1}H COSY (500 MHz, CD_3OD) spectrum of compound **12**. 67
S63. HSQC (500 and 125 MHz, CD_3OD spectrum of compound **12**. 68
S64. HMBC (500 and 125 MHz, CD_3OD) spectrum of compound **12**. 69
S65. ROESY (500 MHz, CD_3OD) spectrum of compound **12**. 70
S66. HRESIMS spectrum of compound **13**. 71
S67. ^{1}H NMR (700 MHz, CD_3OD) spectrum of compound **13**. 72
S68. ^{13}C NMR (175 MHz, CD_3OD) spectrum of compound **13**. 73
S69. ^{1}H-^{1}H TOCSY NMR (700 MHz, CD_3OD) spectrum of compound **13**. 74
S70. HSQC (700 and 175 MHz, CD_3OD) spectrum of compound **13**. 75
S71. HMBC (700 and 175 MHz, CD_3OD) spectrum of compound **13**. 76
S72. ROESY (700 MHz, CD_3OD) spectrum of compound **13**. 77
S73. HRESIMS spectrum of compound **14**. 78
S74. ^{1}H NMR (700 MHz, CD_3OD) spectrum of compound **14**. 79
S75. ^{13}C NMR (175 MHz, CD_3OD) spectrum of compound **14**. 80
S76. ^{1}H-^{1}H TOCSY NMR (700 MHz, CD_3OD) spectrum of compound **14**. 81
S77. HSQC (700 and 175 MHz, CD_3OD) spectrum of compound **14**. 82
S78. HMBC (700 and 175 MHz, CD_3OD) spectrum of compound **14**. 83
S79. ROESY (700 MHz, CD_3OD) spectrum of compound **14**. 84
S80. HRESIMS spectrum of compound **15**. 85
S81. ^{1}H NMR (300 MHz, CD_3OD) spectrum of compound **15**. 86
S82. ^{1}H NMR (300 MHz, DMSO-d_6) spectrum of compound **15**. 87
S83. ^{13}C NMR (75 MHz, DMSO-d_6) spectrum of compound **15**. 88
S84. ^{1}H-^{1}H COSY (300 MHz, DMSO-d_6) spectrum of compound **15**. 89
S85. HSQC (300 and 75 MHz, DMSO-d_6) spectrum of compound **15**. 90
S86. HMBC (300 and 75 MHz, DMSO-d_6) spectrum of compound **15**. 91
S87. HRESIMS spectrum of compound **16**. 92
S88. ^{1}H NMR (300 MHz, CD_3OD) spectrum of compound **16**. 93
S89. ^{1}H-^{1}H COSY (300 MHz, CD_3OD) spectrum of compound **16**. 94
S90. HSQC (300 and 75 MHz, CD_3OD) spectrum of compound **16**. 95

S91. HMBC (300 and 75 MHz, CD_3OD) spectrum of compound **16**. 96
S92. Experimental ECD spectrum of **9** in MeCN compared with the Boltzmann-weighted B3LYP/TZVP PCM/MeCN ECD spectrum of (3*S*,7*S*)-**9** computed for the B97D/TZVP PCM/MeCN conformers. 97
S93. Experimental ECD spectrum of **1** in MeCN. 98
S94. Experimental ECD spectrum of **2** in MeCN. 99
S95. Experimental ECD spectrum of **5** in MeCN. 100
S96. Experimental ECD spectrum of **6** in MeCN. 101
S97. Experimental ECD spectrum of **7** in MeCN. 102
S98. Experimental ECD spectrum of **8** in MeCN. 103
S99. Experimental ECD spectrum of **9** in MeCN. 104
S100. Experimental ECD spectrum of **10** in MeCN. 105
S101. Experimental ECD spectrum of **11** in MeCN. 106
S102. Experimental ECD spectrum of **12** in MeCN. 107
S103. Experimental ECD spectrum of **13** in MeCN. 108
S104. Experimental ECD spectrum of **14** in MeCN. 109
S105. Experimental ECD spectrum of **15** in MeCN. 110
S106. Experimental ECD spectrum of **16** in MeCN. 111

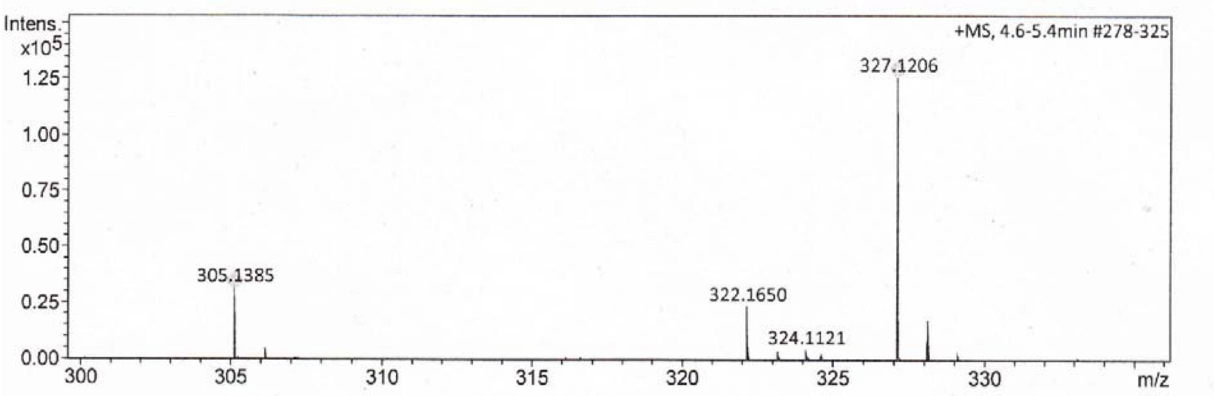

Meas. m/z	#	Ion Formula	m/z	err [ppm]	mSigma	# mSigma	Score	rdb	e⁻ Conf	N-Rule
305.1385	1	C17H21O5	305.1384	-0.5	14.0	1	100.00	7.5	even	ok
327.1206	1	C17H20NaO5	327.1203	-0.9	28.8	1	100.00	7.5	even	ok

S1. HRESIMS spectrum of compound **1**.

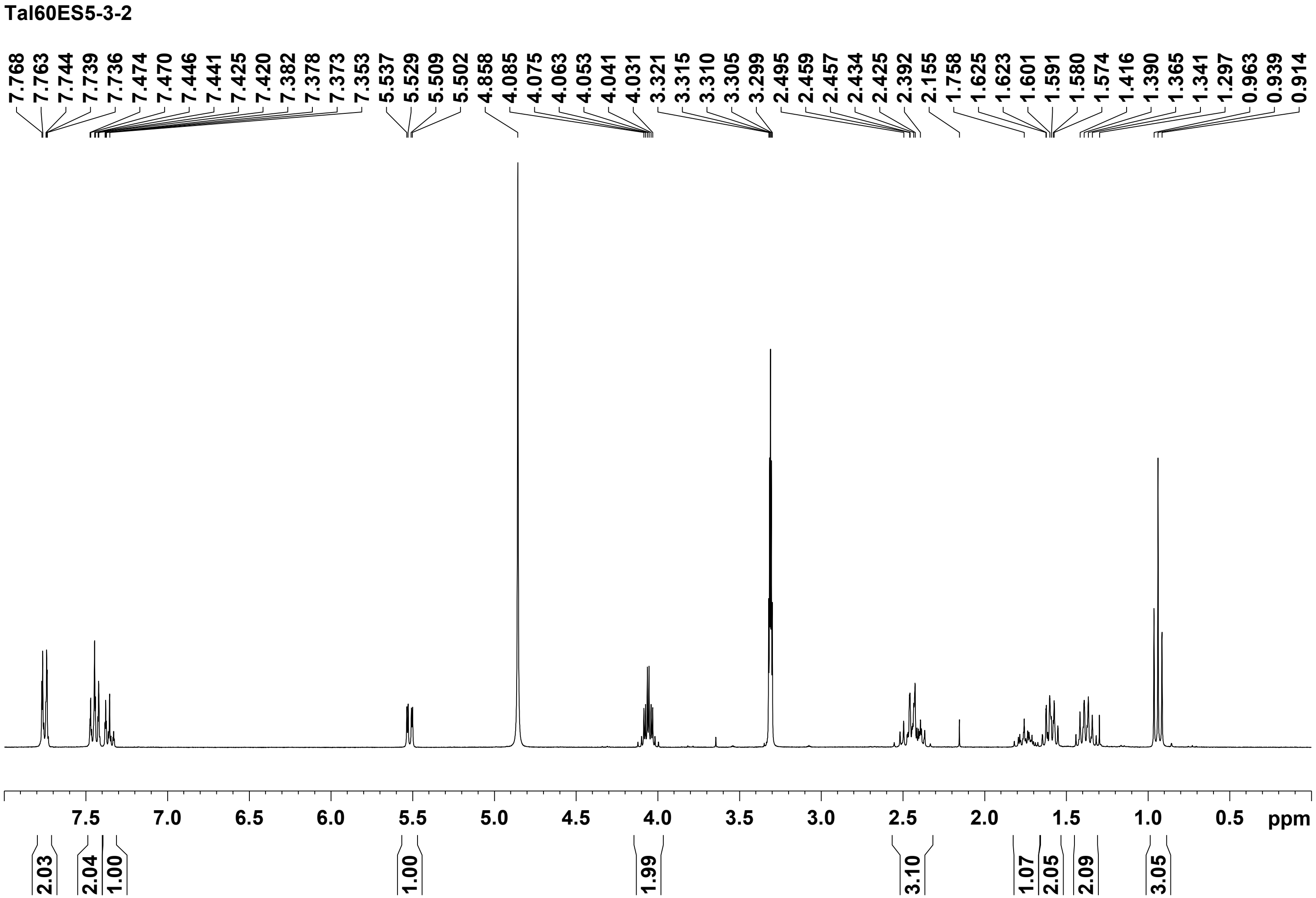

S2. ^{1}H NMR (300 MHz, CD_3OD) spectrum of compound **1**.

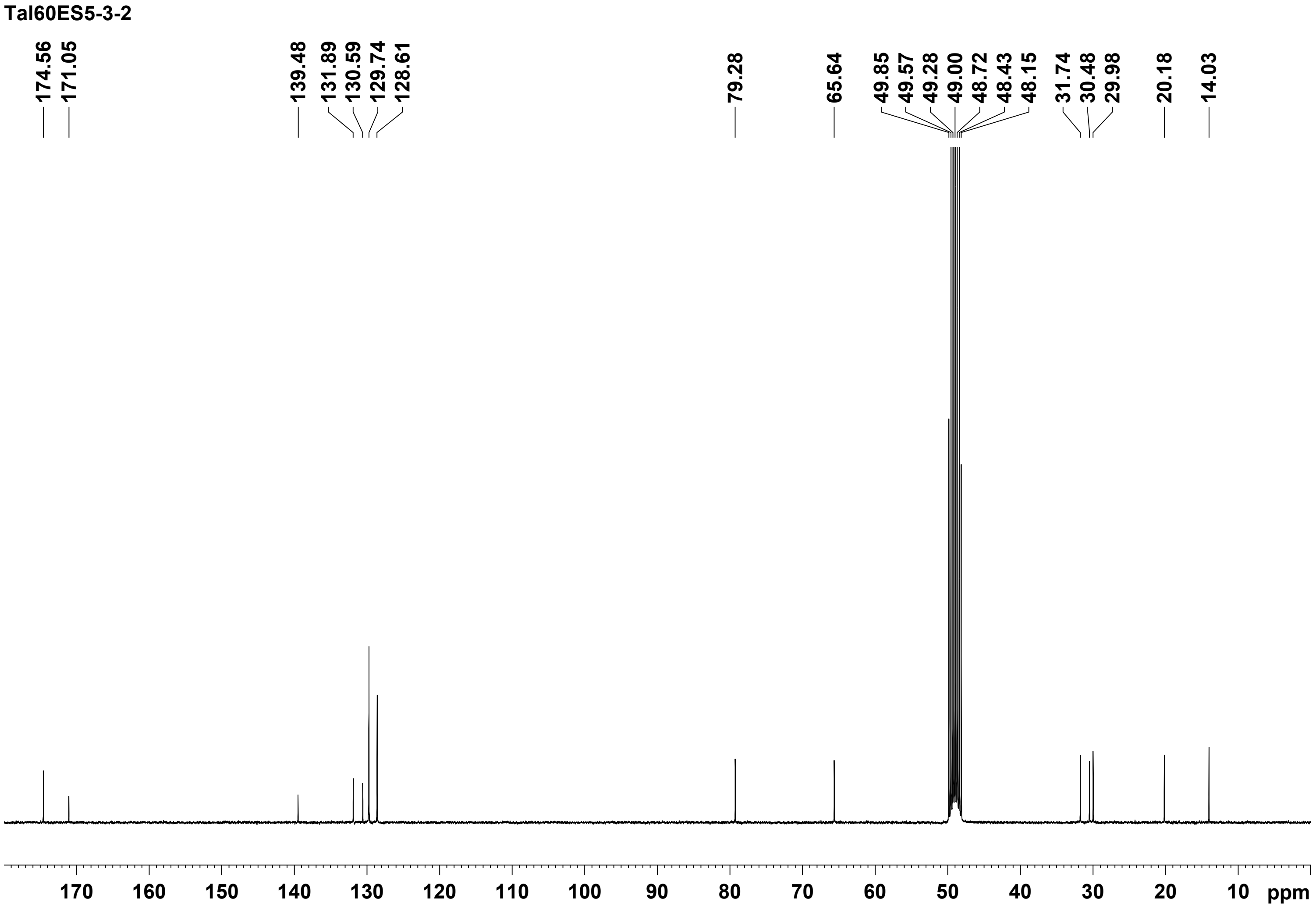

S3. ^{13}C NMR (75 MHz, CD_3OD) spectrum of compound **1**.

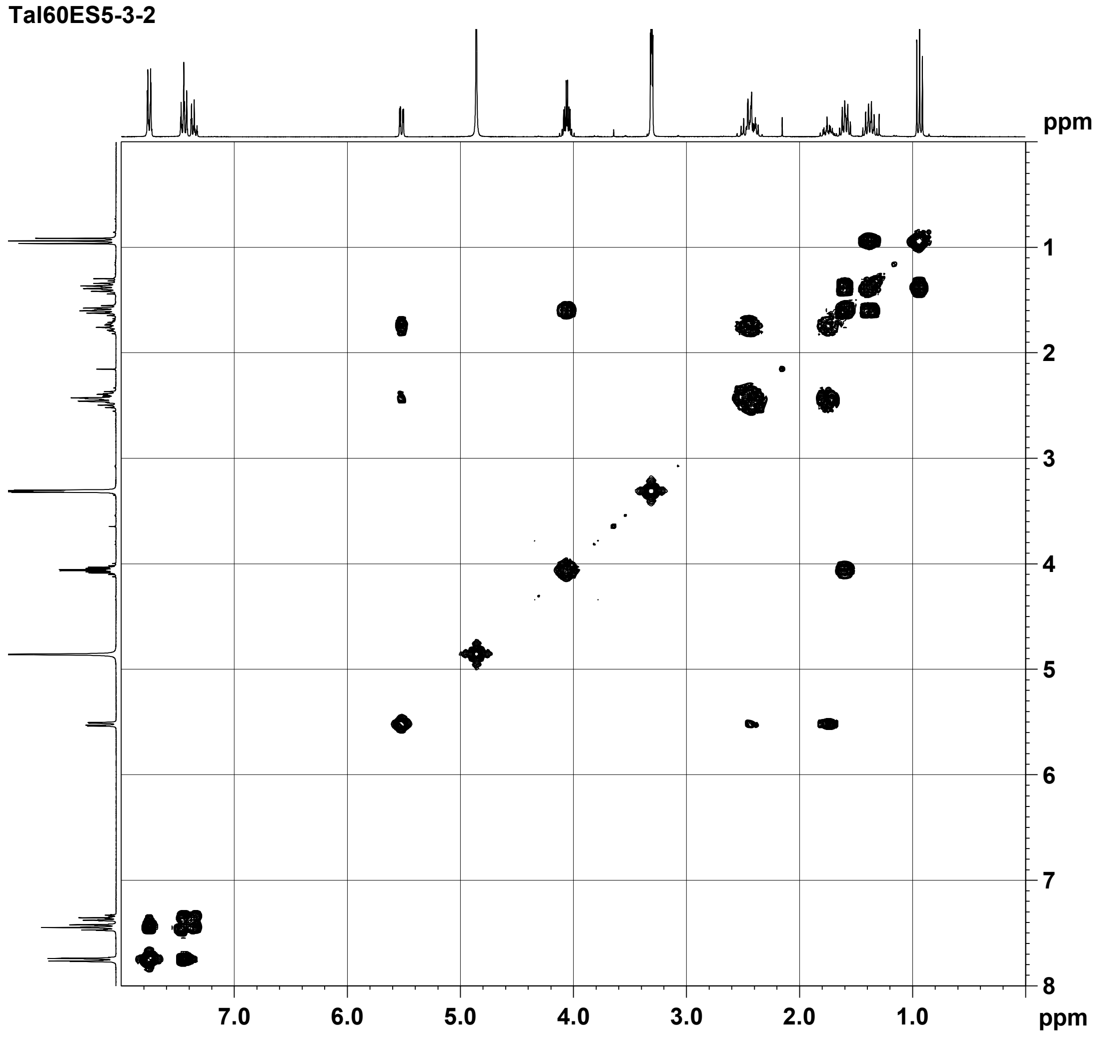

S4. ^{1}H-^{1}H COSY (300 MHz, CD_3OD) spectrum of compound **1**.

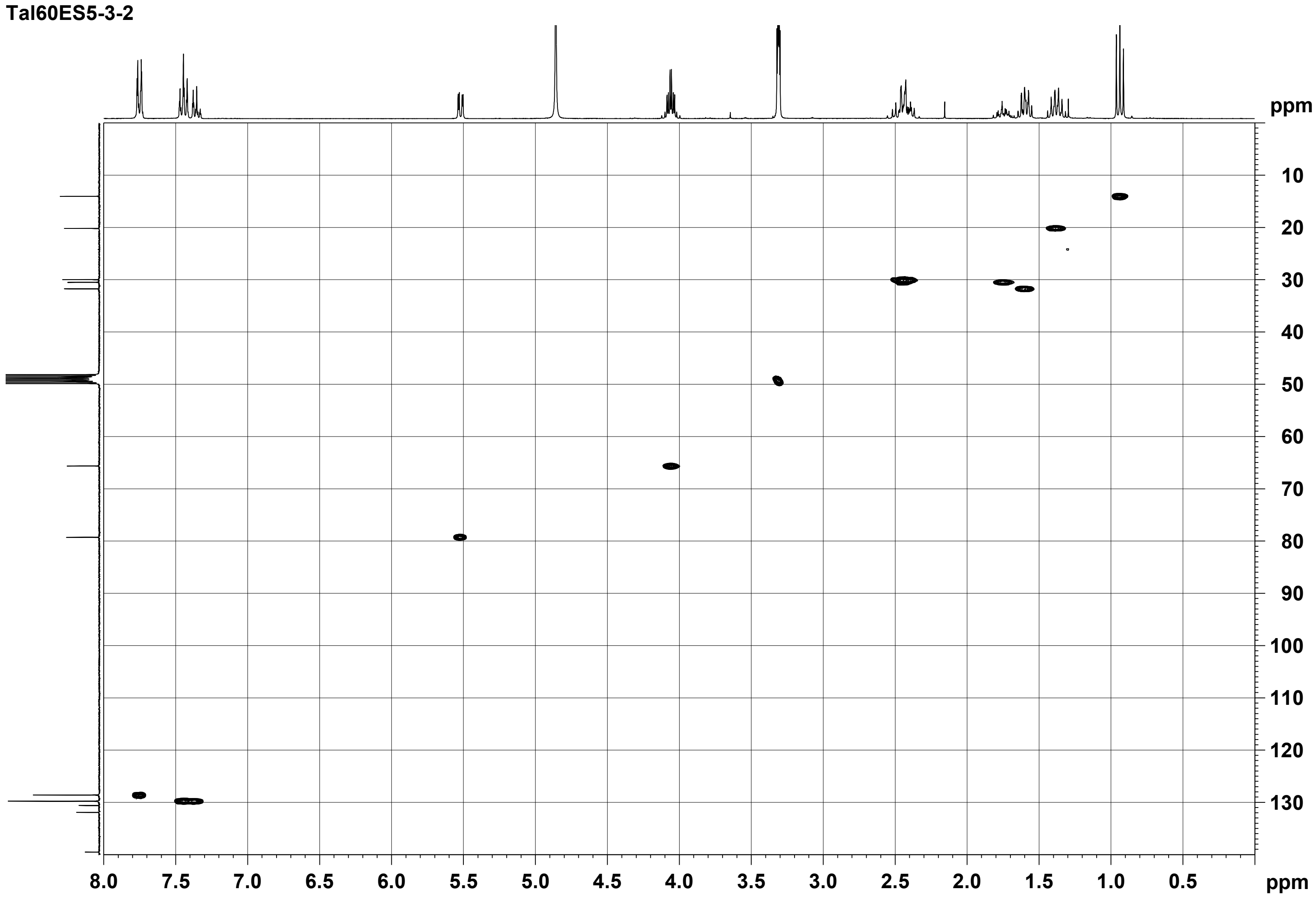

S5. HSQC (300 and 75 MHz, CD_3OD) spectrum of compound **1**.

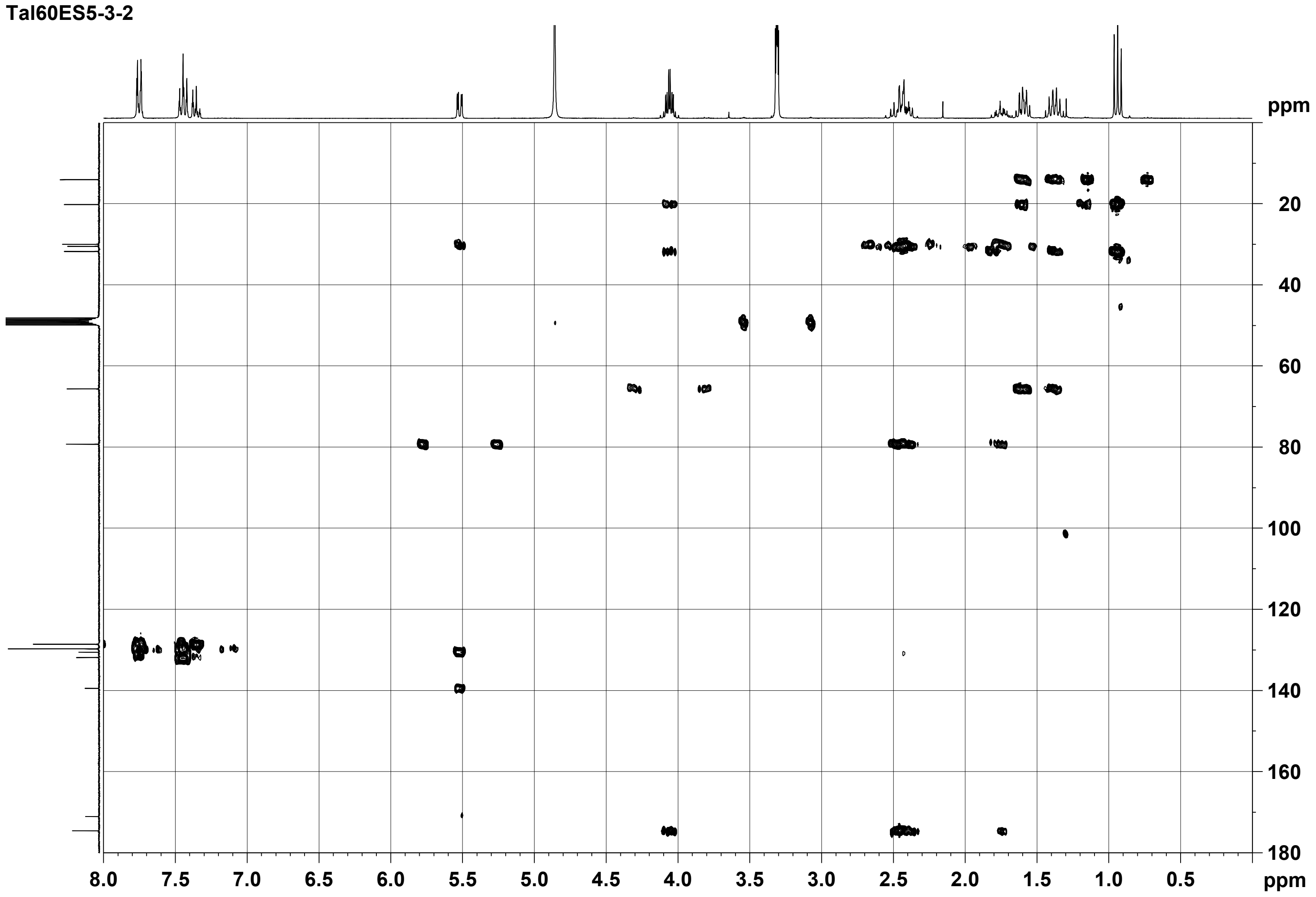

S6. HMBC (300 and 75 MHz, CD_3OD) spectrum of compound **1**.

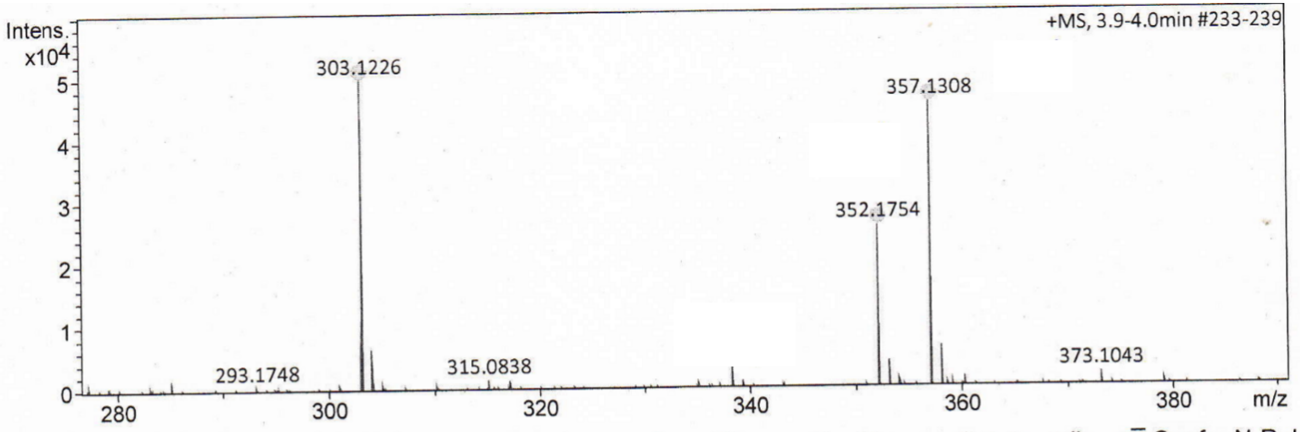

Meas. m/z	#	Ion Formula	m/z	err [ppm]	mSigma	# mSigma	Score	rdb	e⁻ Conf	N-Rule
303.1226	1	C17H19O5	303.1227	0.4	33.6	1	100.00	8.5	even	ok
352.1754	1	C18H26NO6	352.1755	0.2	27.3	1	100.00	6.5	even	ok
357.1308	1	C18H22NaO6	357.1309	0.3	34.6	1	100.00	7.5	even	ok

S7. HRESIMS spectrum of compound **2**.

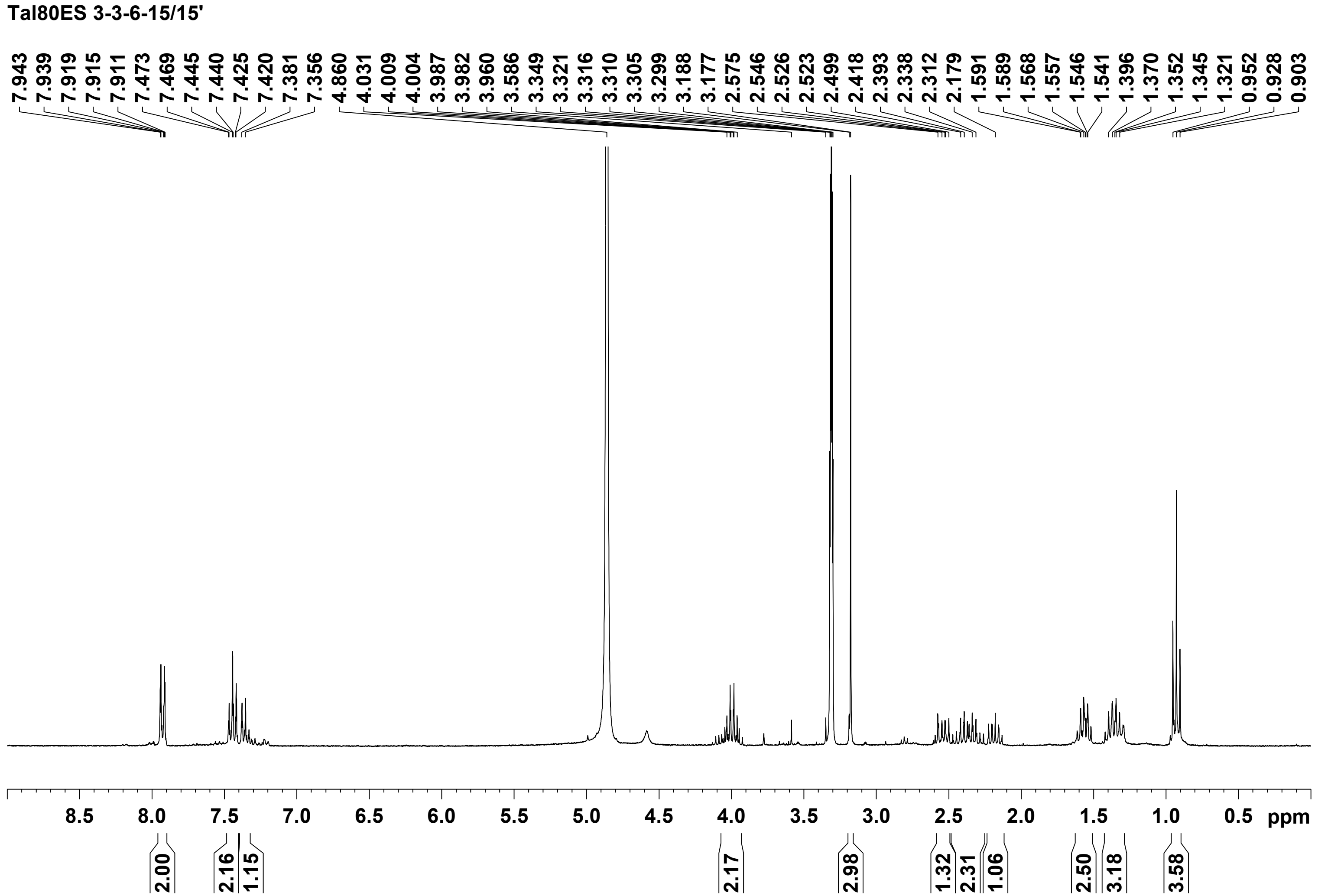

S8. ^{1}H NMR (300 MHz, CD_3OD) spectrum of compound **2**.

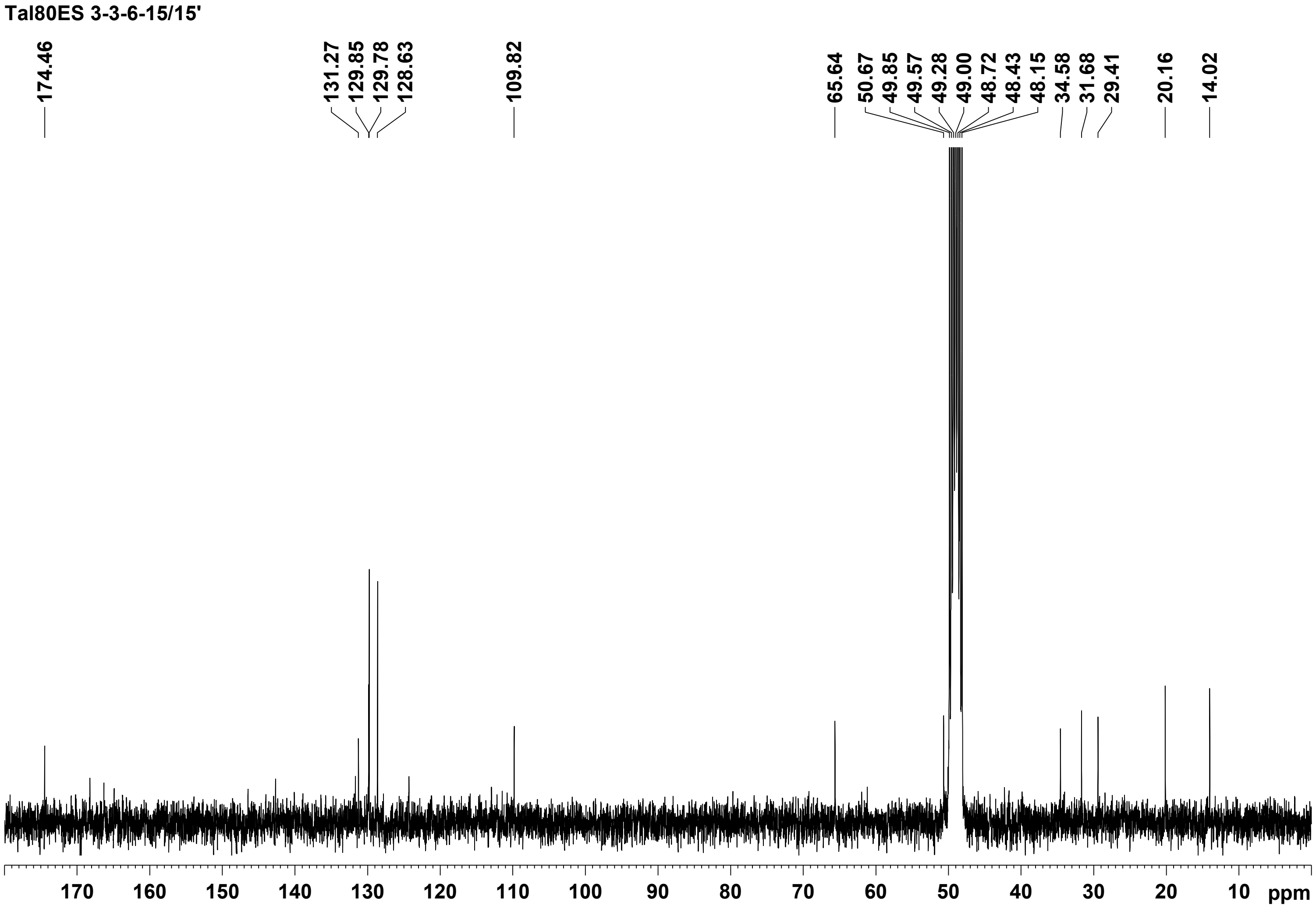

S9. ^{13}C NMR (75 MHz, CD_3OD) spectrum of compound **2**.

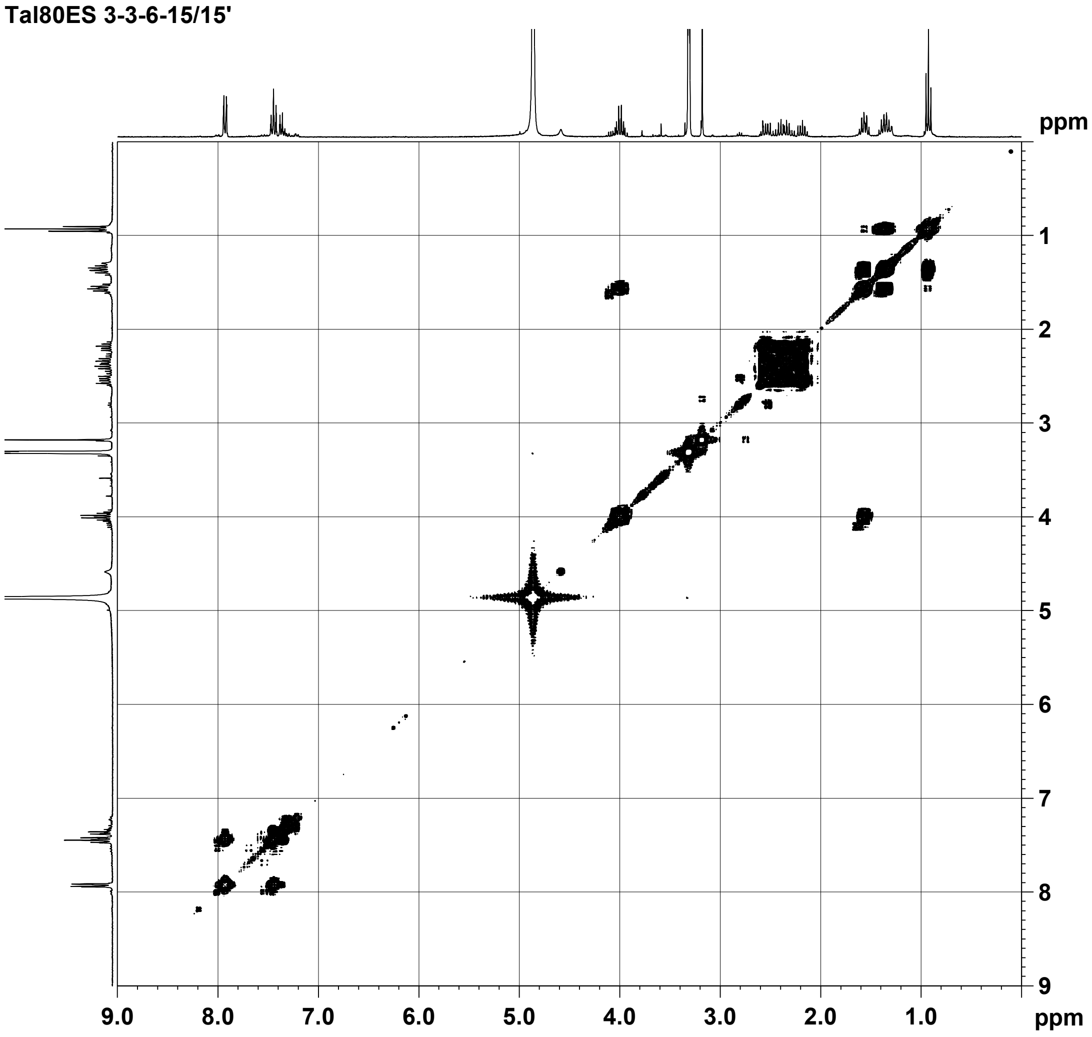

S10. ^{1}H-^{1}H COSY (300 MHz, CD_3OD) spectrum of compound **2**.

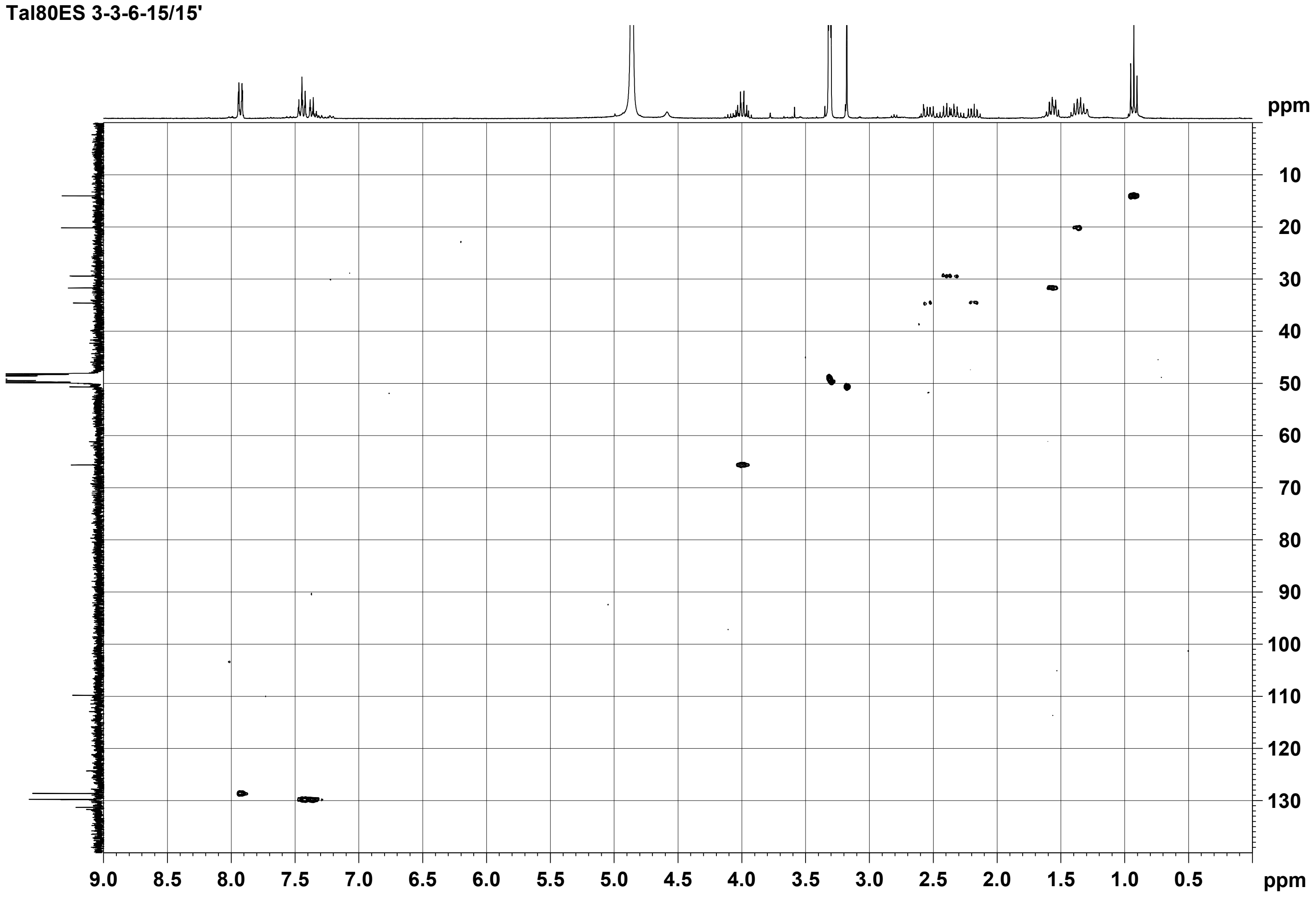

S11. HSQC (300 and 75 MHz, CD_3OD) spectrum of compound **2**.

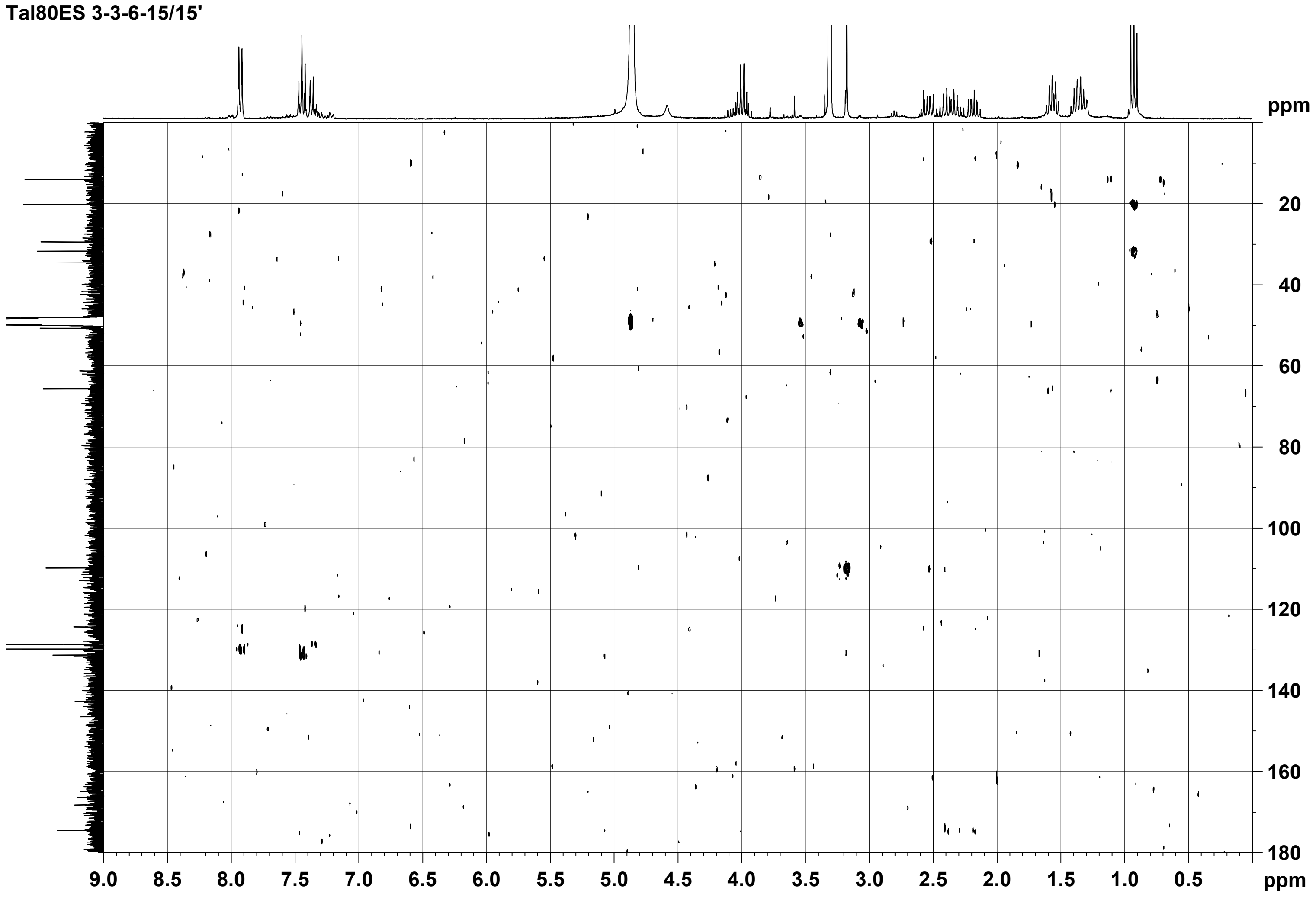

S12. HMBC (300 and 75 MHz, CD_3OD) spectrum of compound **2**.

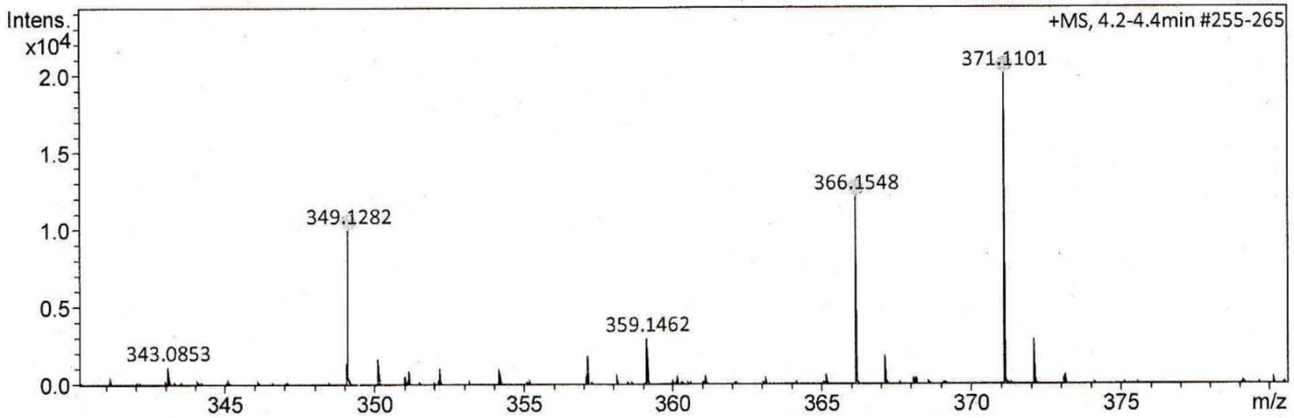

Meas. m/z	#	Ion Formula	m/z	err [ppm]	mSigma	# mSigma	Score	rdb	e⁻ Conf	N-Rule
349.1282	1	C18H21O7	349.1282	-0.0	38.1	1	100.00	8.5	even	ok
366.1548	1	C18H24NO7	366.1547	-0.2	29.9	1	100.00	7.5	even	ok
371.1101	1	C18H20NaO7	371.1101	-0.1	33.0	1	100.00	8.5	even	ok

S13. HRESIMS spectrum of compound **3**.

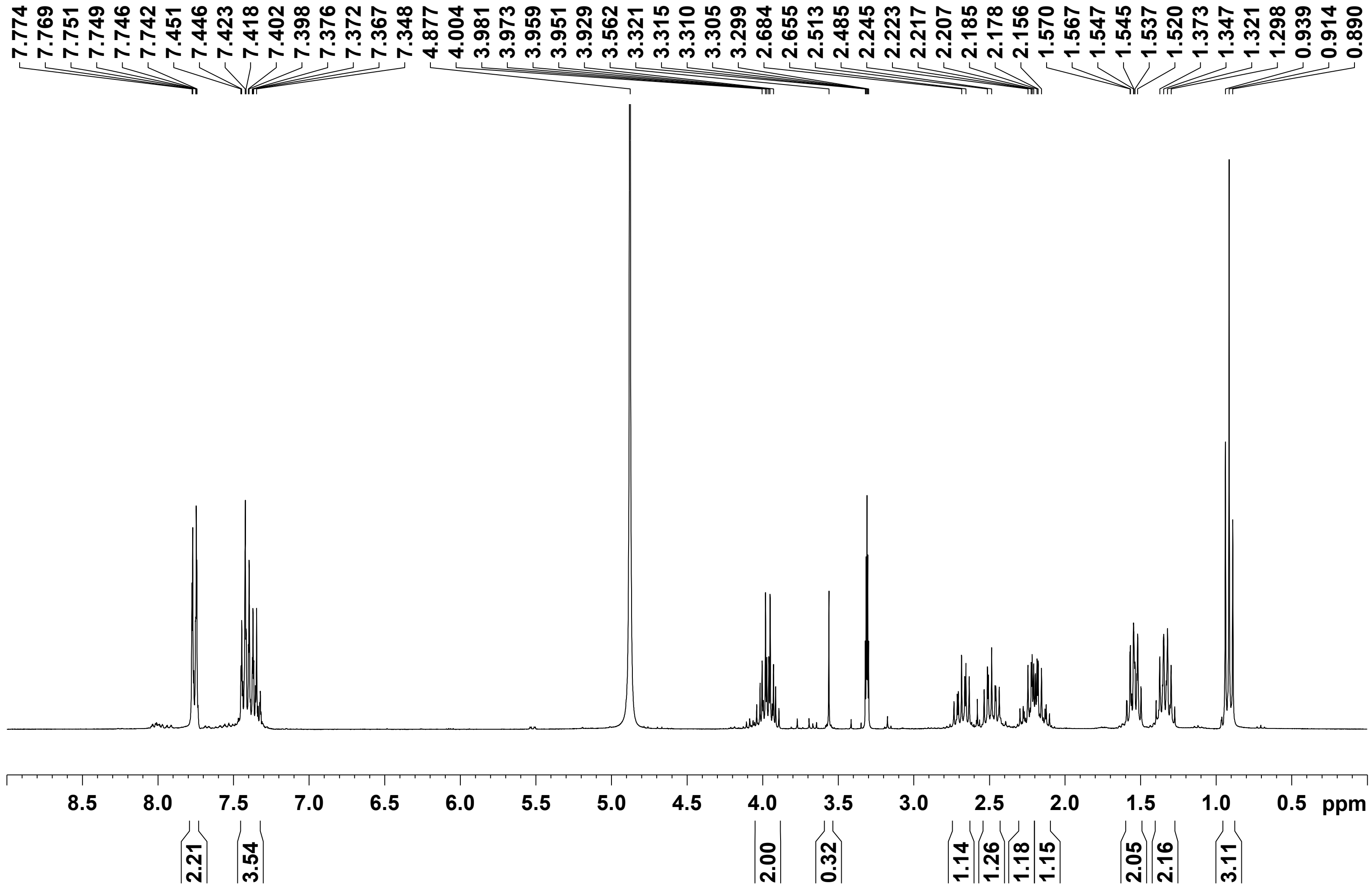

S14. ^{1}H NMR (300 MHz, CD_3OD) spectrum of compound **3**.

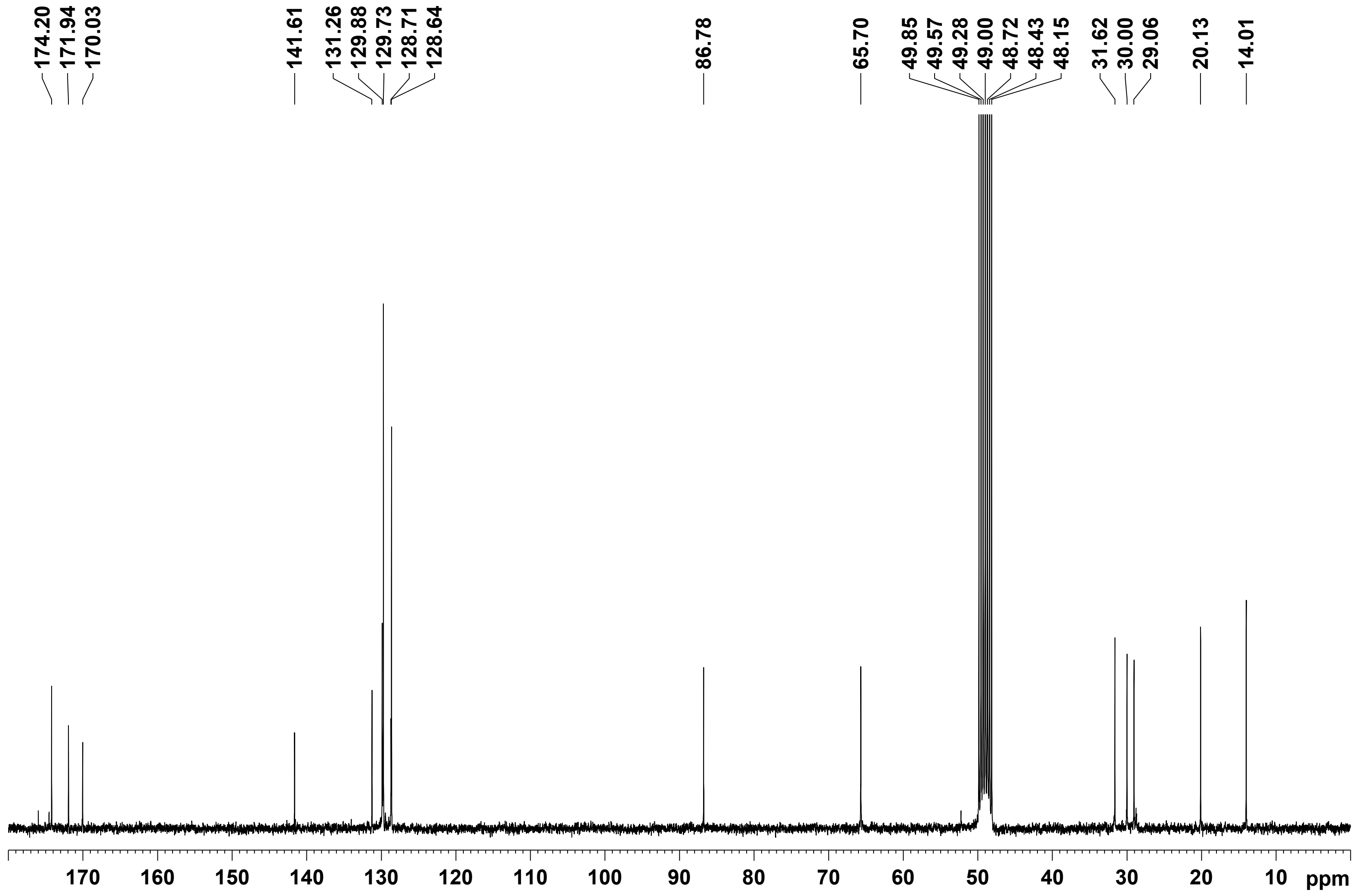

S15. ^{13}C NMR (75 MHz, CD_3OD) spectrum of compound **3**.

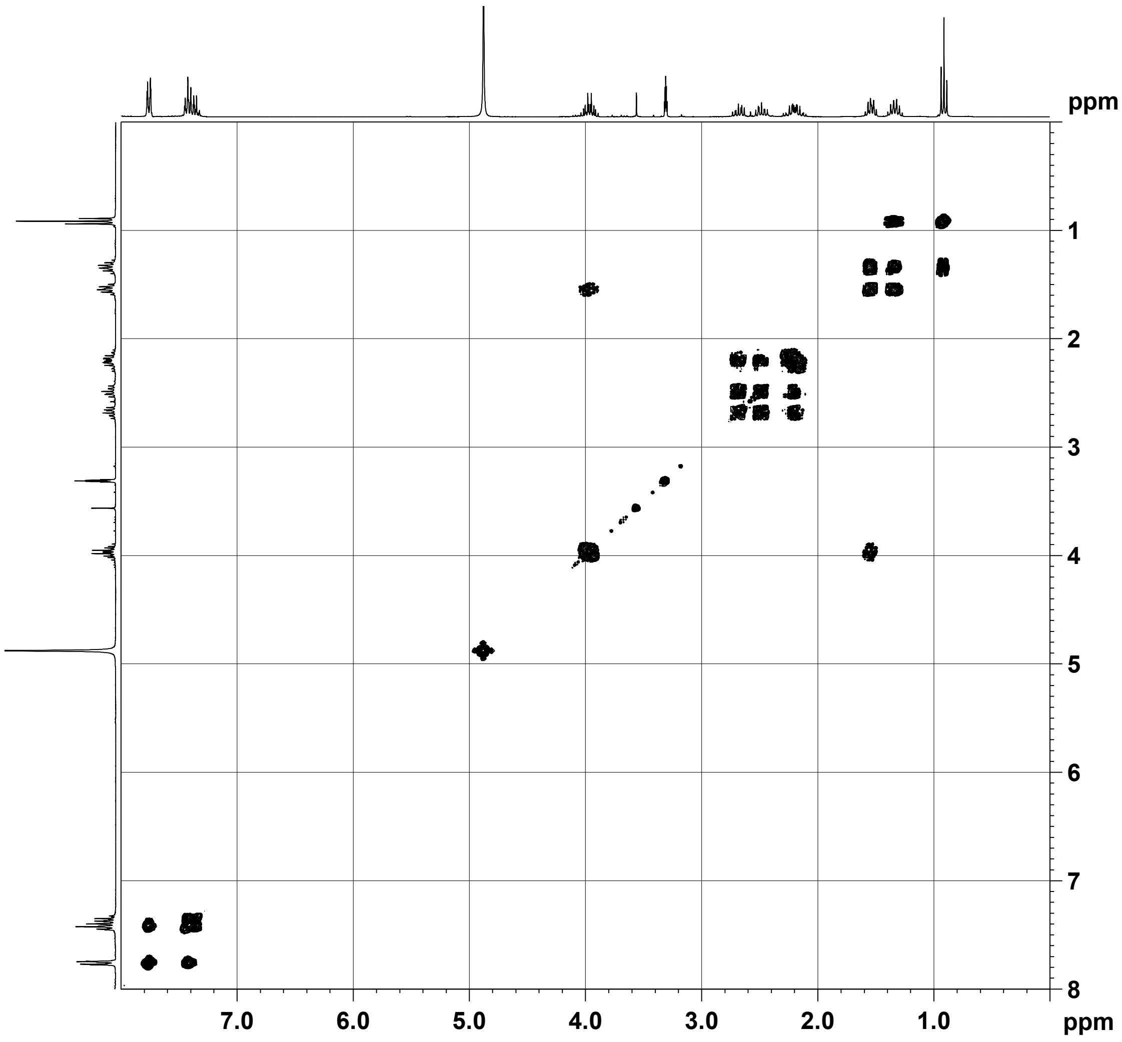

S16. ^{1}H-^{1}H COSY (300 MHz, CD_3OD) spectrum of compound **3**.

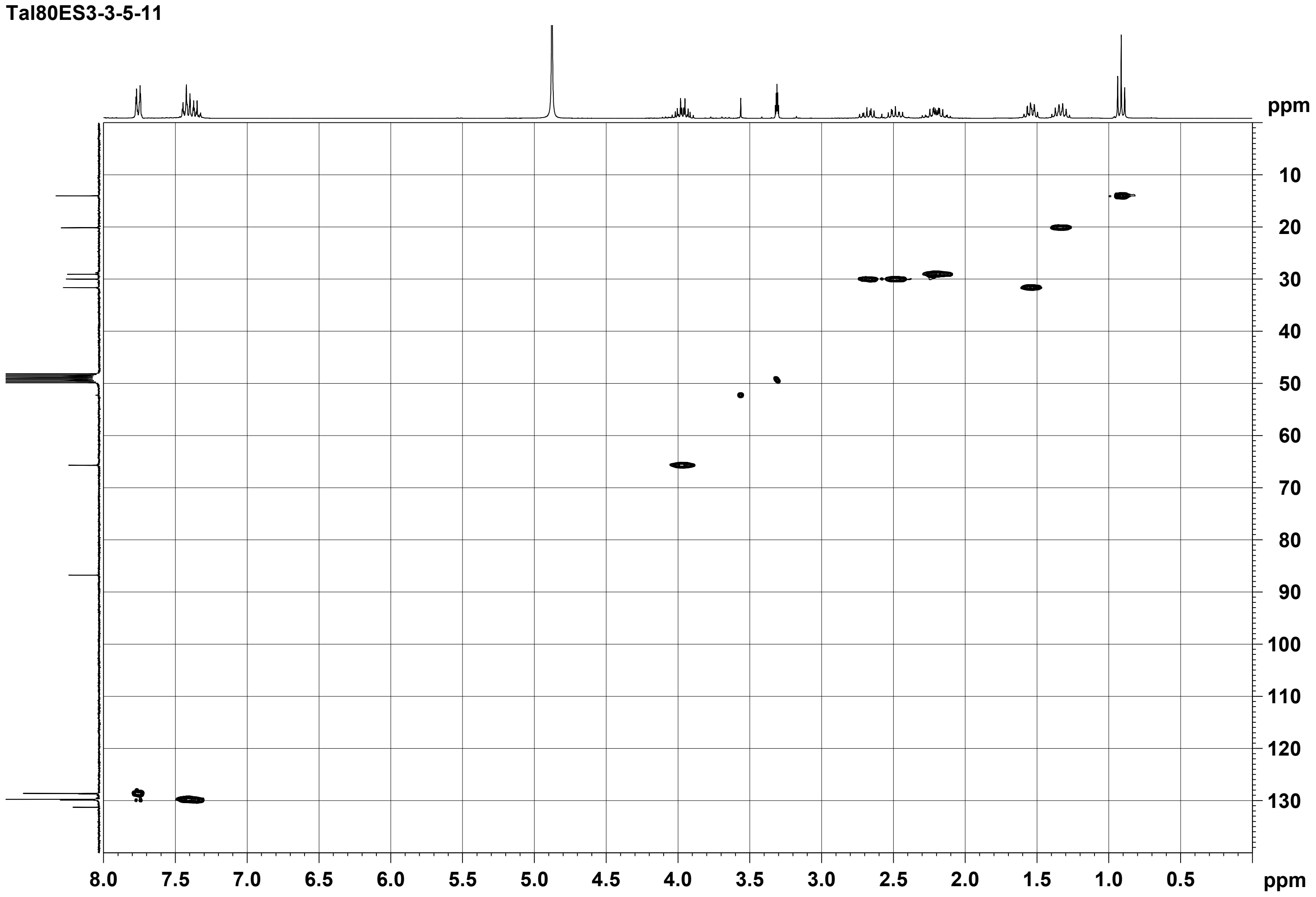

S17. HSQC (300 and 75 MHz, CD_3OD) spectrum of compound **3**.

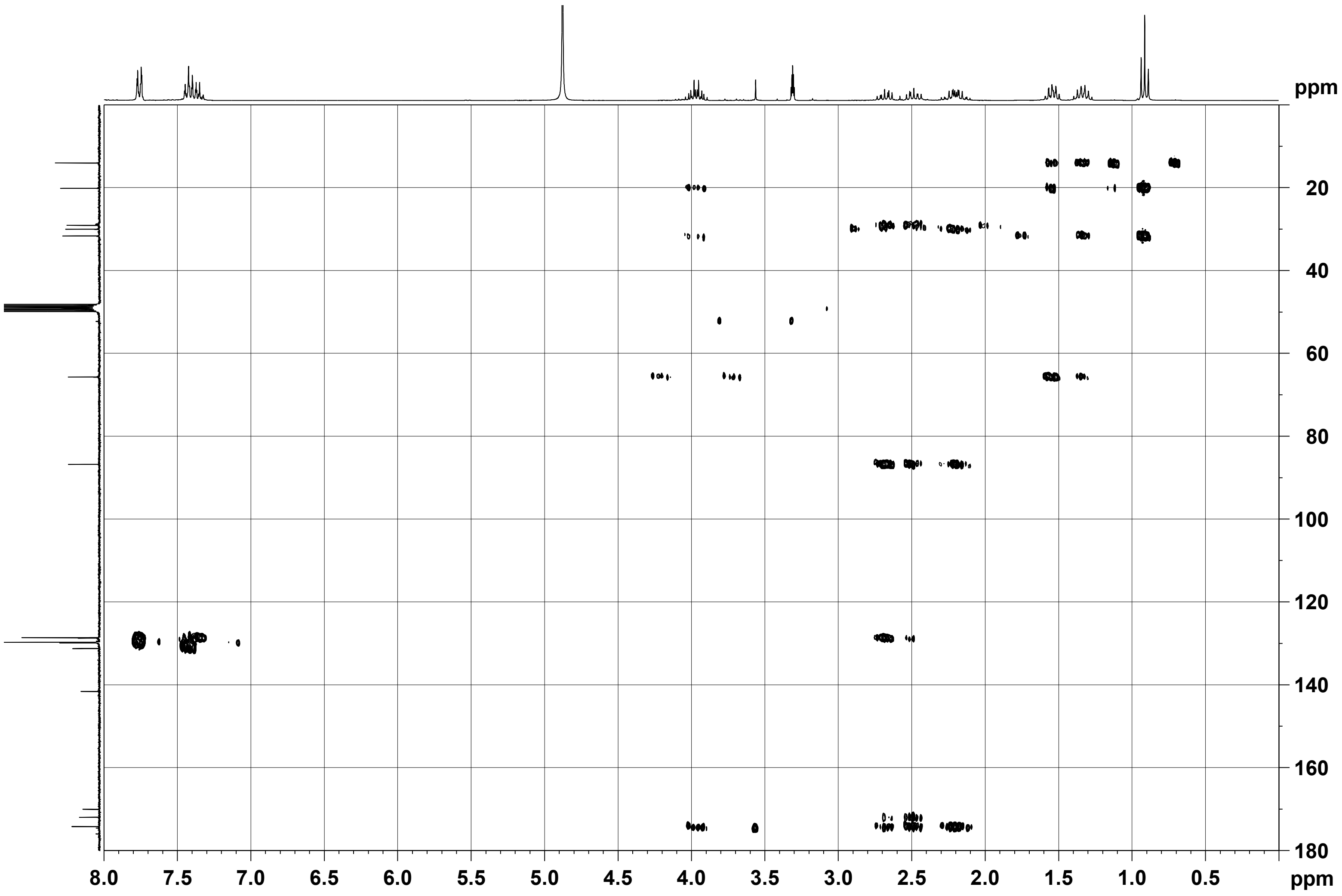

S18. HMBC (300 and 75 MHz, CD_3OD) spectrum of compound **3**.

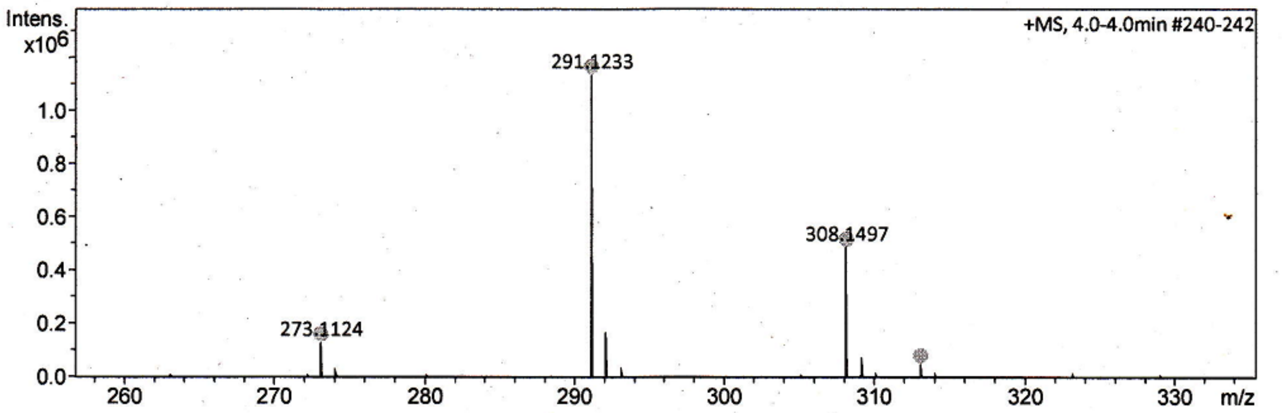

Meas. m/z	#	Ion Formula	m/z	err [ppm]	mSigma	# mSigma	Score	rdb	e⁻ Conf	N-Rule
273.1124	1	C16H17O4	273.1121	-0.8	9.4	1	100.00	8.5	even	ok
291.1233	1	C16H19O5	291.1227	-1.9	15.8	1	100.00	7.5	even	ok

S19. HRESIMS spectrum of compound **6**.

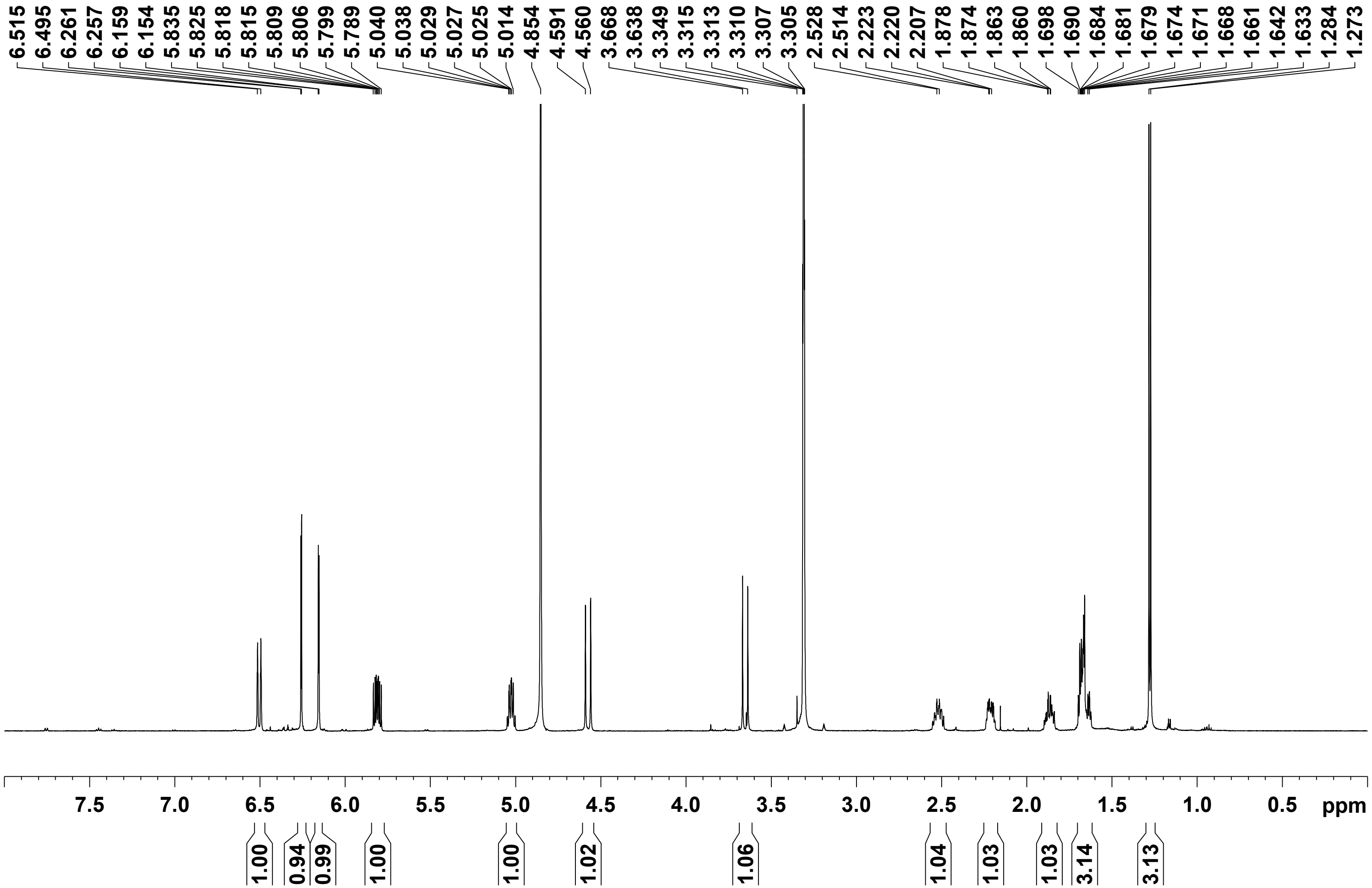

S20. ^{1}H NMR (600 MHz, CD_3OD) spectrum of compound **6**.

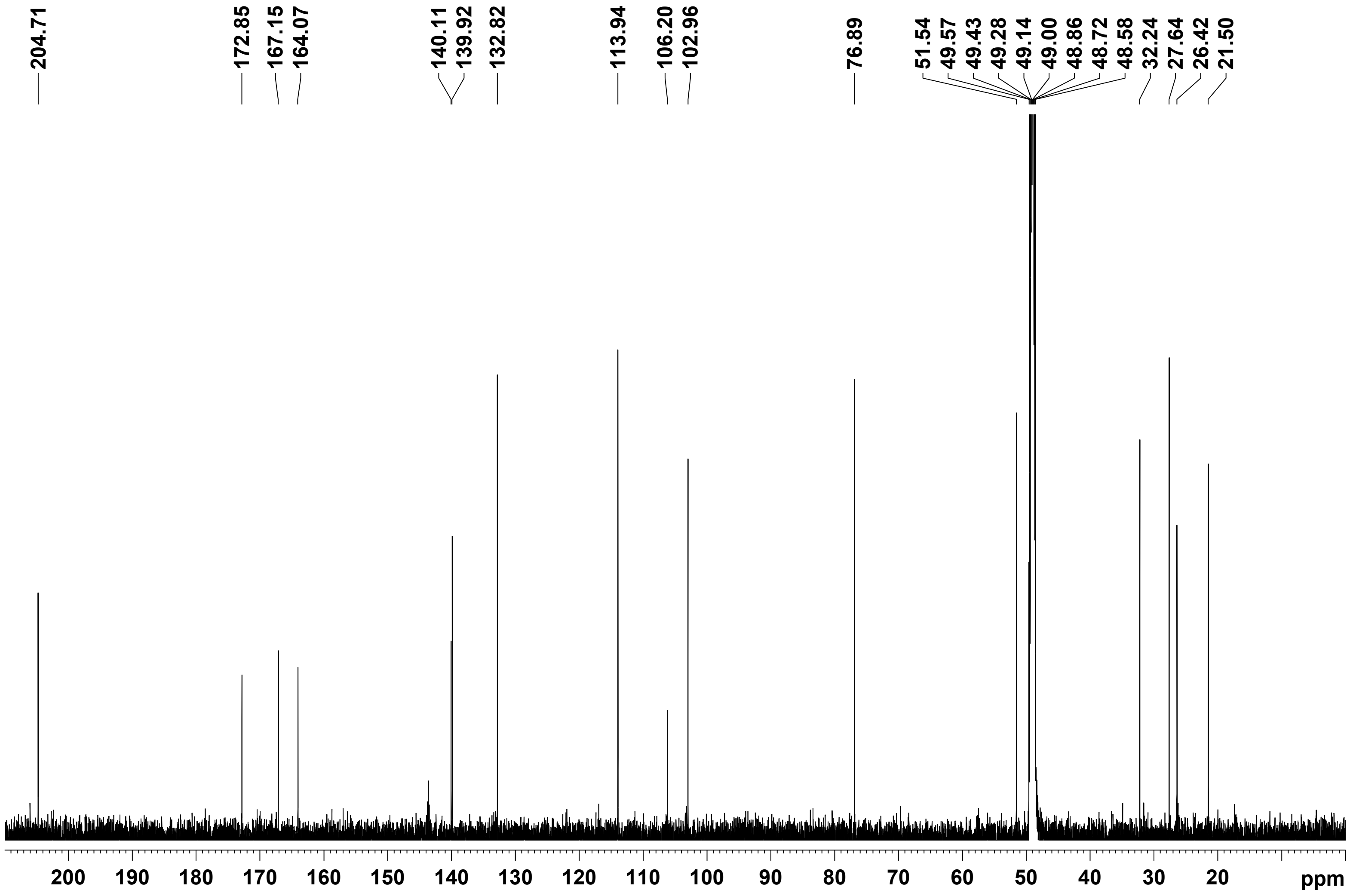

S21. ^{13}C NMR (150 MHz, CD_3OD) spectrum of compound **6**.

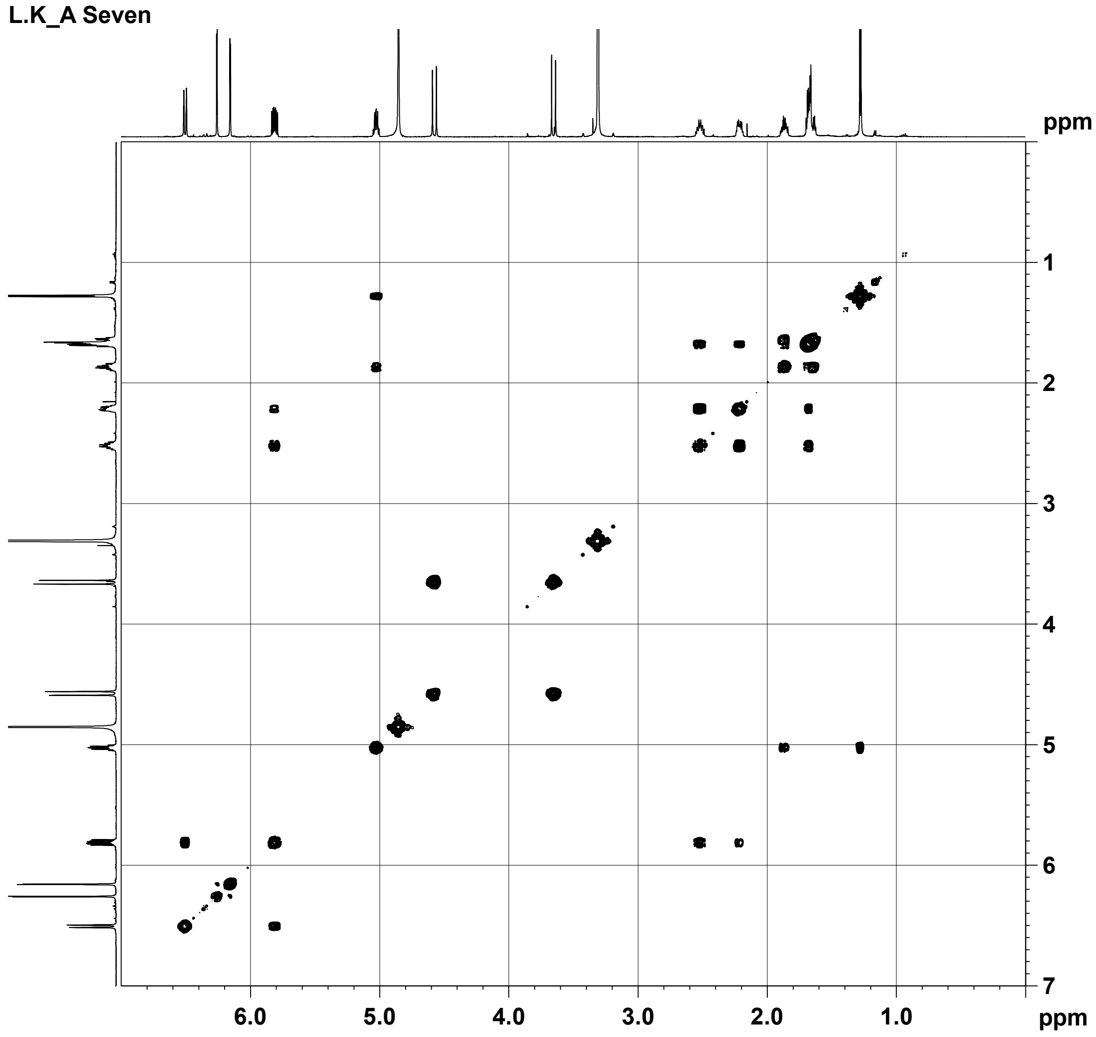

S22. ^{1}H-^{1}H COSY (600 MHz, CD_3OD) spectrum of compound **6**.

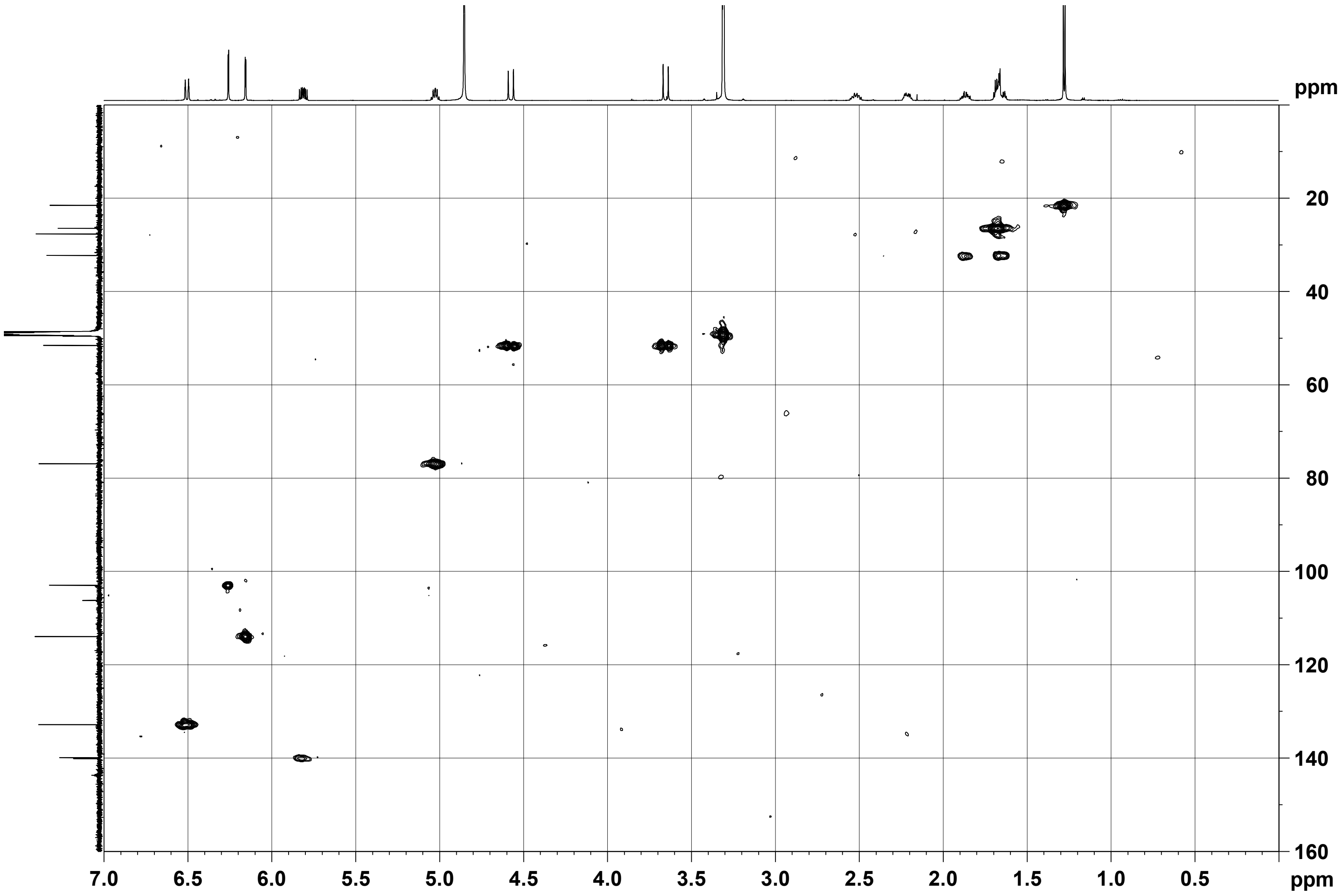

S23. HSQC (600 and 150 MHz, CD_3OD) spectrum of compound **6**.

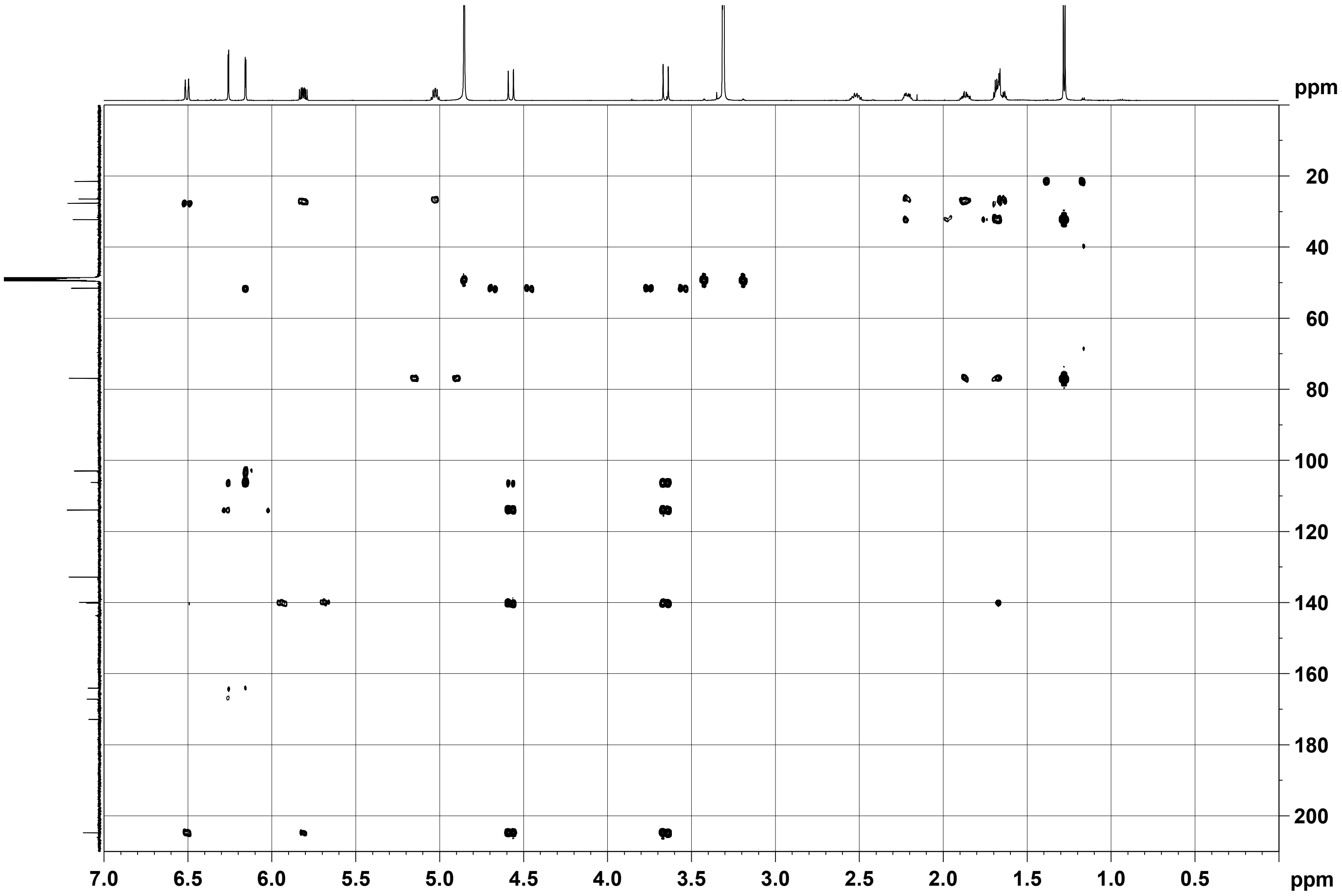

S24. HMBC (600 and 150 MHz, CD_3OD) spectrum of compound **6**.

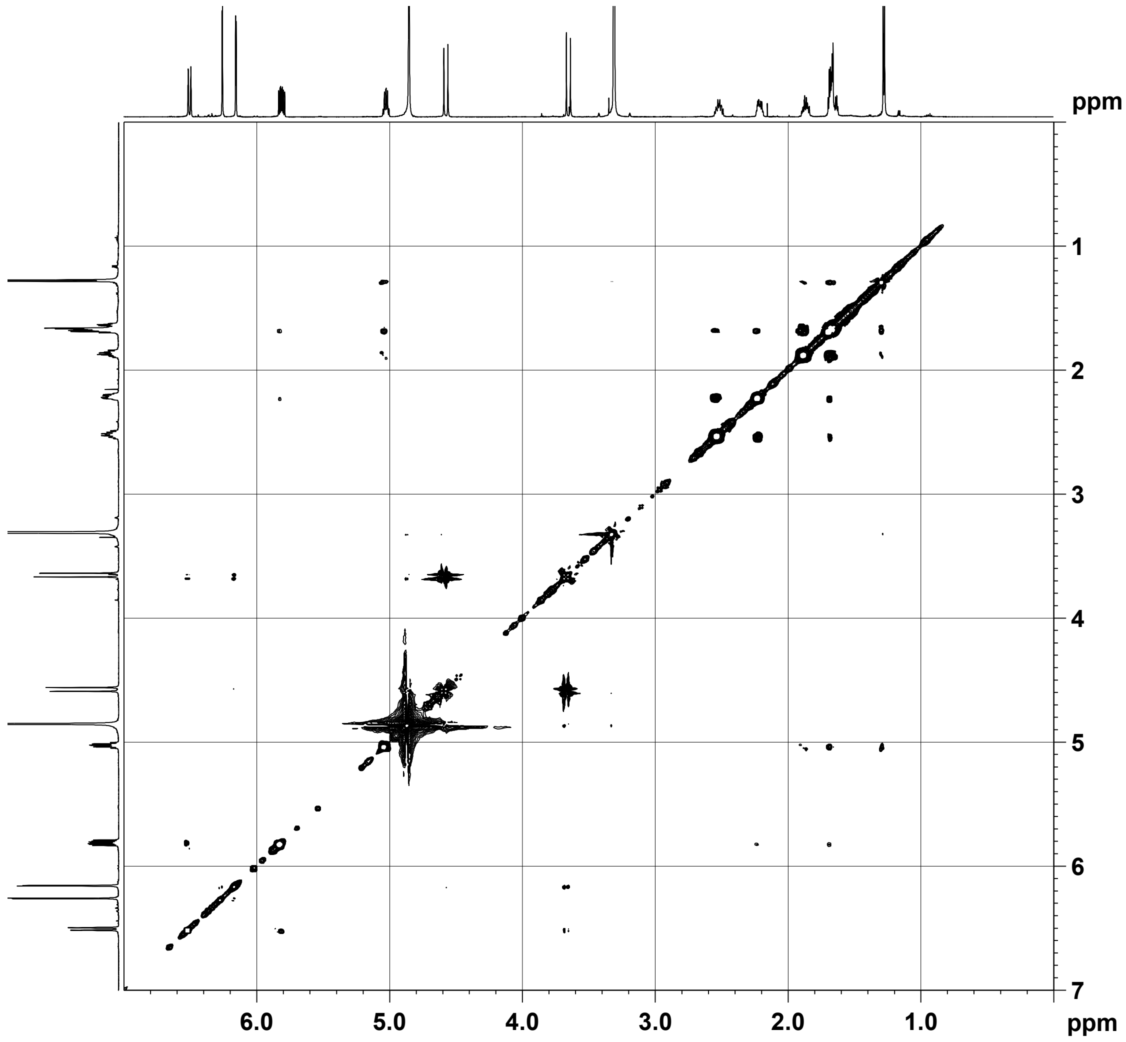

S25. ROESY (600 MHz, CD_3OD) spectrum of compound **6**.

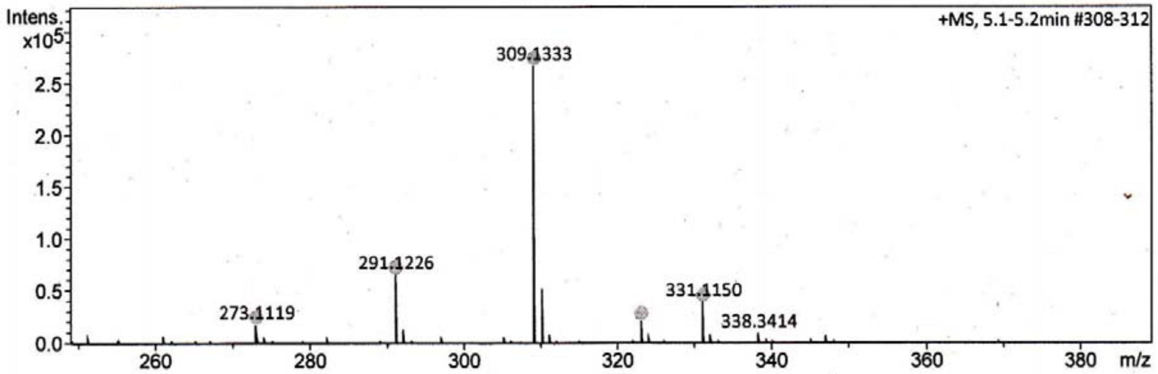

Meas. m/z	#	Ion Formula	m/z	err [ppm]	mSigma	# mSigma	Score	rdb	e¯ Conf	N-Rule
273.1119	1	C16H17O4	273.1121	1.0	5.5	1	100.00	8.5	even	ok
291.1226	1	C16H19O5	291.1227	0.3	14.9	1	100.00	7.5	even	ok
309.1333	1	C16H21O6	309.1333	-0.1	10.6	1	100.00	6.5	even	ok

S26. HRESIMS spectrum of compound **7**.

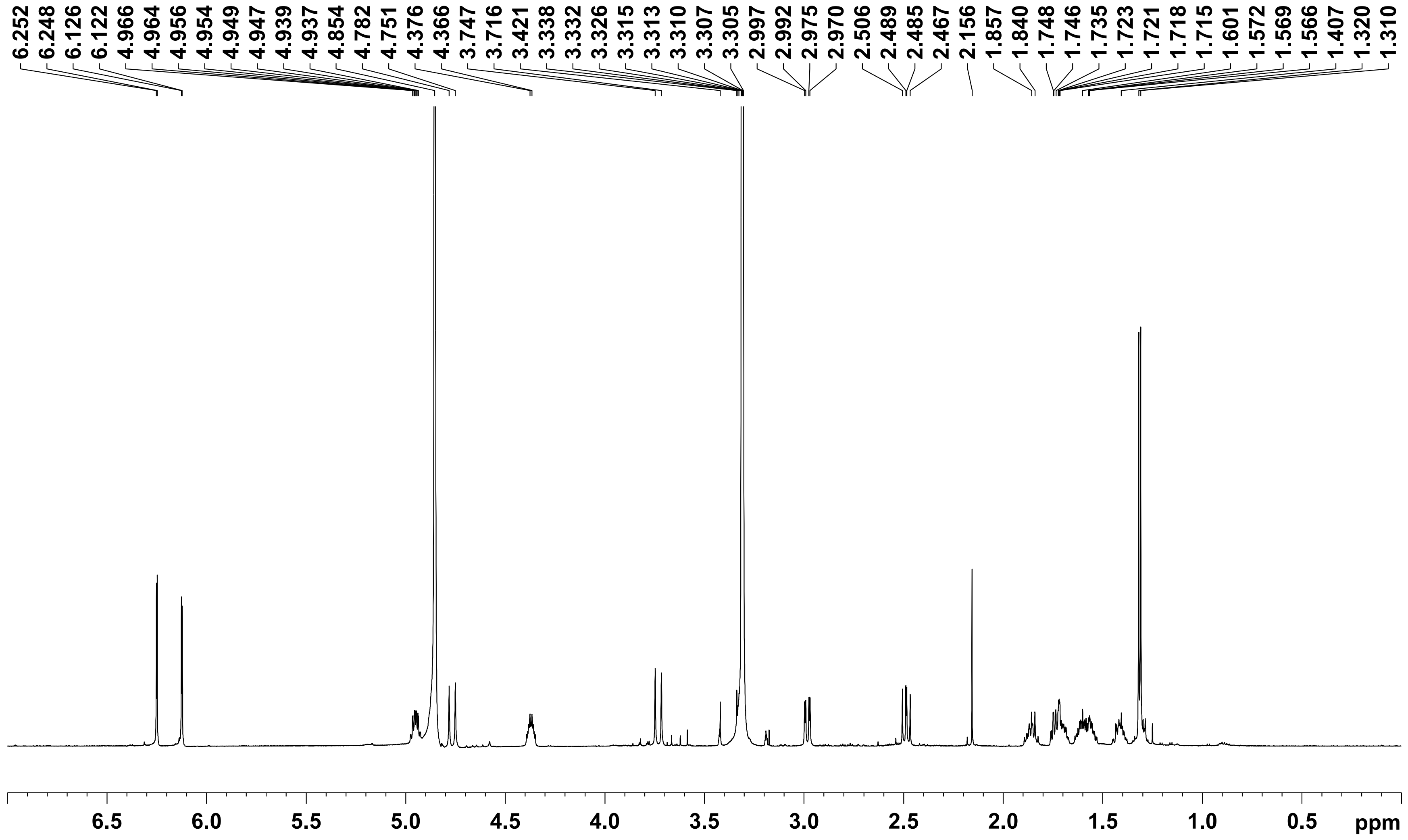

S27. ^{1}H NMR (600 MHz, CD_3OD) spectrum of compound **7**.

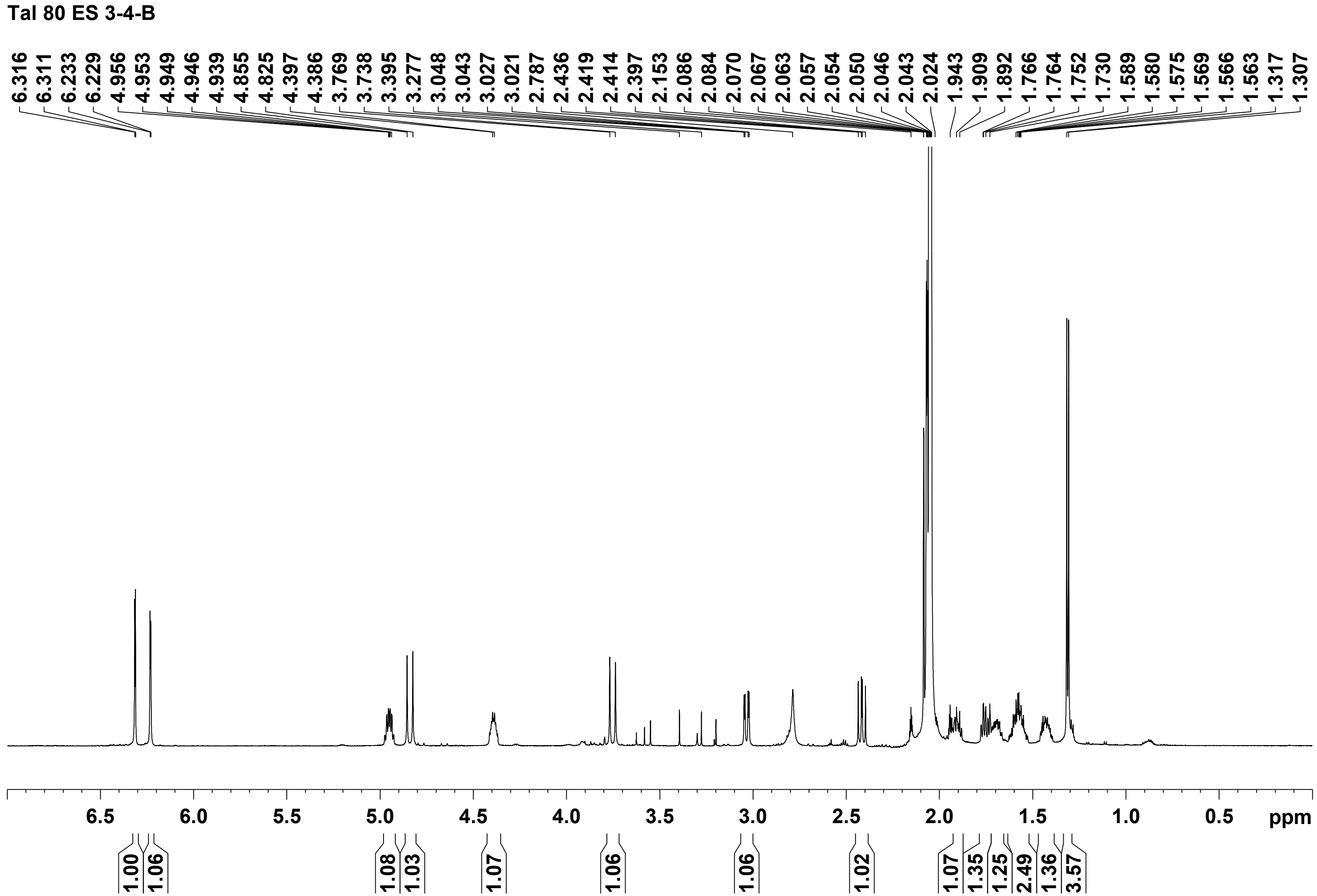

S28. ^{1}H NMR (600 MHz, CD_3COCD_3) spectrum of compound **7**.

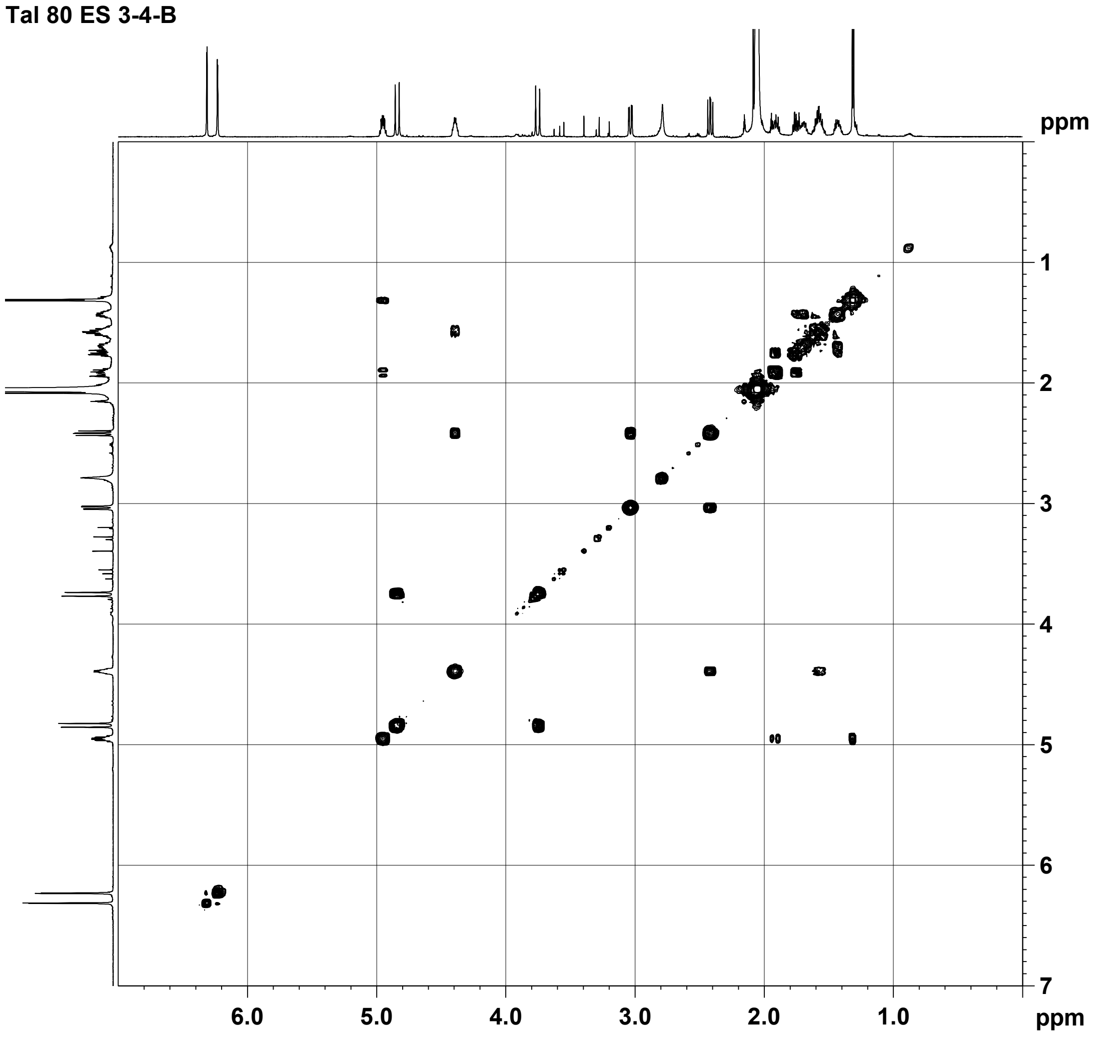

S29. ^{1}H-^{1}H COSY (600 MHz, CD_3COCD_3) spectrum of compound **7**.

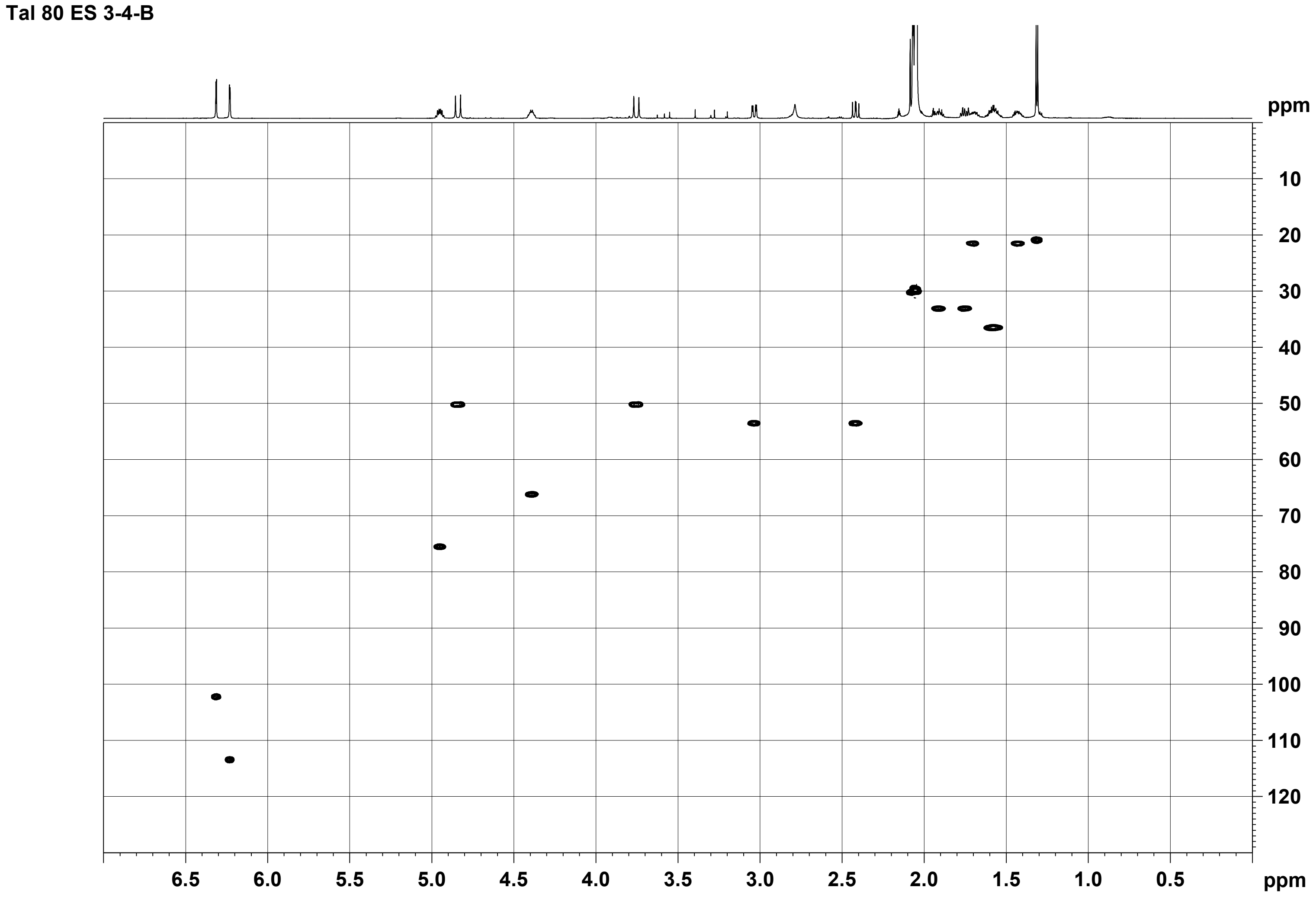

S30. HSQC (600 and 150 MHz, CD_3COCD_3) spectrum of compound **7**.

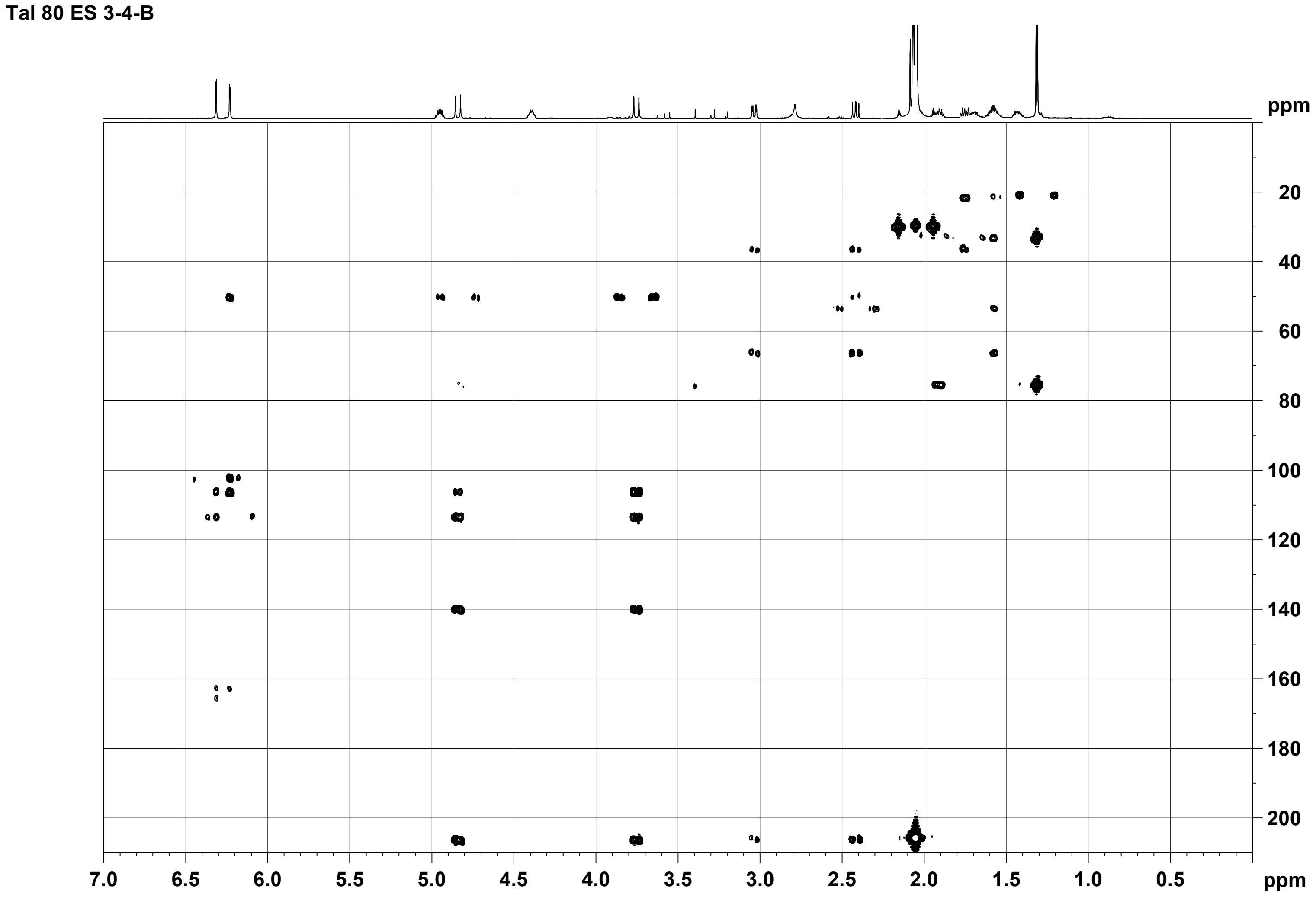

S31. HMBC (600 and 150 MHz, CD_3COCD_3) spectrum of compound **7**.

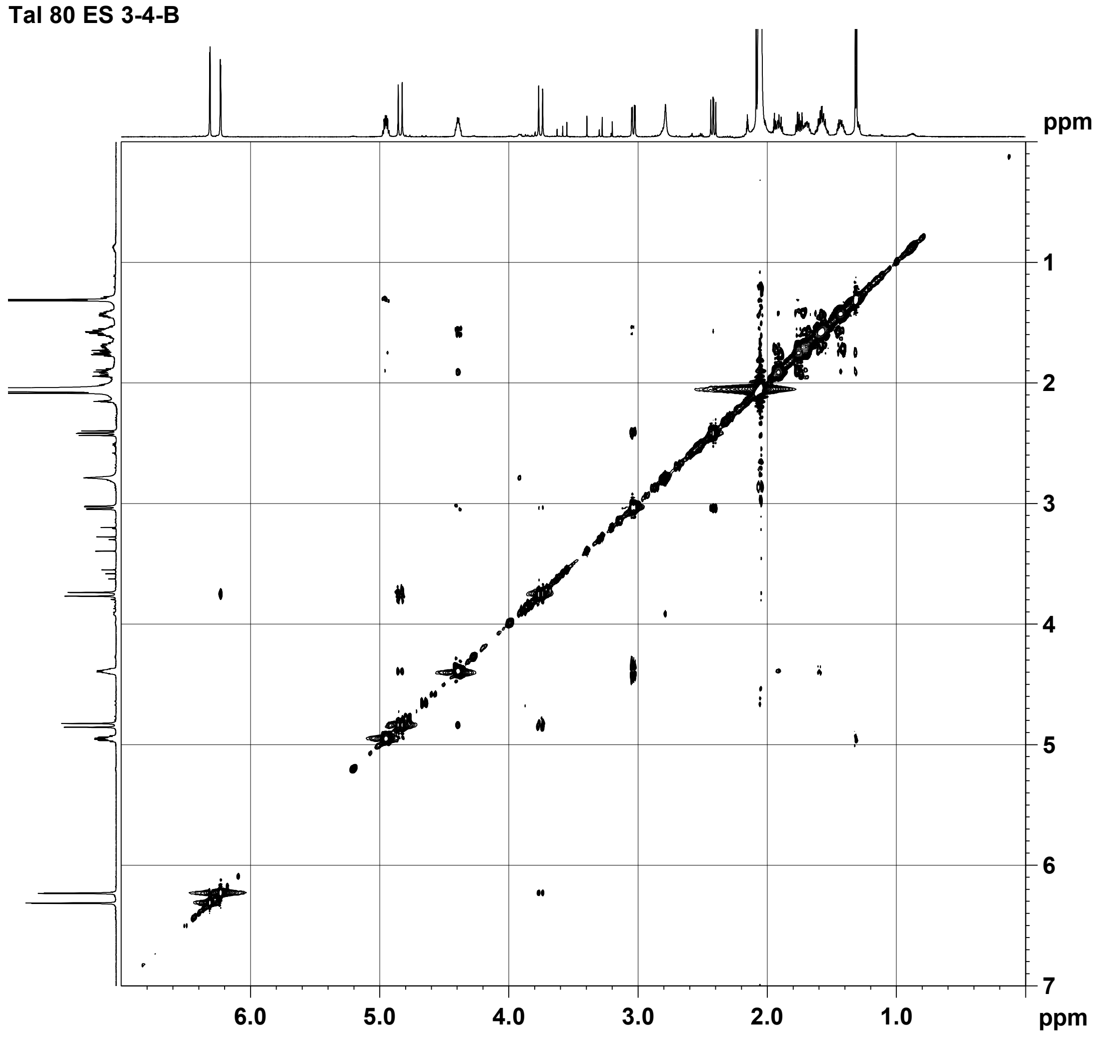

S32. ROESY (600 MHz, CD_3COCD_3) spectrum of compound **7**.

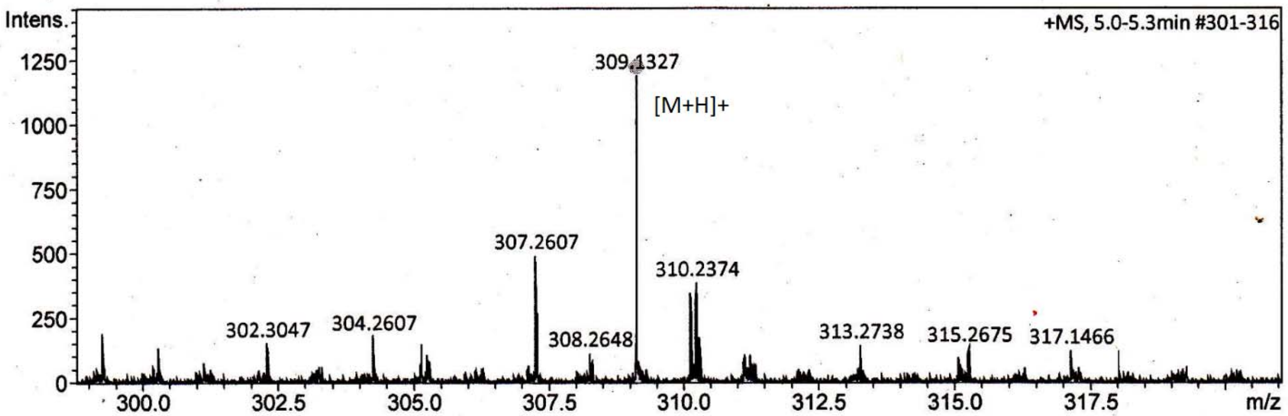

Meas. m/z	#	Ion Formula	m/z	err [ppm]	mSigma	# mSigma	Score	rdb	e¯ Conf	N-Rule
309.1327	1	C16H21O6	309.1333	1.8	77.1	1	100.00	6.5	even	ok

S33. HRESIMS spectrum of compound **8**.

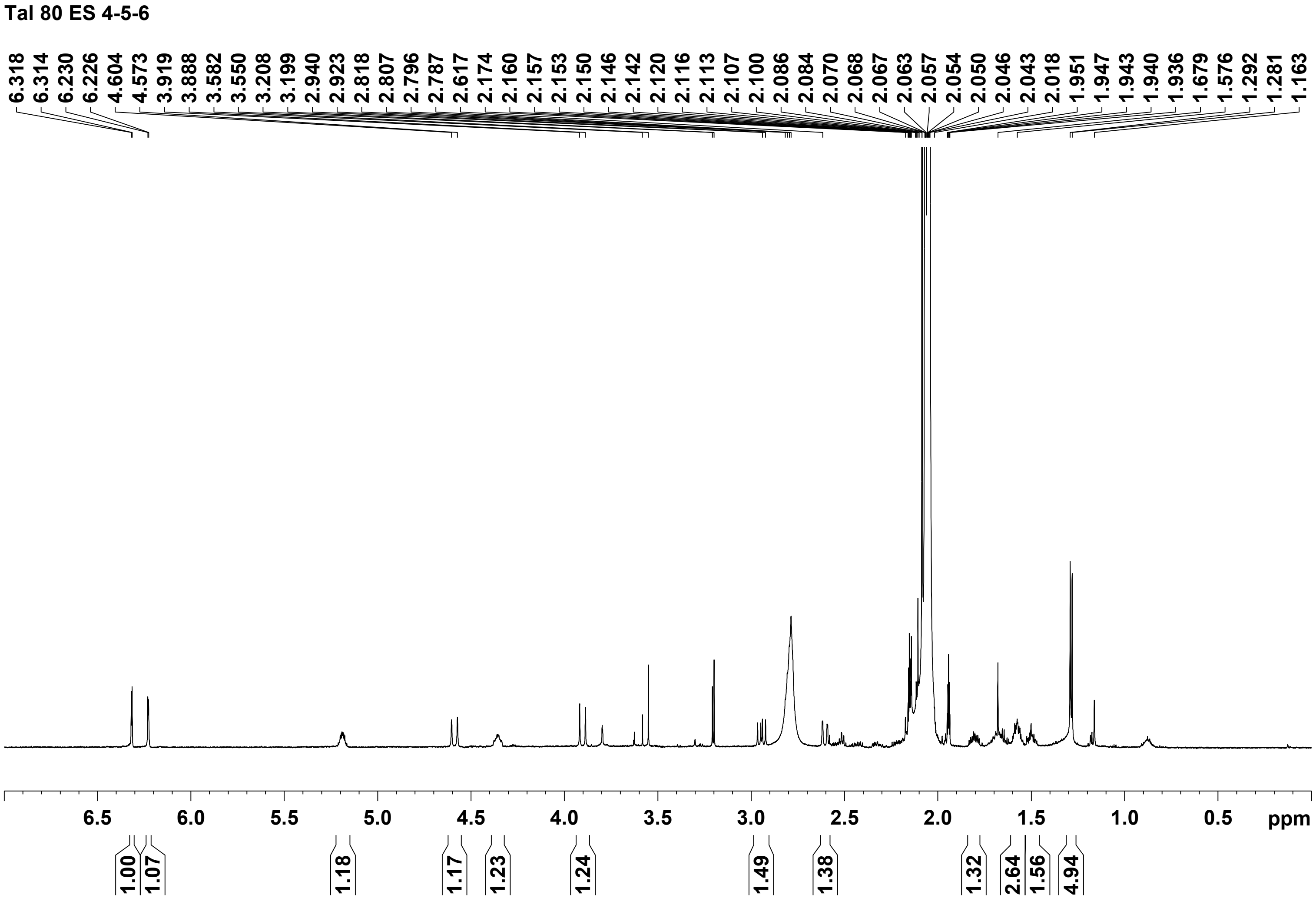

S34. ^{1}H NMR (600 MHz, CD_3COCD_3) spectrum of compound **8**.

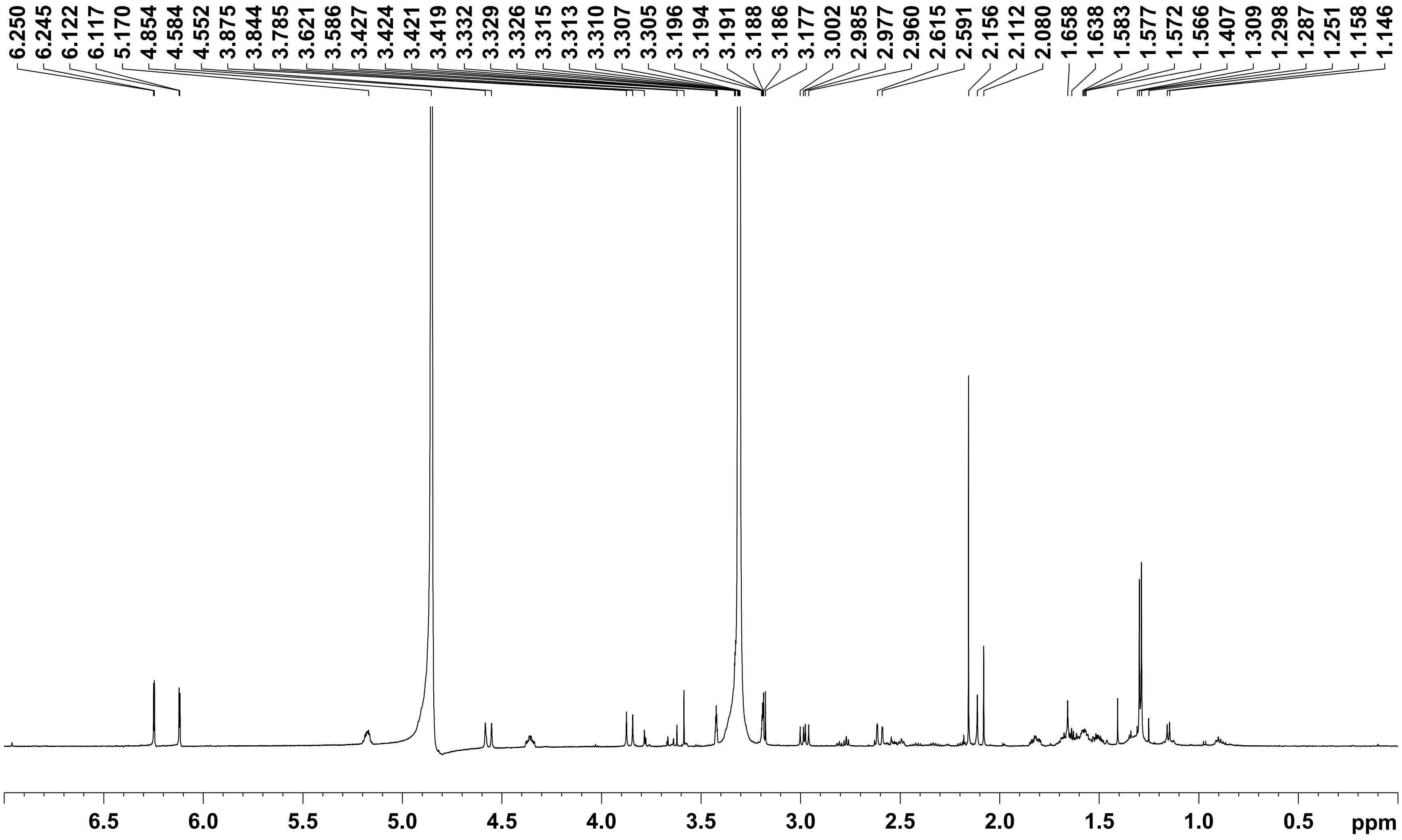

S35. ^{1}H NMR (600 MHz, CD_3OD) spectrum of compound **8**.

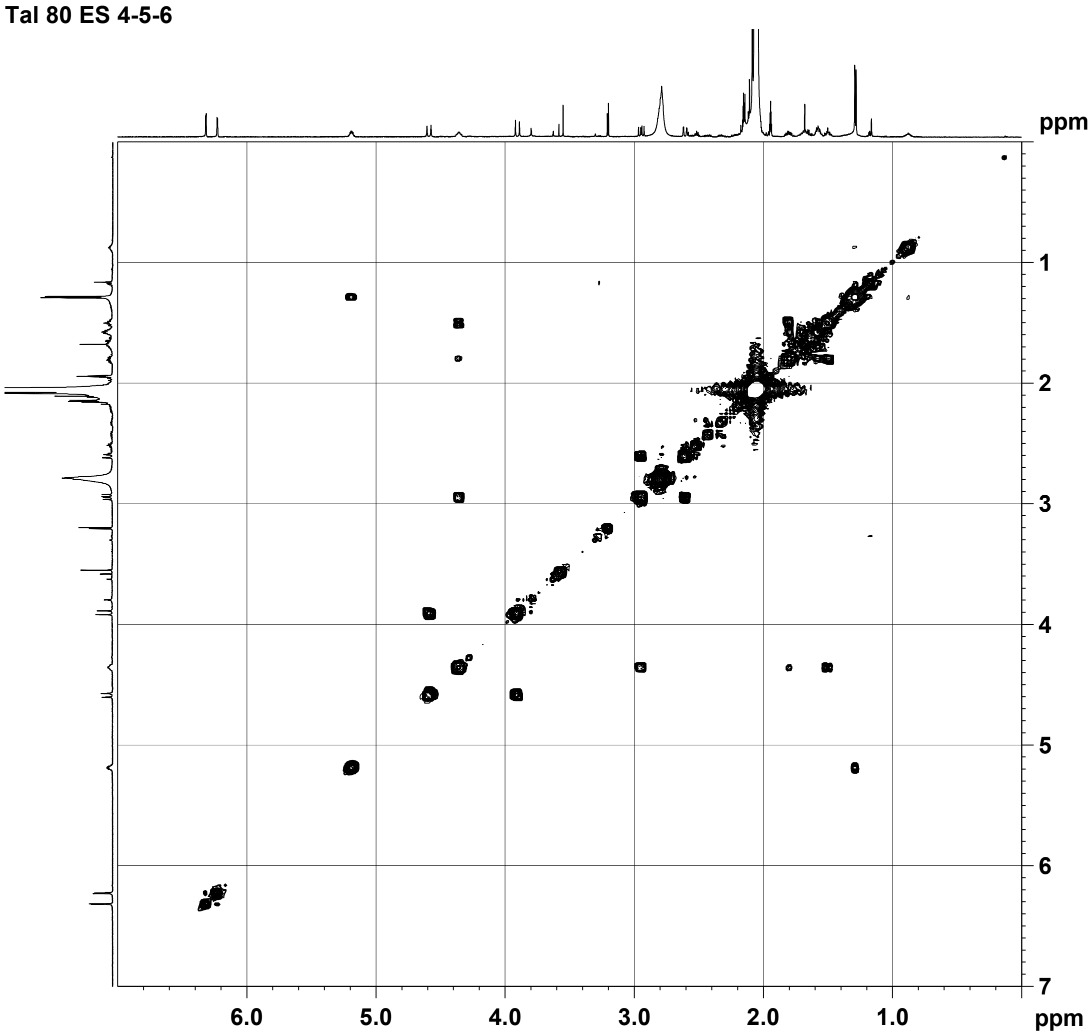

S36. ^{1}H-^{1}H COSY (600 MHz, CD_3COCD_3) spectrum of compound **8**.

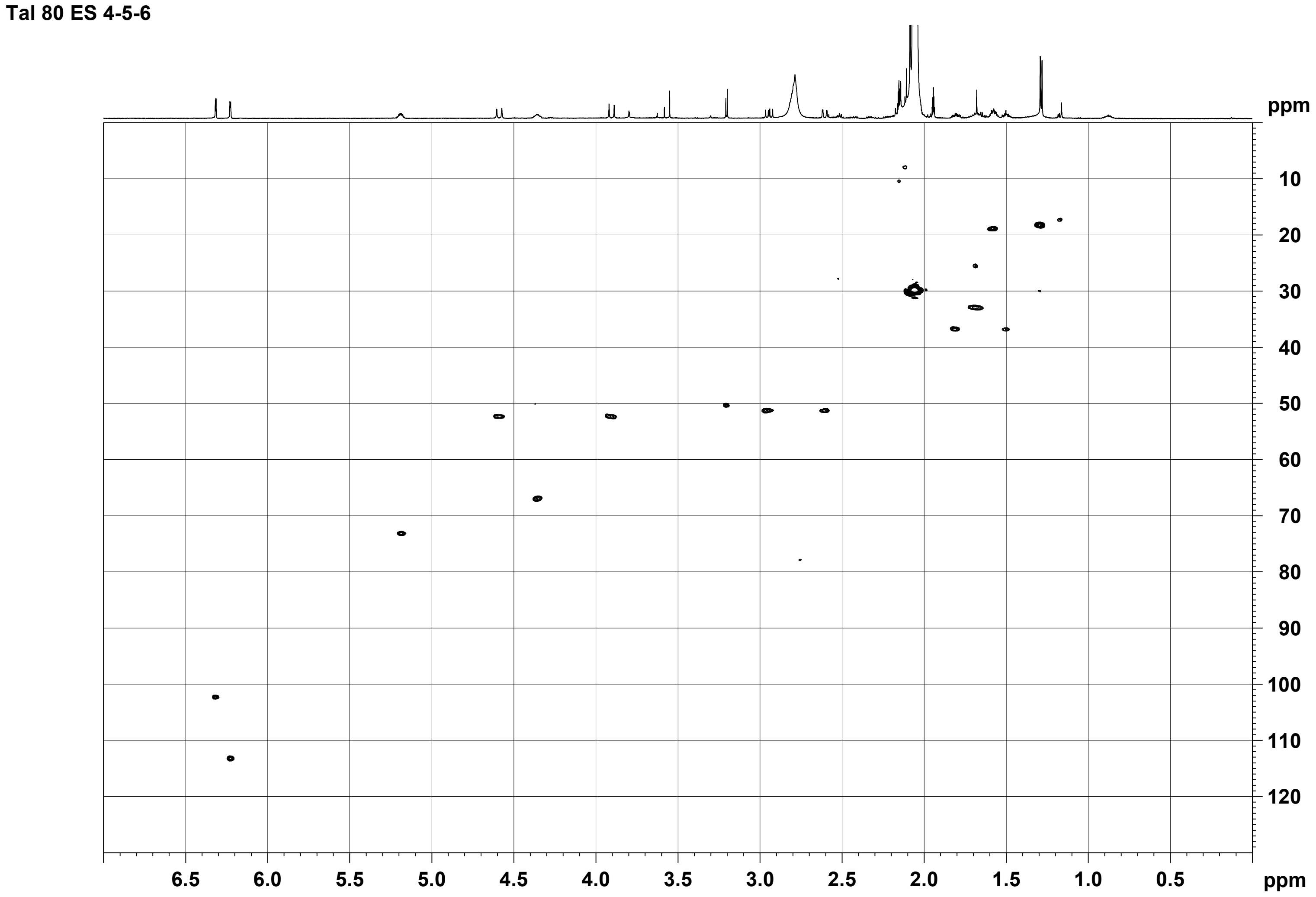

S37. HSQC (600 and 150 MHz, CD_3COCD_3) spectrum of compound **8**.

Tal 80 ES 4-5-6

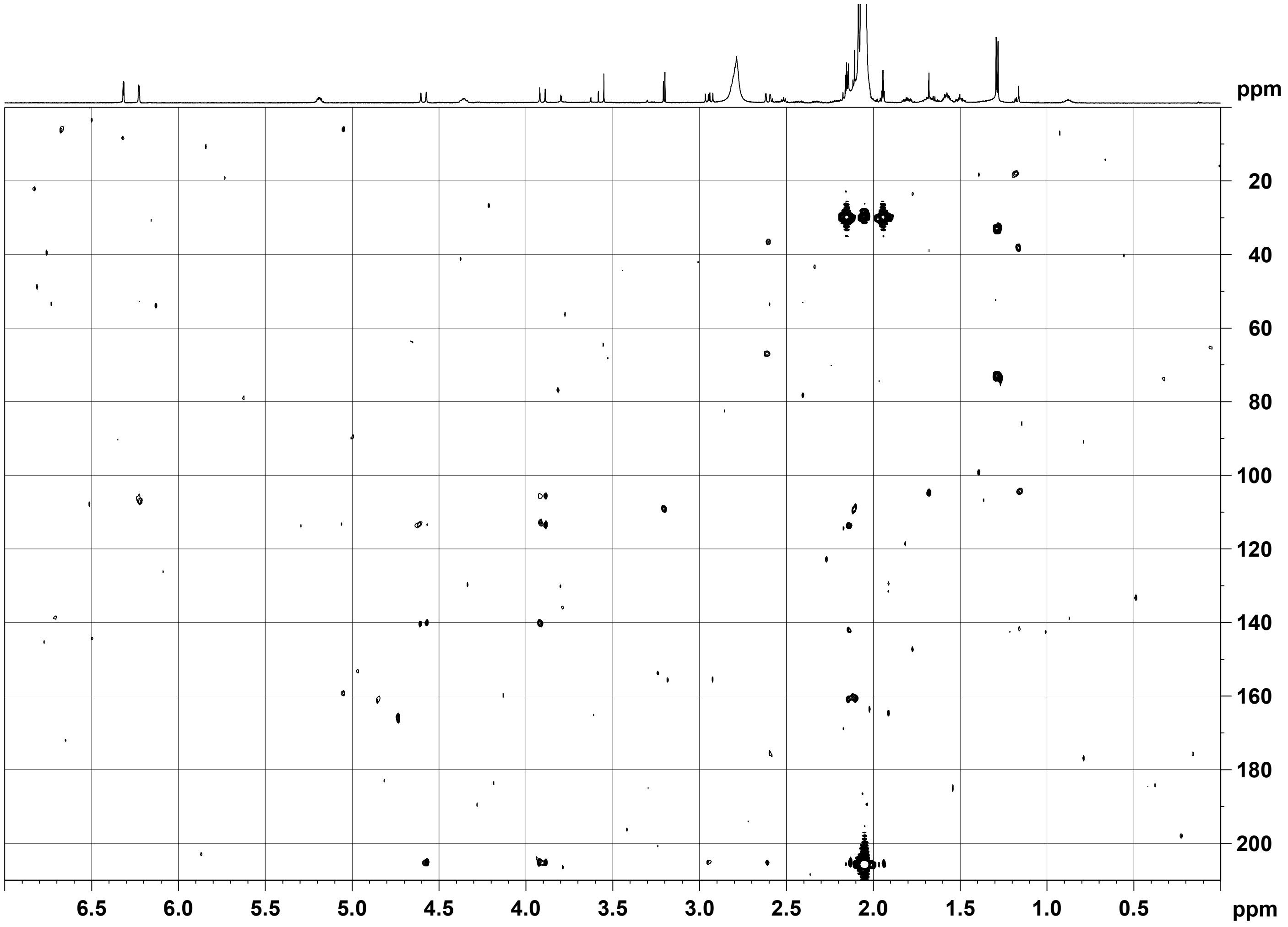

S38. HMBC (600 and 150 MHz, CD_3COCD_3) spectrum of compound **8**.

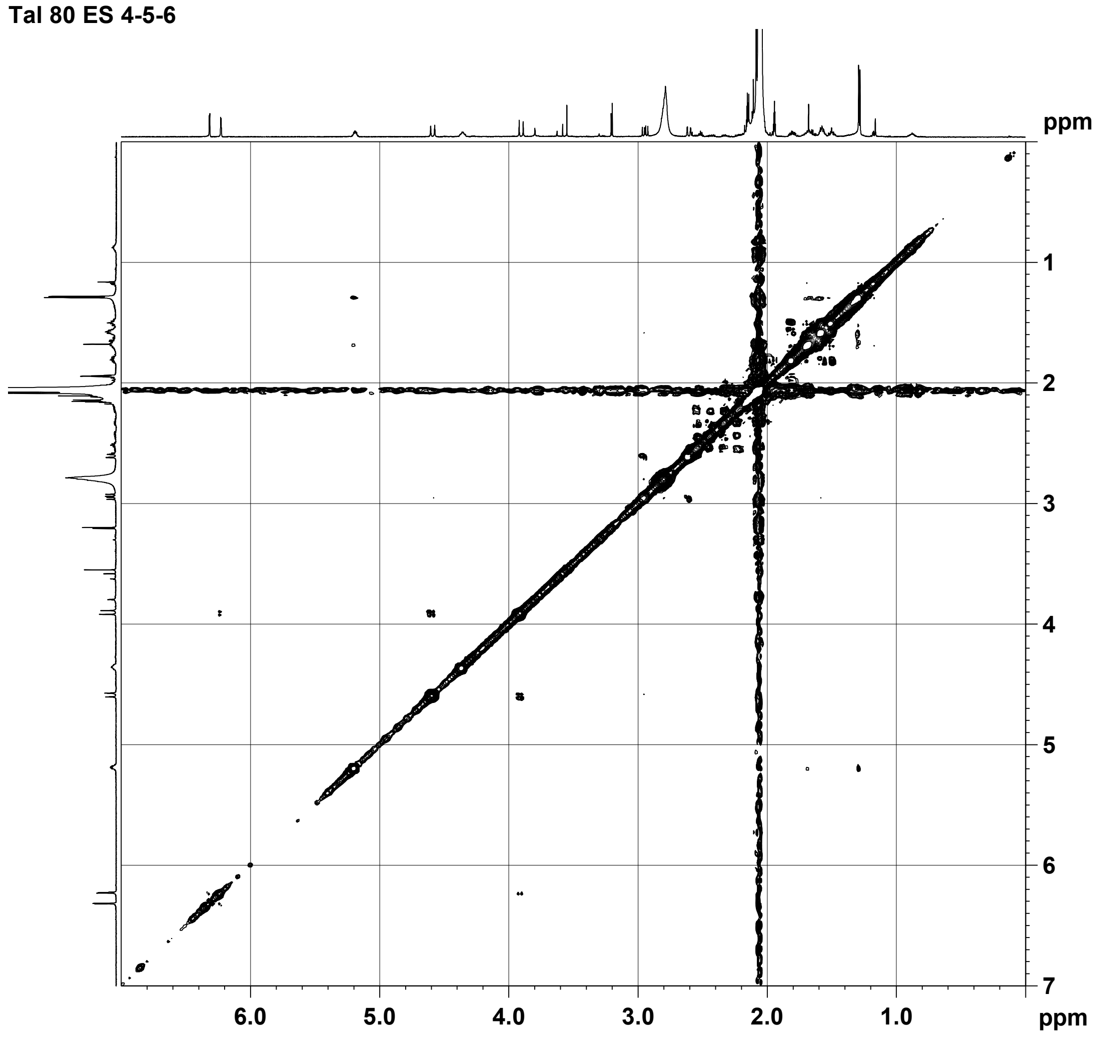

S39. ROESY (600 MHz, CD_3COCD_3) spectrum of compound **8**.

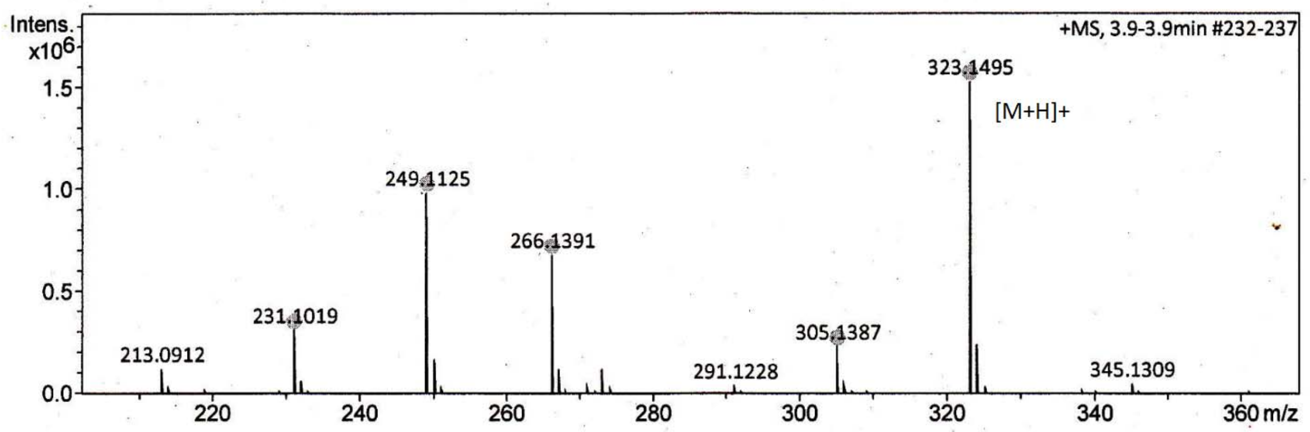

Meas. m/z	#	Ion Formula	m/z	err [ppm]	mSigma	# mSigma	Score	rdb	e¯ Conf	N-Rule
213.0912	1	C14H13O2	213.0910	-1.0	26.0	1	100.00	8.5	even	ok
231.1019	1	C14H15O3	231.1016	-1.4	25.9	1	100.00	7.5	even	ok
249.1125	1	C14H17O4	249.1121	-1.5	8.2	1	100.00	6.5	even	ok
266.1391	1	C14H20NO4	266.1387	-1.7	9.7	1	100.00	5.5	even	ok
305.1387	1	C17H21O5	305.1384	-1.3	9.0	1	100.00	7.5	even	ok
323.1495	1	C17H23O6	323.1489	-1.9	19.6	1	100.00	6.5	even	ok

S40. HRESIMS spectrum of compound **9**.

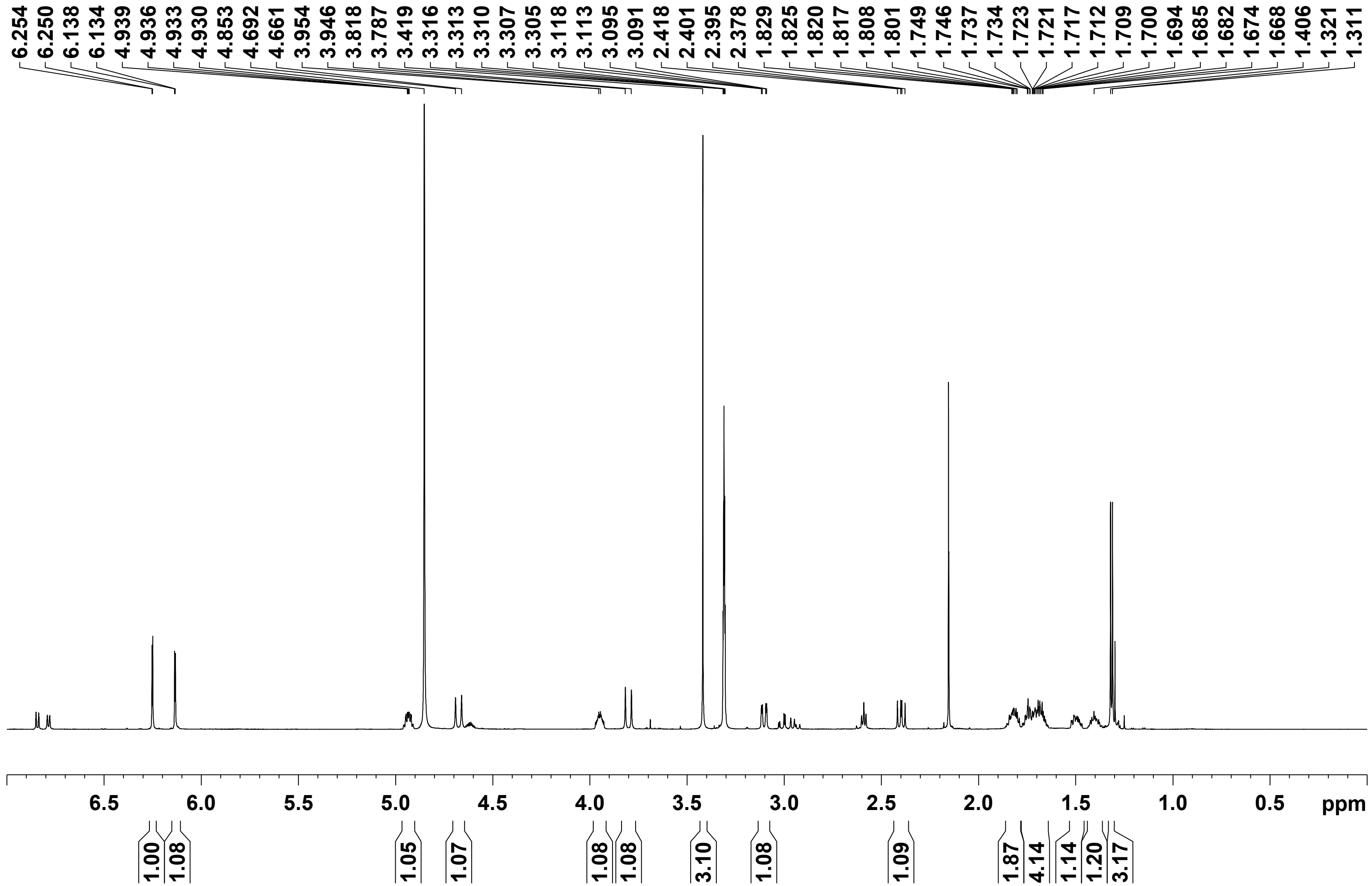

S41. ^{1}H NMR (600 MHz, CD_3OD) spectrum of compound **9**.

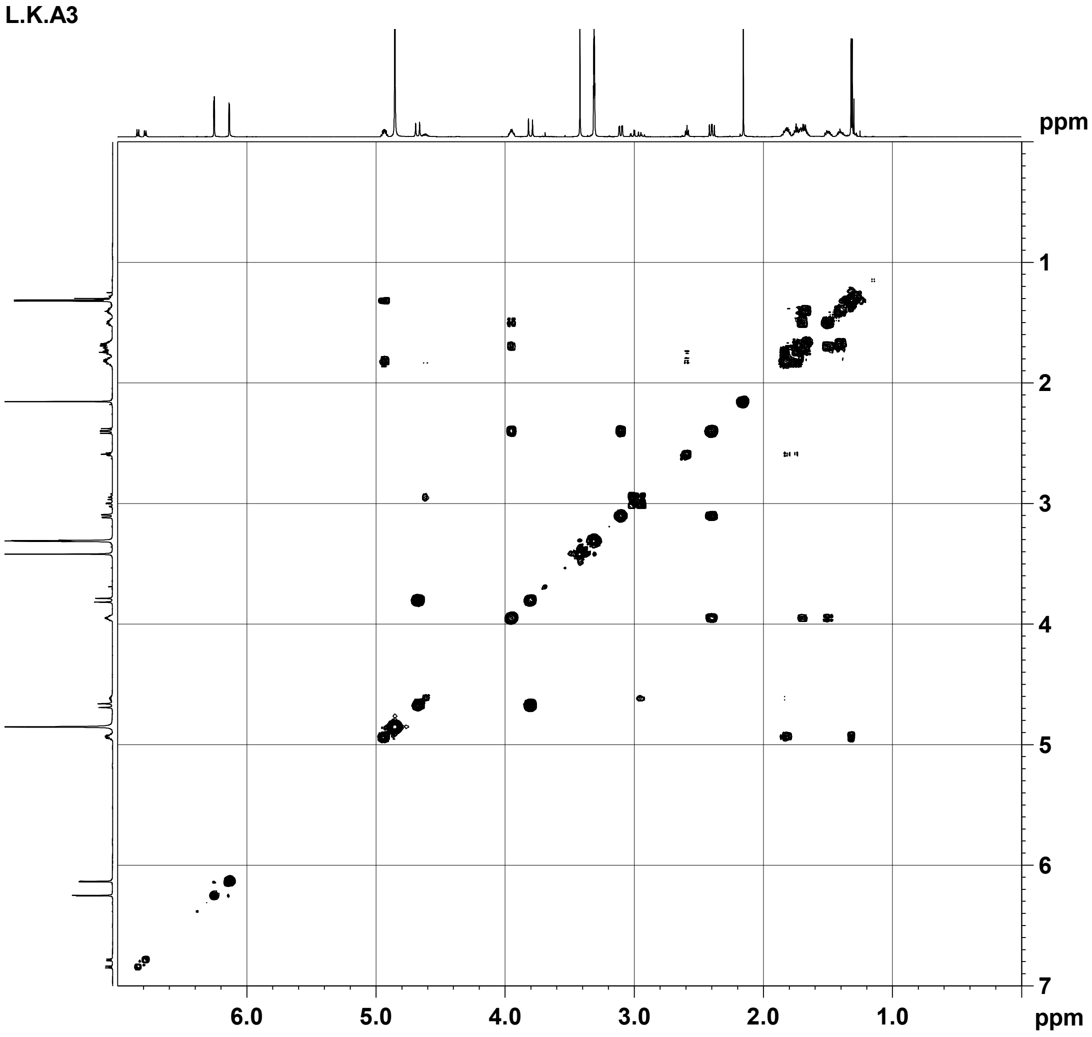

S42. ^{1}H-^{1}H COSY (600 MHz, CD_3OD) spectrum of compound **9**.

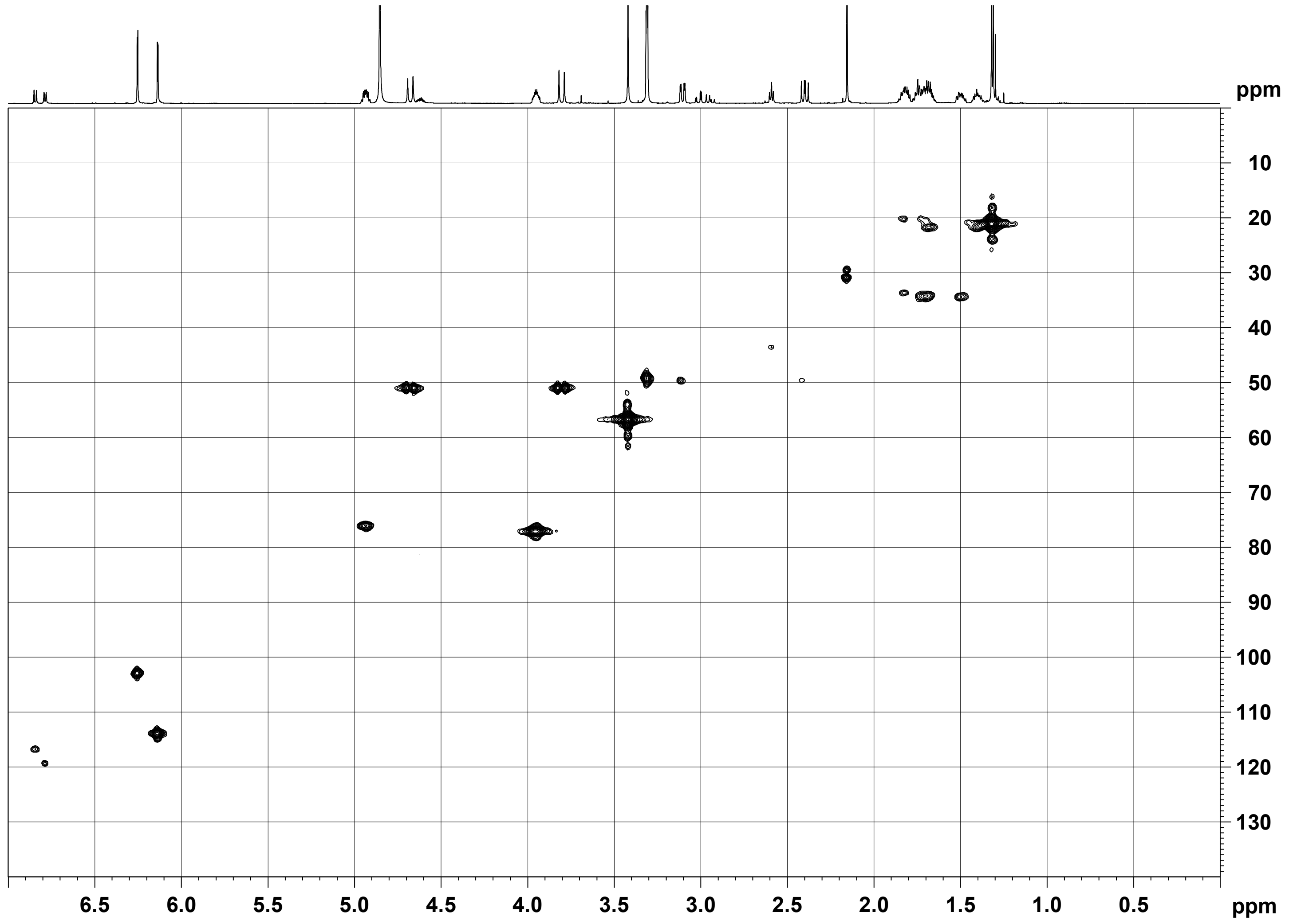

S43. HSQC (600 and 150 MHz, CD_3OD) spectrum of compound **9**.

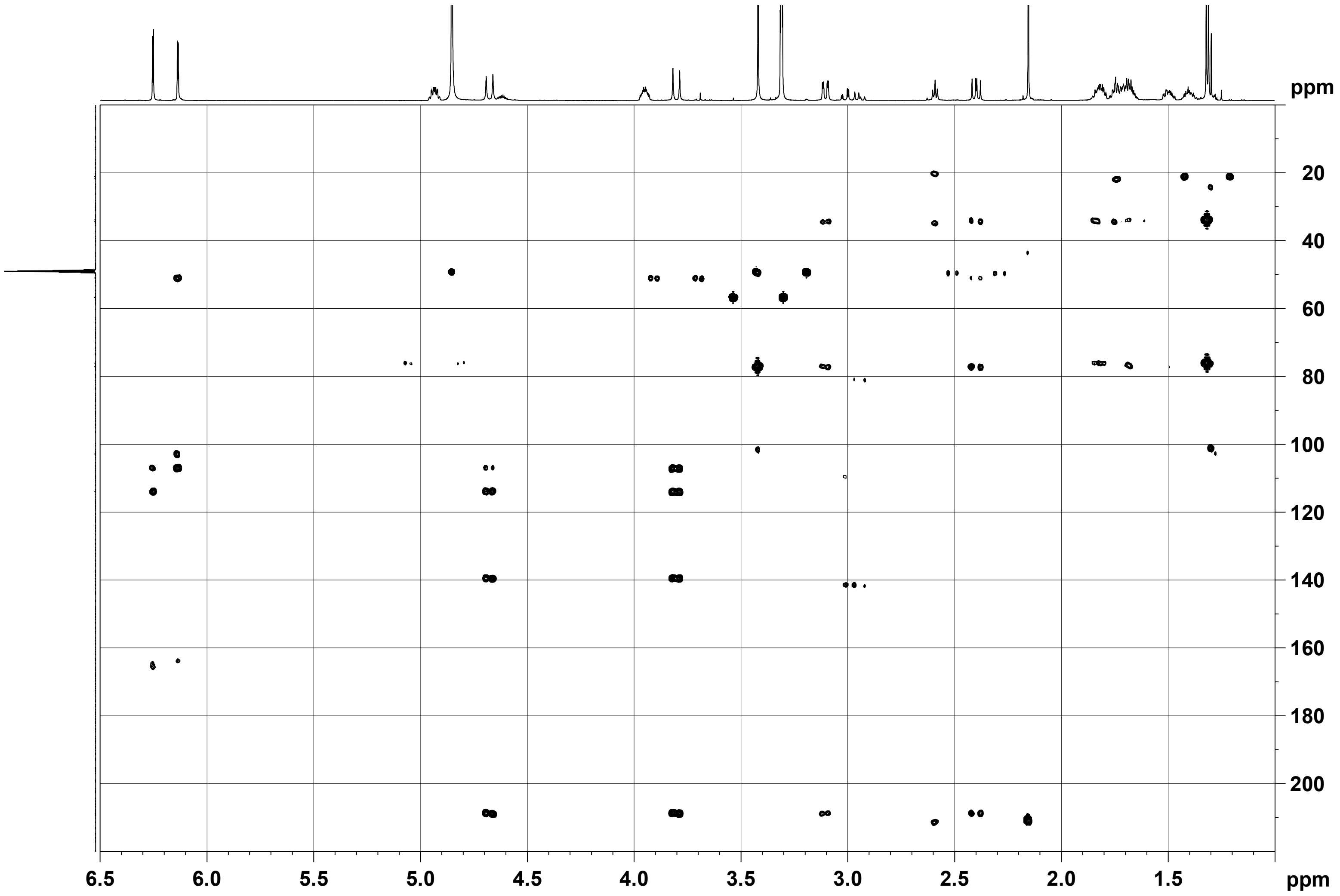

S44. HMBC (600 and 150 MHz, CD_3OD) spectrum of compound **9**.

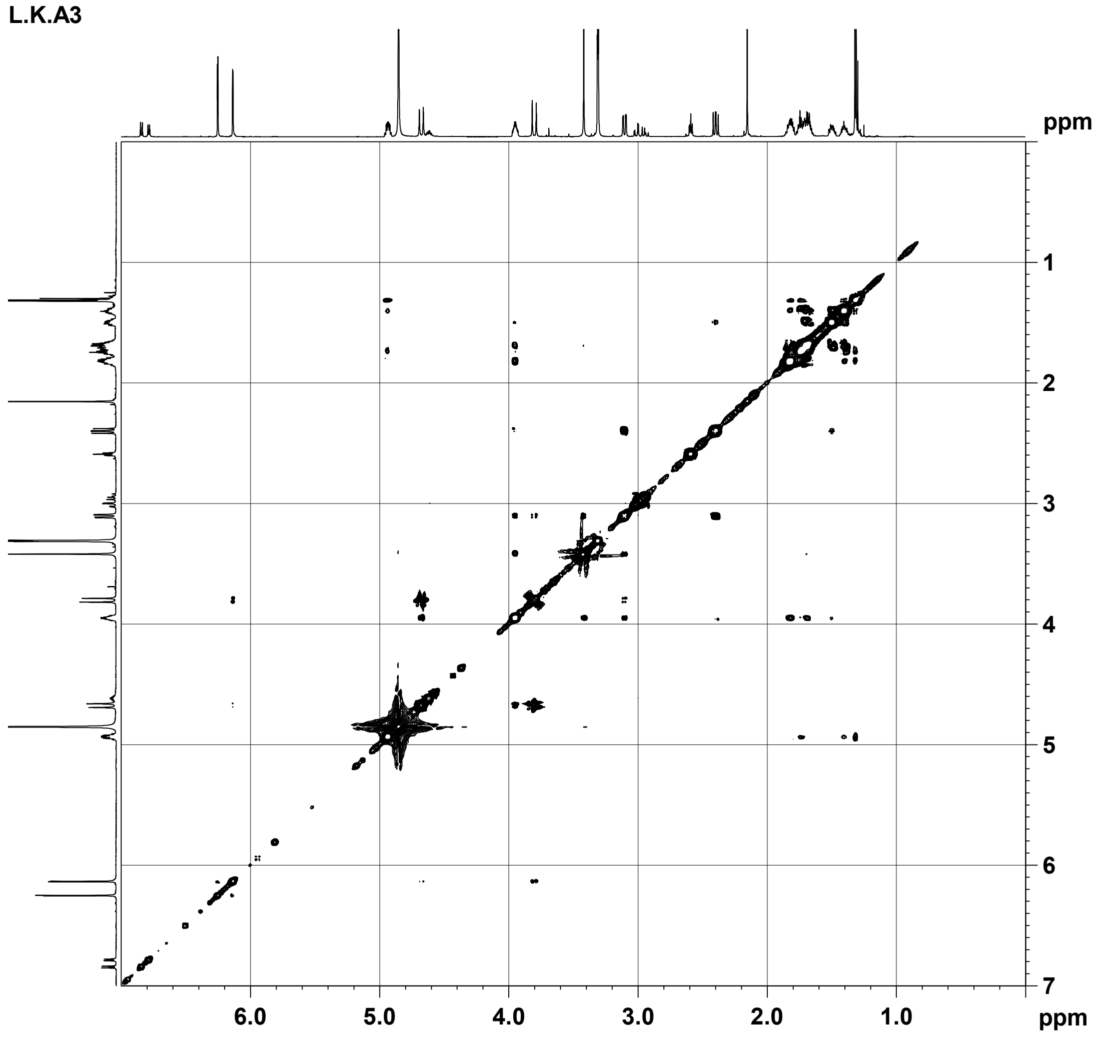

S45. ROESY (600 MHz, CD_3OD) spectrum of compound **9**.

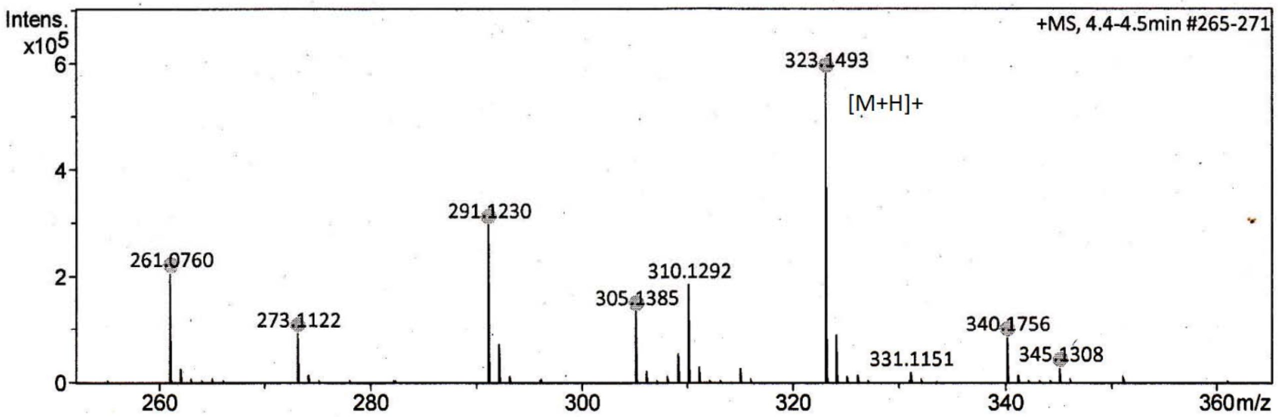

Meas. m/z	#	Ion Formula	m/z	err [ppm]	mSigma	# mSigma	Score	rdb	e⁻ Conf	N-Rule
261.0760	1	C14H13O5	261.0757	-0.8	8.8	1	100.00	8.5	even	ok
273.1122	1	C16H17O4	273.1121	-0.2	1.7	1	100.00	8.5	even	ok
291.1230	1	C16H19O5	291.1227	-1.0	43.0	1	100.00	7.5	even	ok
305.1385	1	C17H21O5	305.1384	-0.4	9.4	1	100.00	7.5	even	ok
323.1493	1	C17H23O6	323.1489	-1.2	18.6	1	100.00	6.5	even	ok
340.1756	1	C17H26NO6	340.1755	-0.3	3.0	1	100.00	5.5	even	ok
345.1308	1	C17H22NaO6	345.1309	0.1	0.7	1	100.00	6.5	even	ok

S46. HRESIMS spectrum of compound **10**.

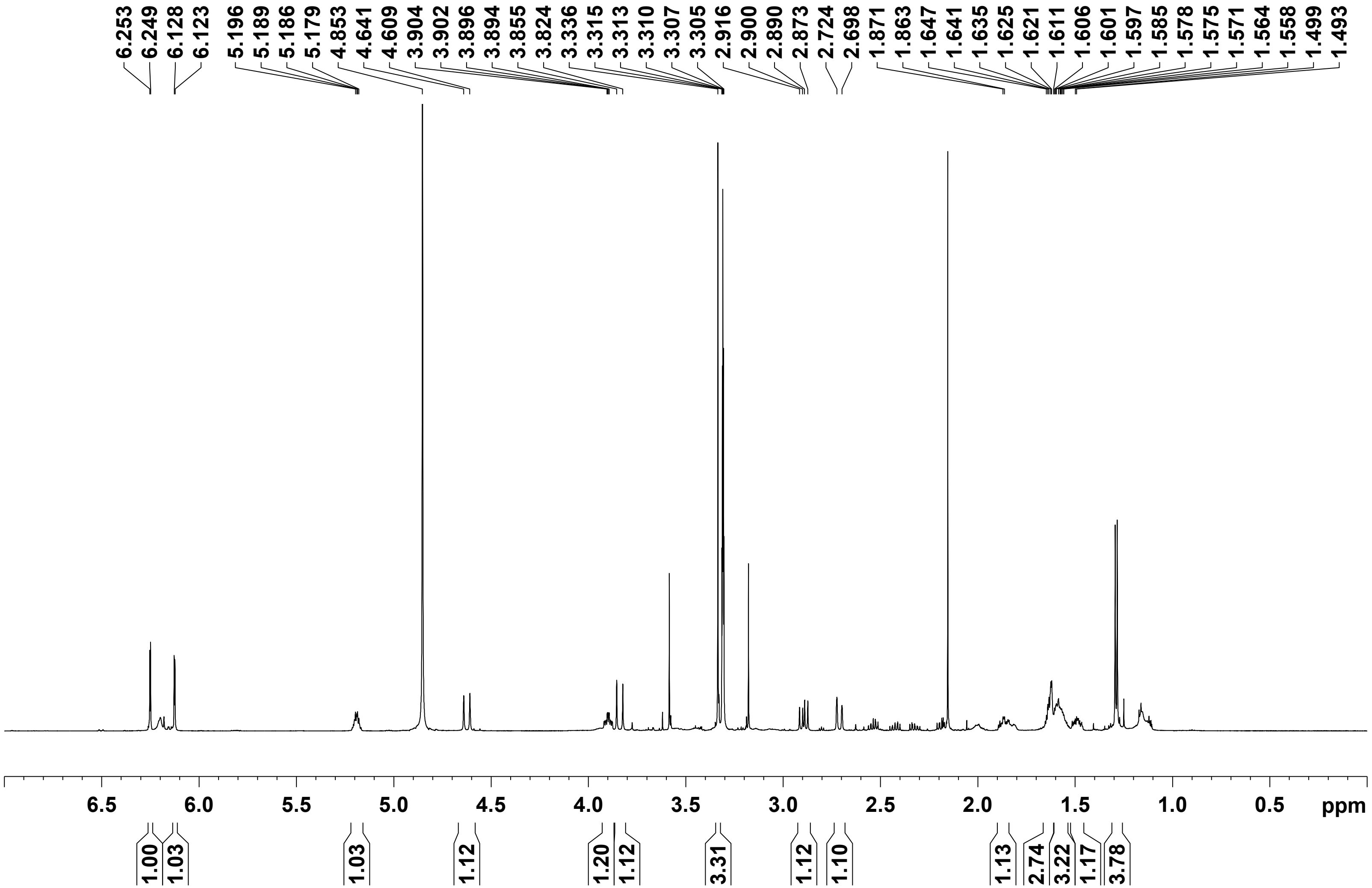

S47. ^{1}H NMR (600 MHz, CD_3OD) spectrum of compound **10**.

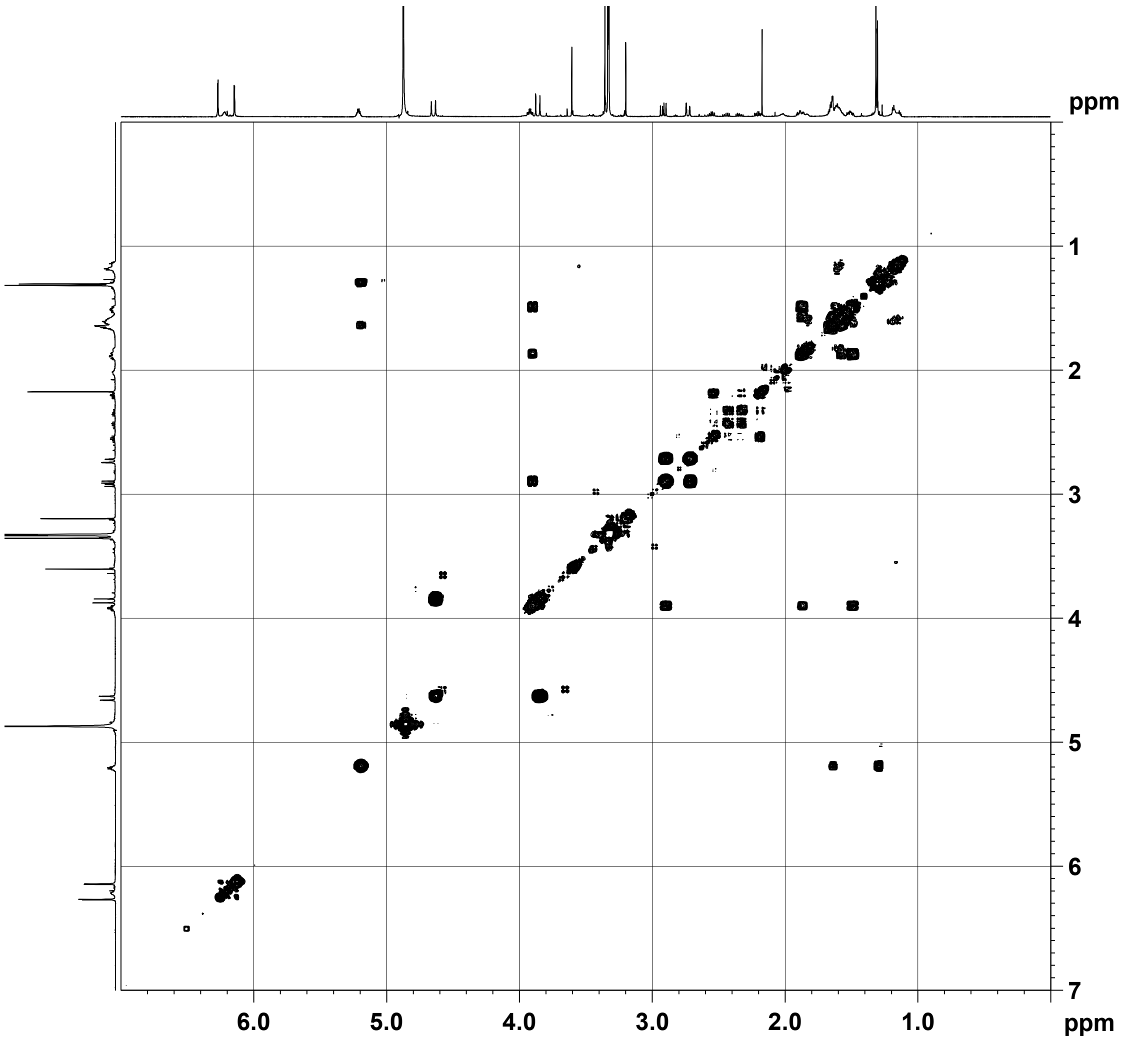

S48. ^{1}H-^{1}H COSY (600 MHz, CD_3OD) spectrum of compound **10**.

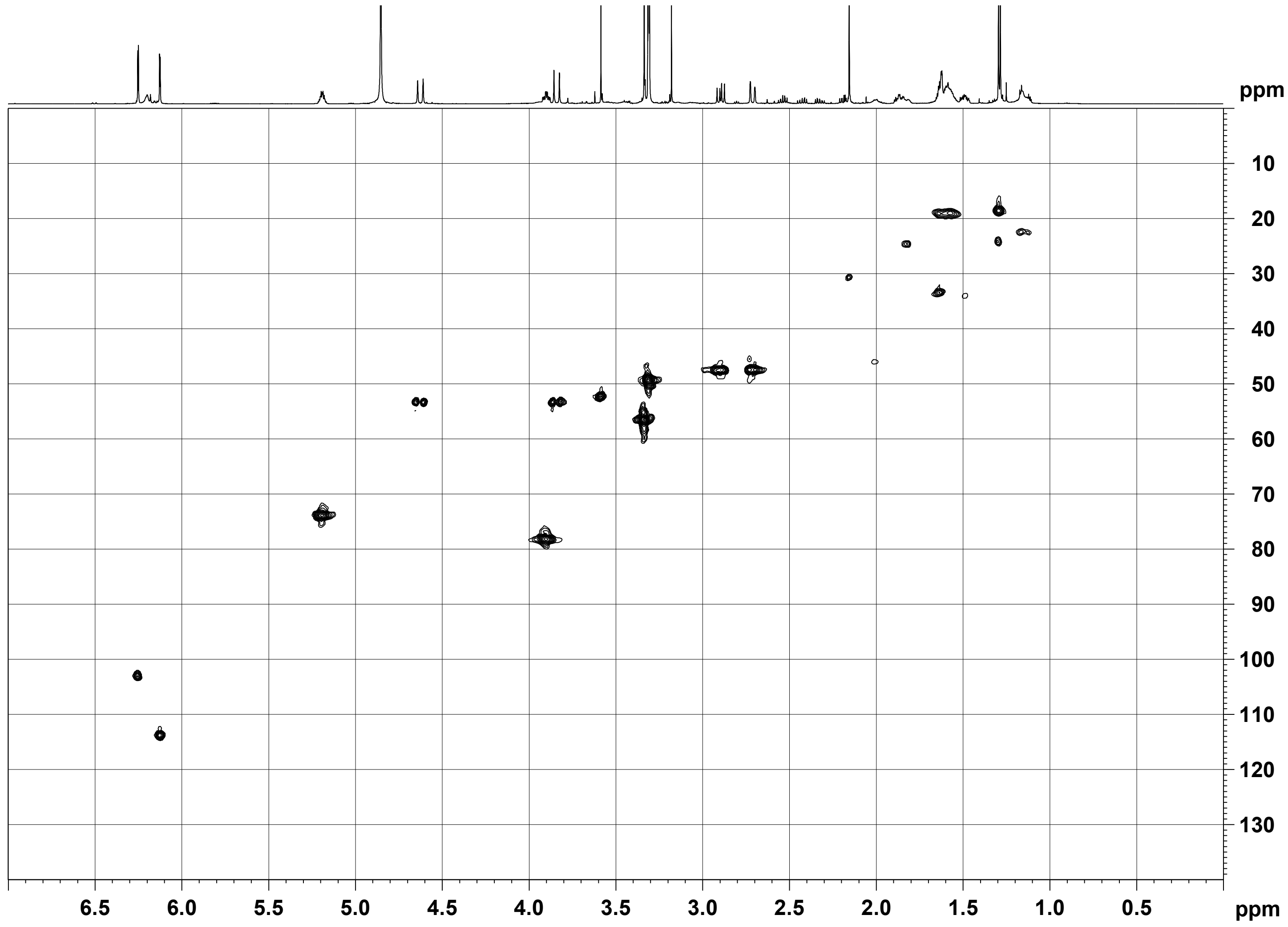

S49. HSQC (600 and 150 MHz, CD_3OD) spectrum of compound **10**.

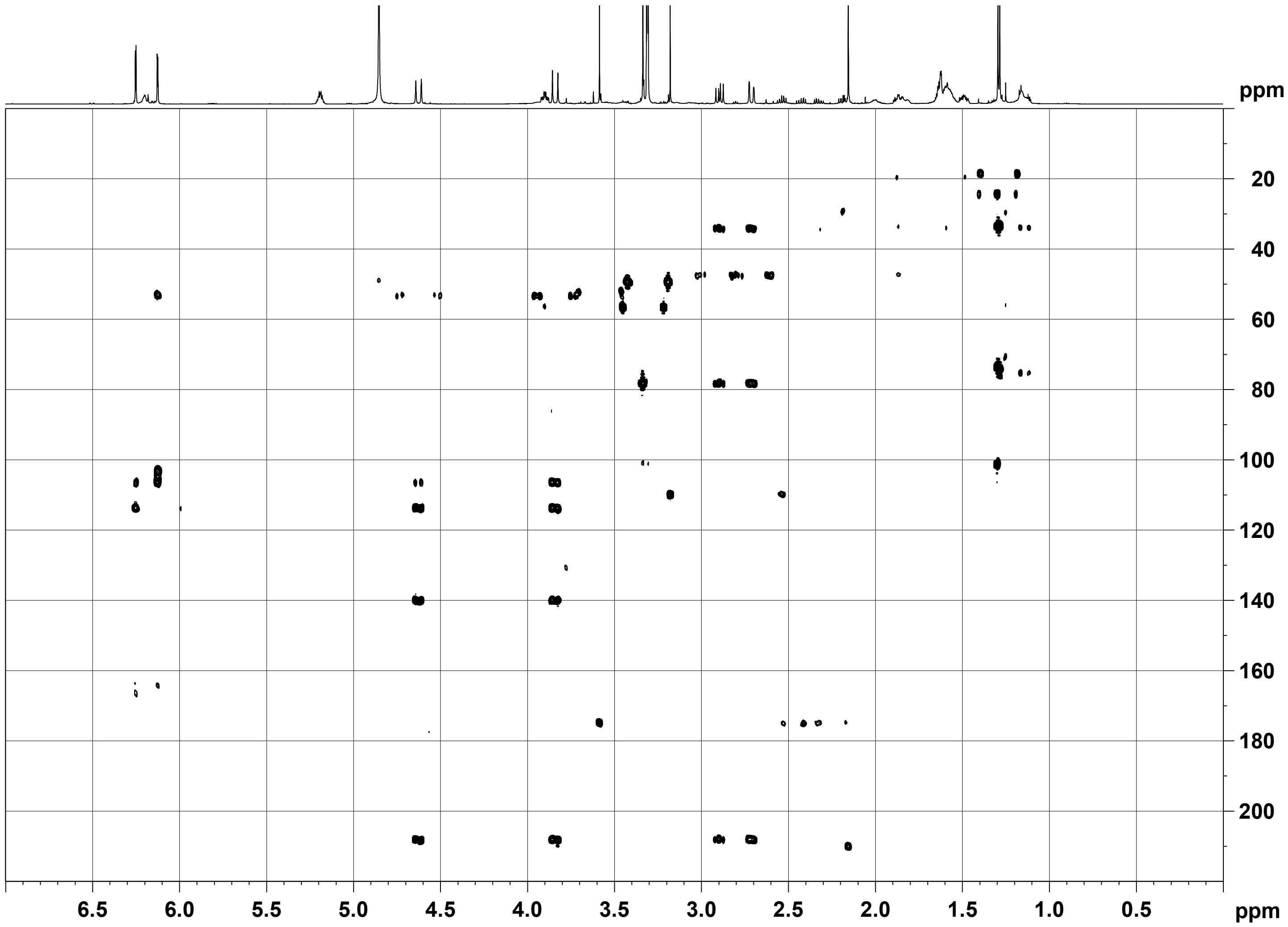

S50. HMBC (600 and 150 MHz, CD_3OD) spectrum of compound **10**.

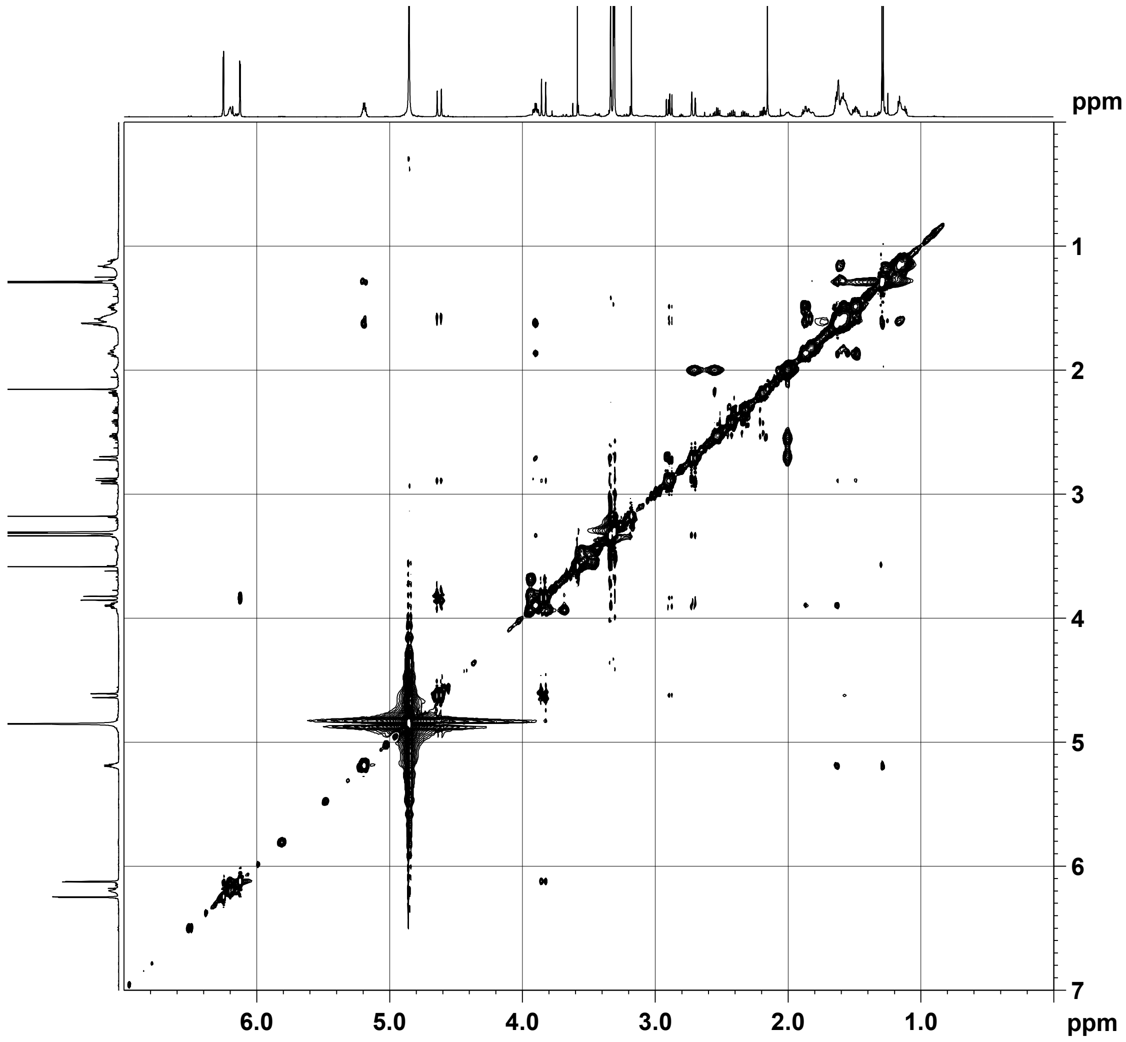

S51. ROESY (600 MHz, CD_3OD) spectrum of compound **10**.

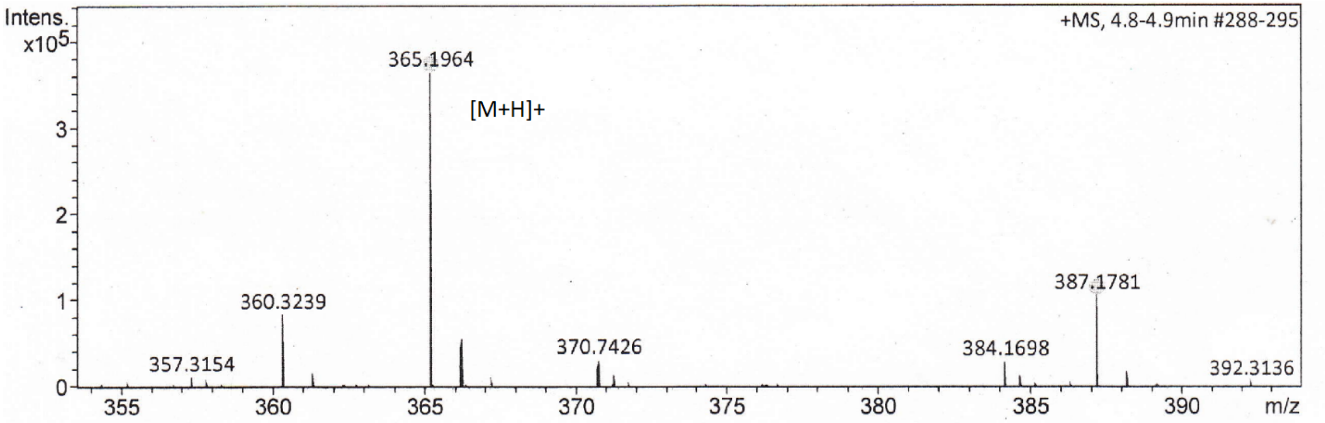

Meas. m/z	#	Ion Formula	m/z	err [ppm]	mSigma	# mSigma	Score	rdb	e¯ Conf	N-Rule
365.1964	1	C20H29O6	365.1959	-1.5	42.1	1	100.00	6.5	even	ok
387.1781	1	C20H28NaO6	387.1778	-0.7	33.3	1	100.00	6.5	even	ok

S52. HRESIMS spectrum of compound **11**.

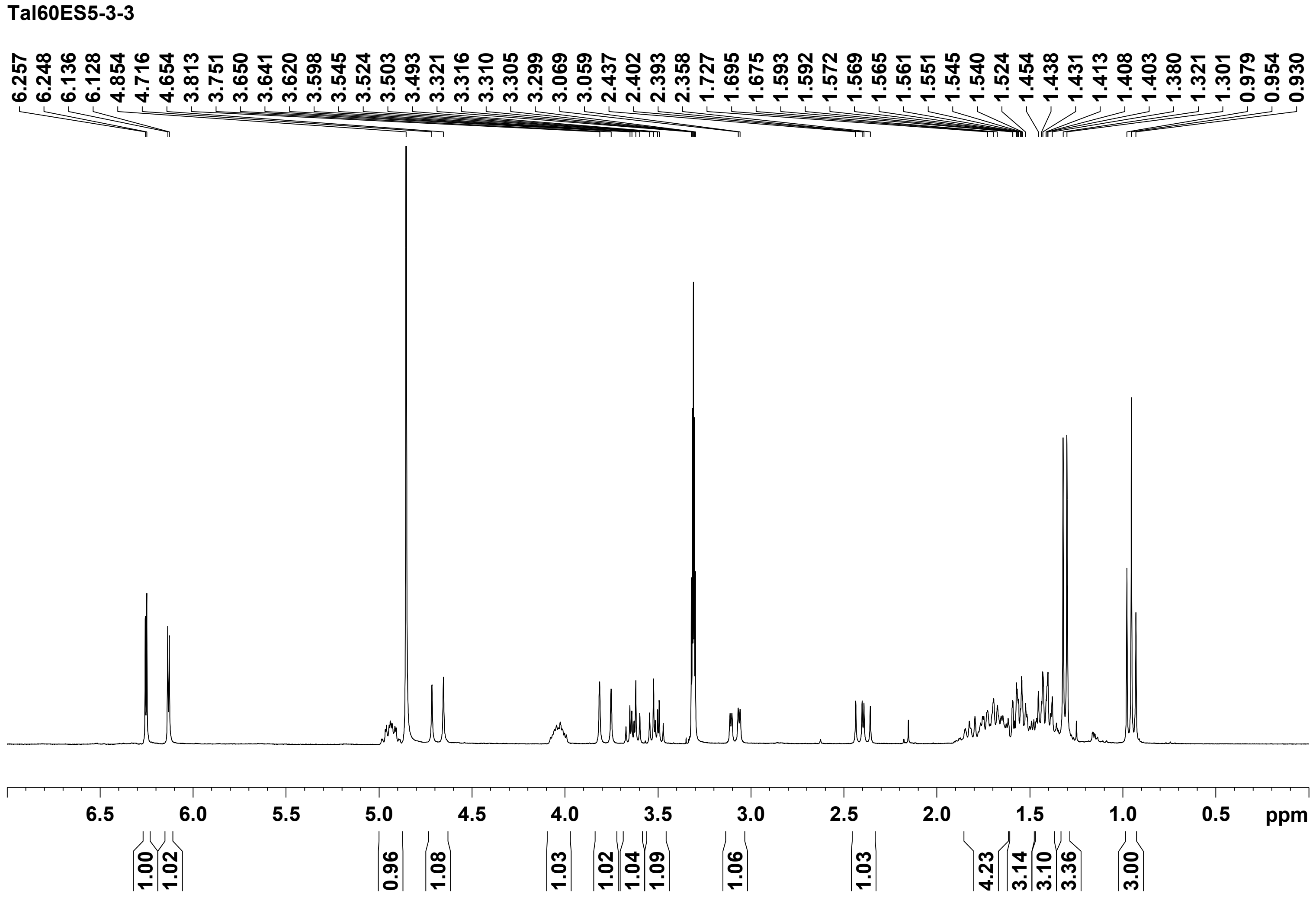

S53. ^{1}H NMR (300 MHz, CD_3OD) spectrum of compound **11**.

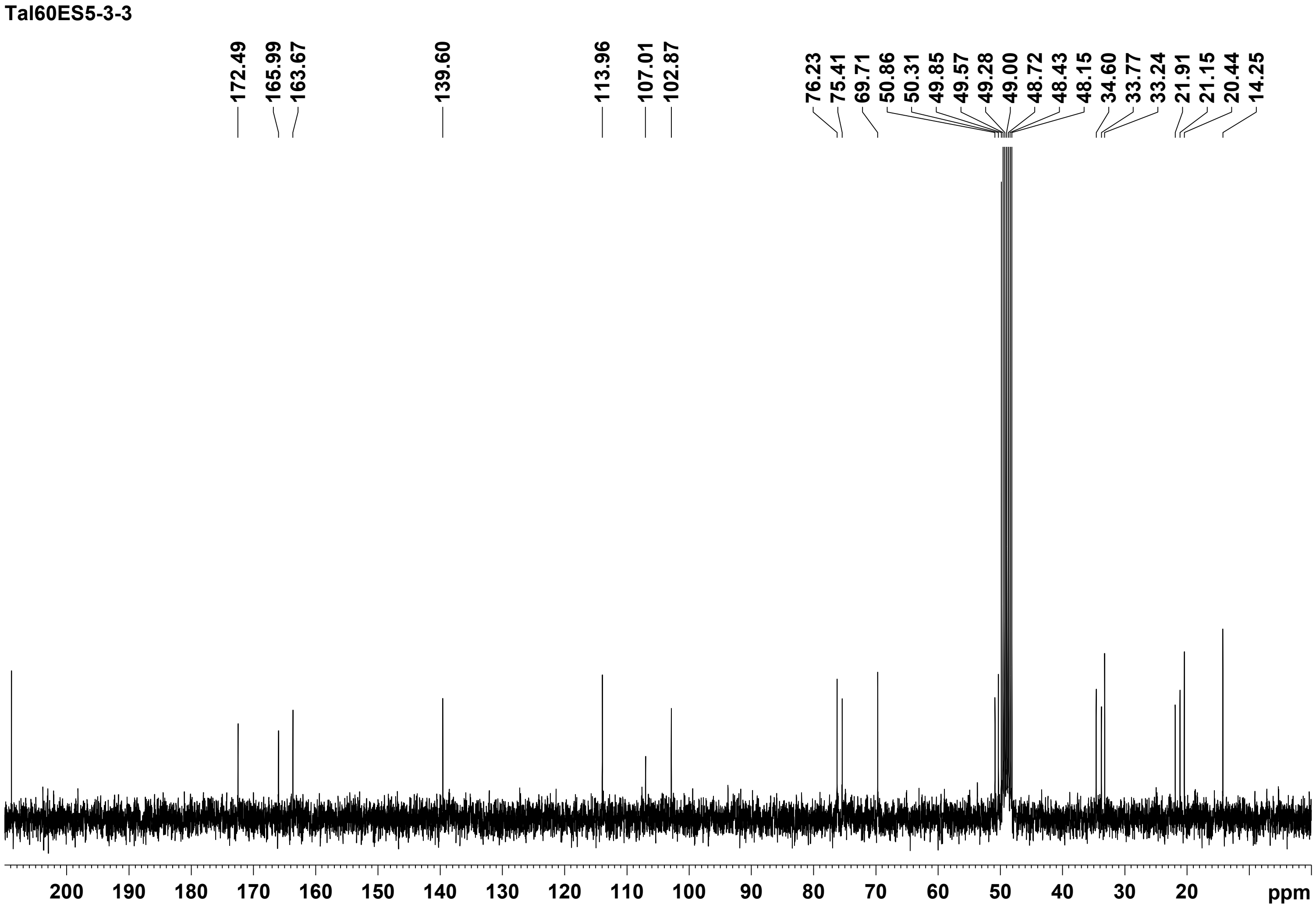

S54. ^{13}C NMR (75 MHz, CD_3OD) spectrum of compound **11**.

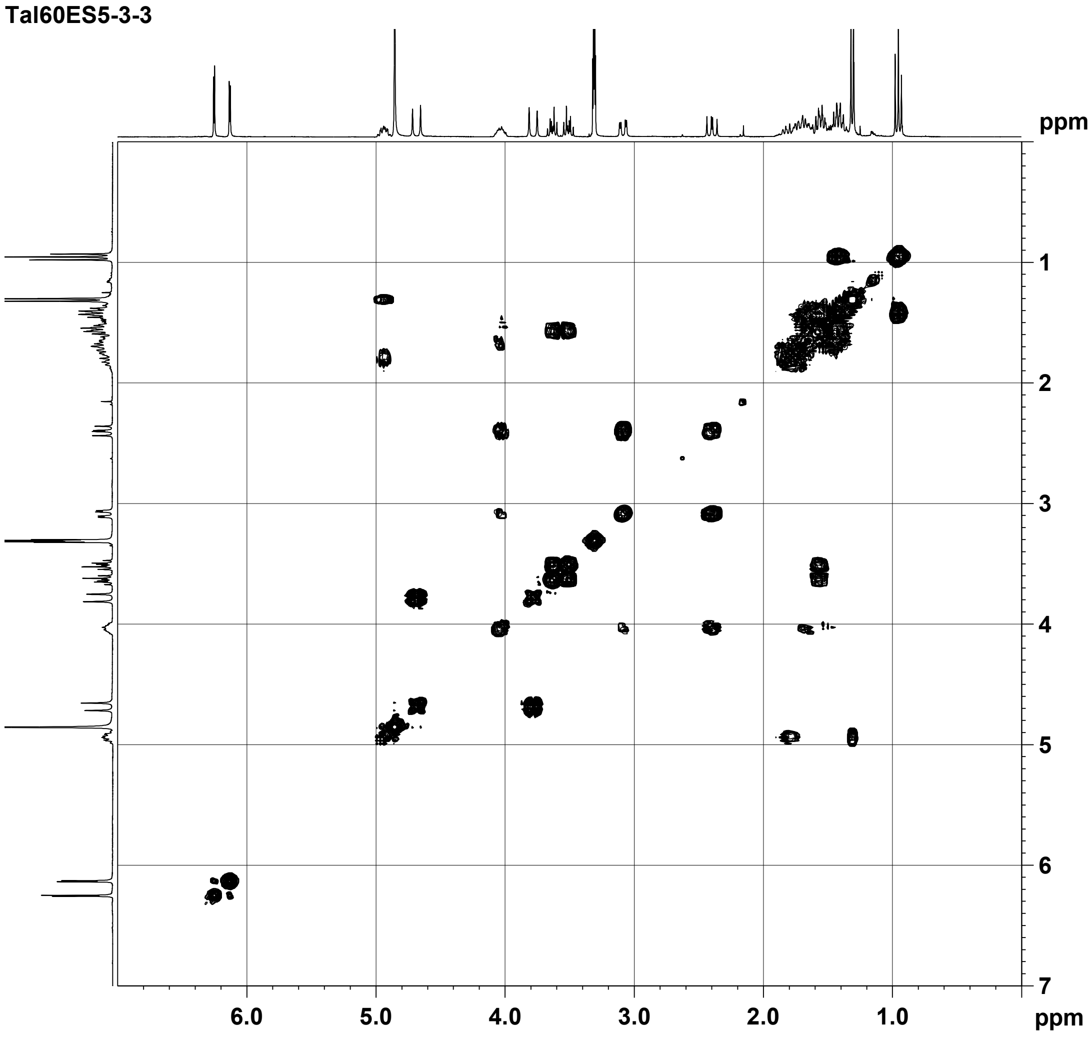

S55. ^{1}H-^{1}H COSY (300 MHz, CD_3OD) spectrum of compound **11**.

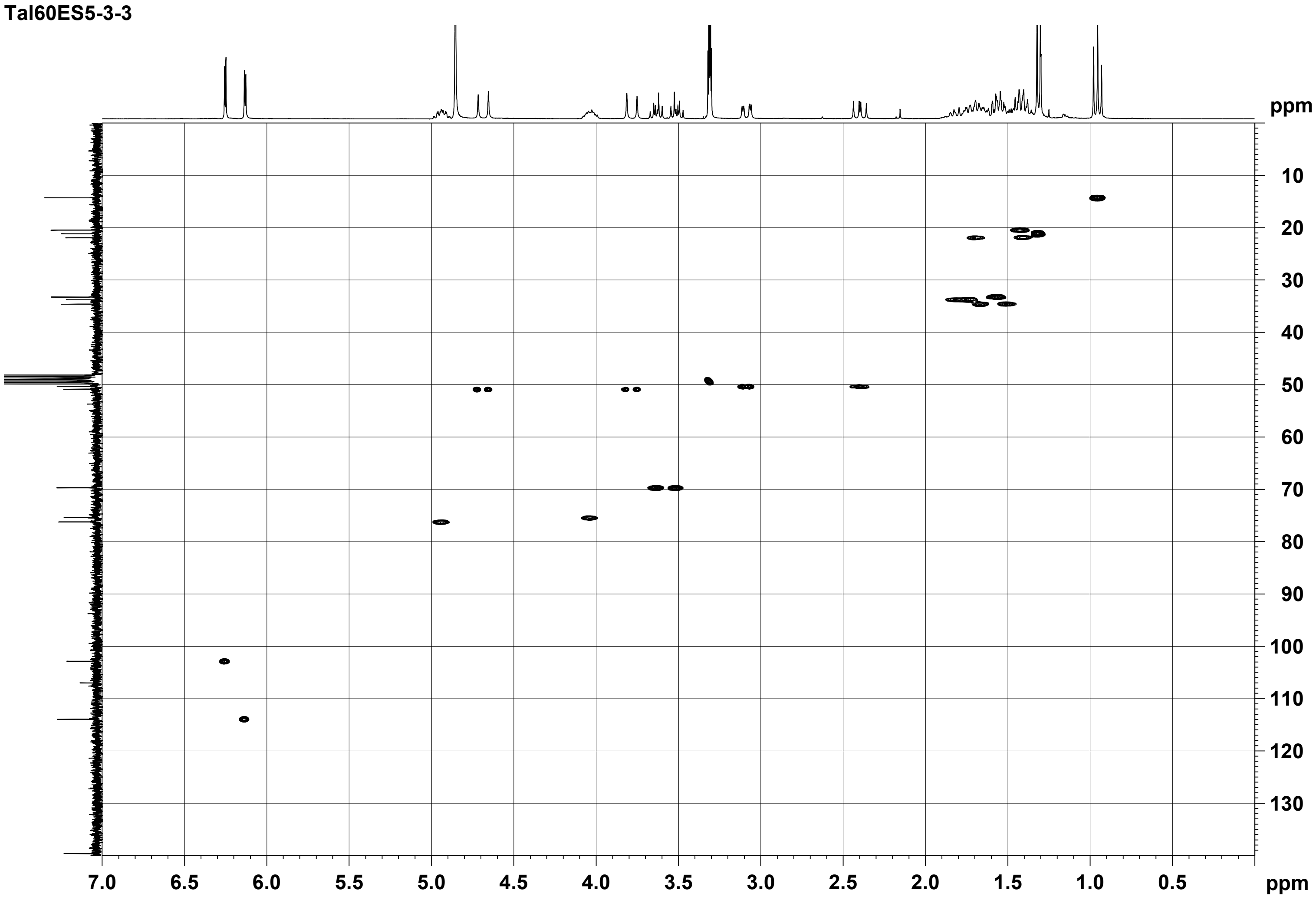

S56. HSQC (300 and 75 MHz, CD_3OD) spectrum of compound **11**.

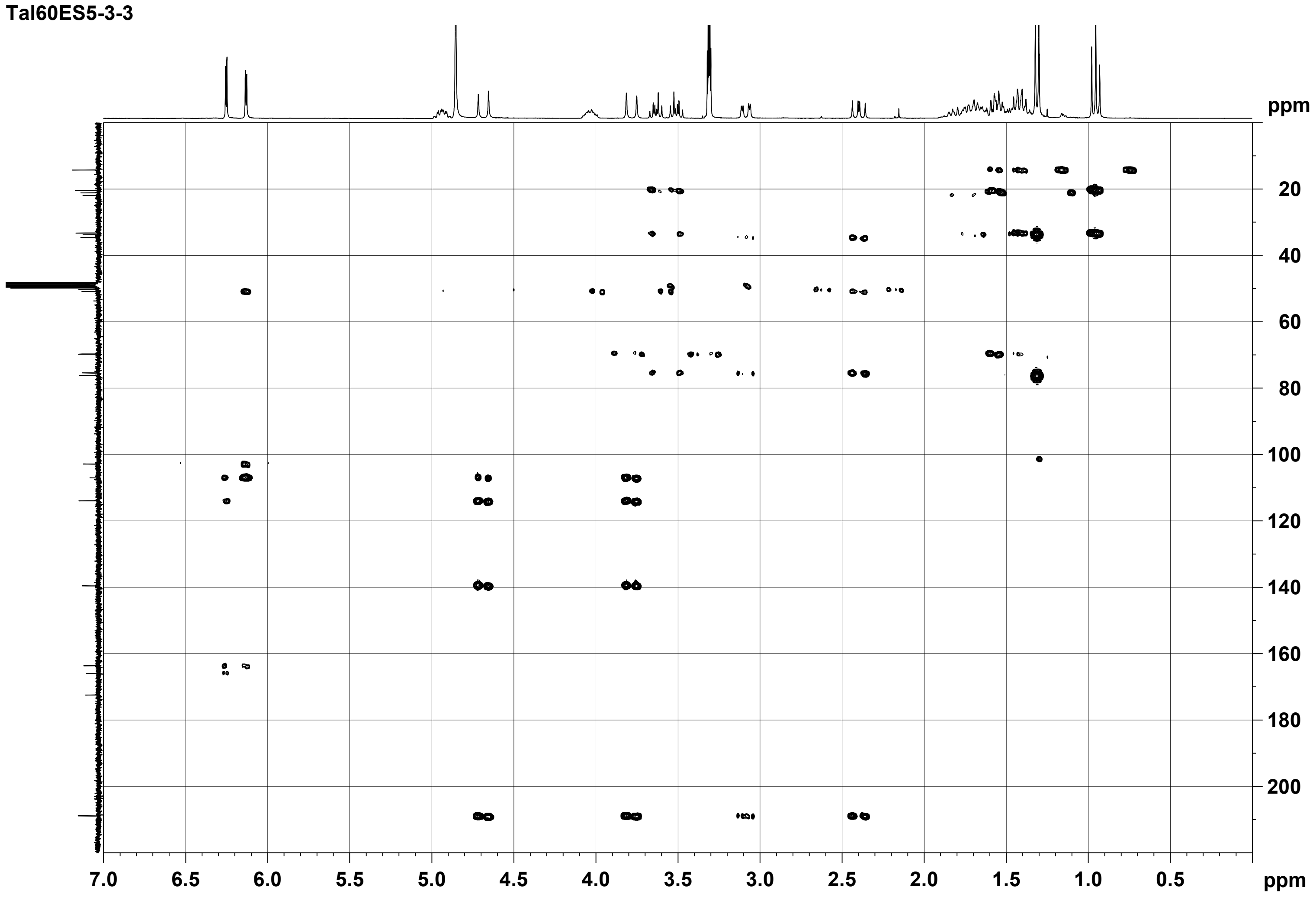

S57. HMBC (300 and 75 MHz, CD_3OD) spectrum of compound **11**.

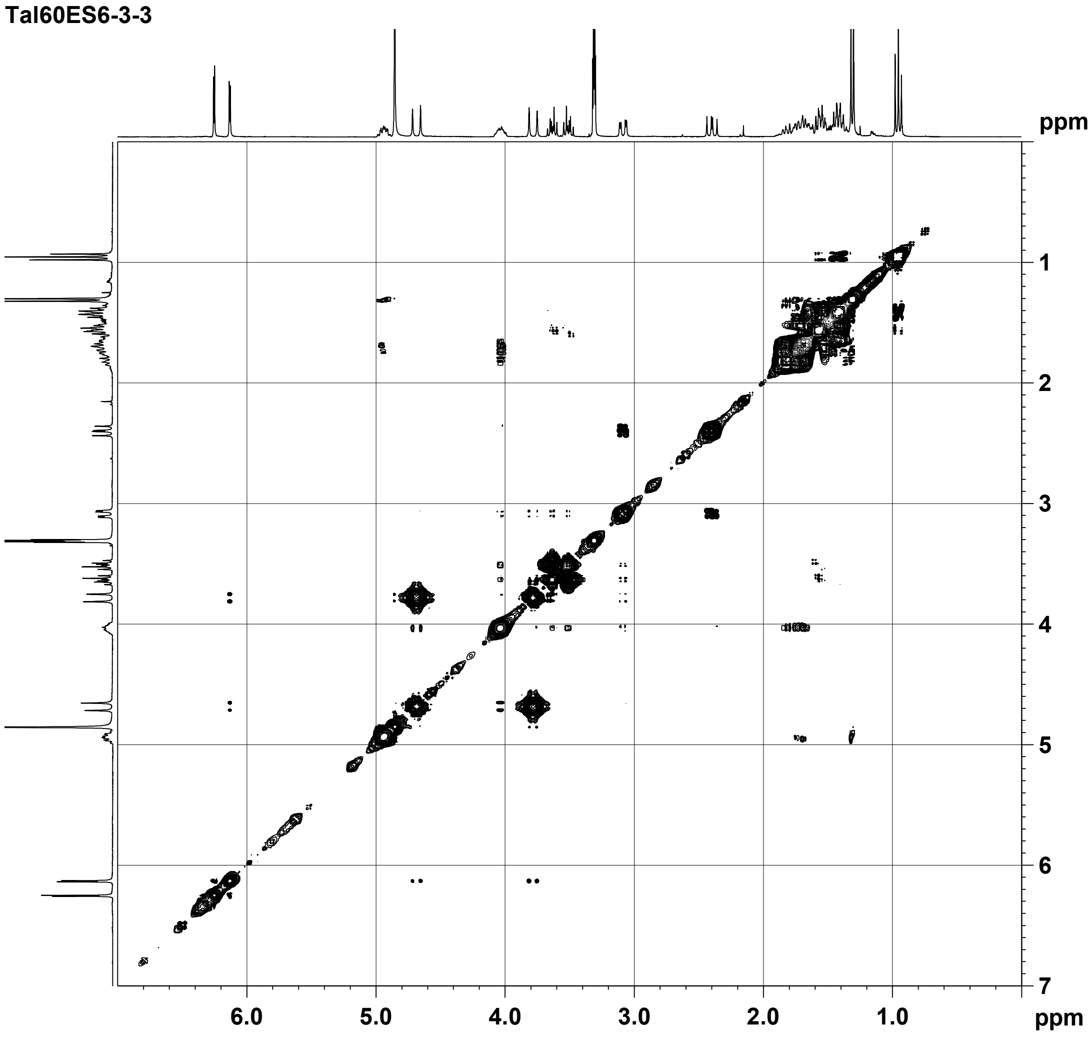

S58. ROESY (300 MHz, CD_3OD) spectrum of compound **11**.

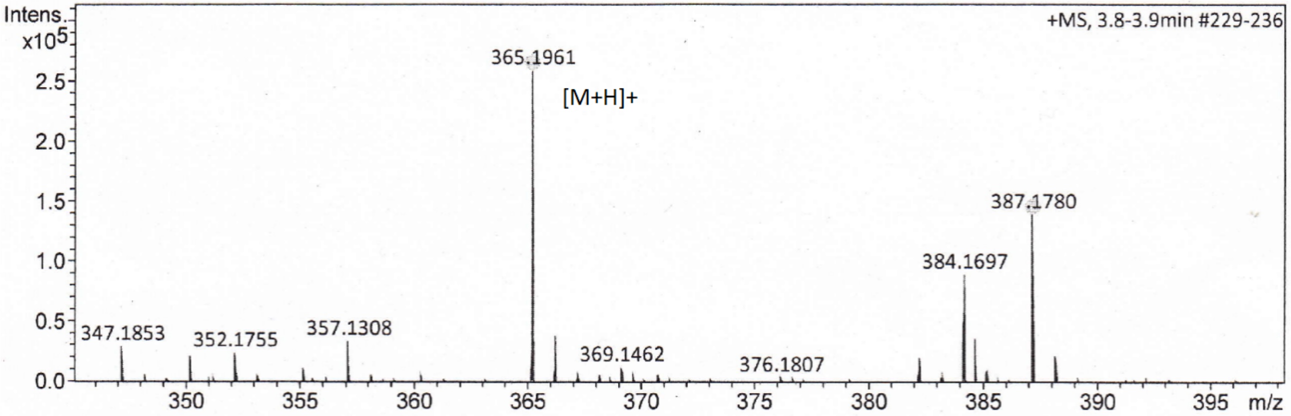

Meas. m/z	#	Ion Formula	m/z	err [ppm]	mSigma	# mSigma	Score	rdb	e⁻ Conf	N-Rule
365.1961	1	C20H29O6	365.1959	-0.8	43.6	1	100.00	6.5	even	ok
387.1780	1	C20H28NaO6	387.1778	-0.6	39.4	1	100.00	6.5	even	ok

S59. HRESIMS spectrum of compound **12**.

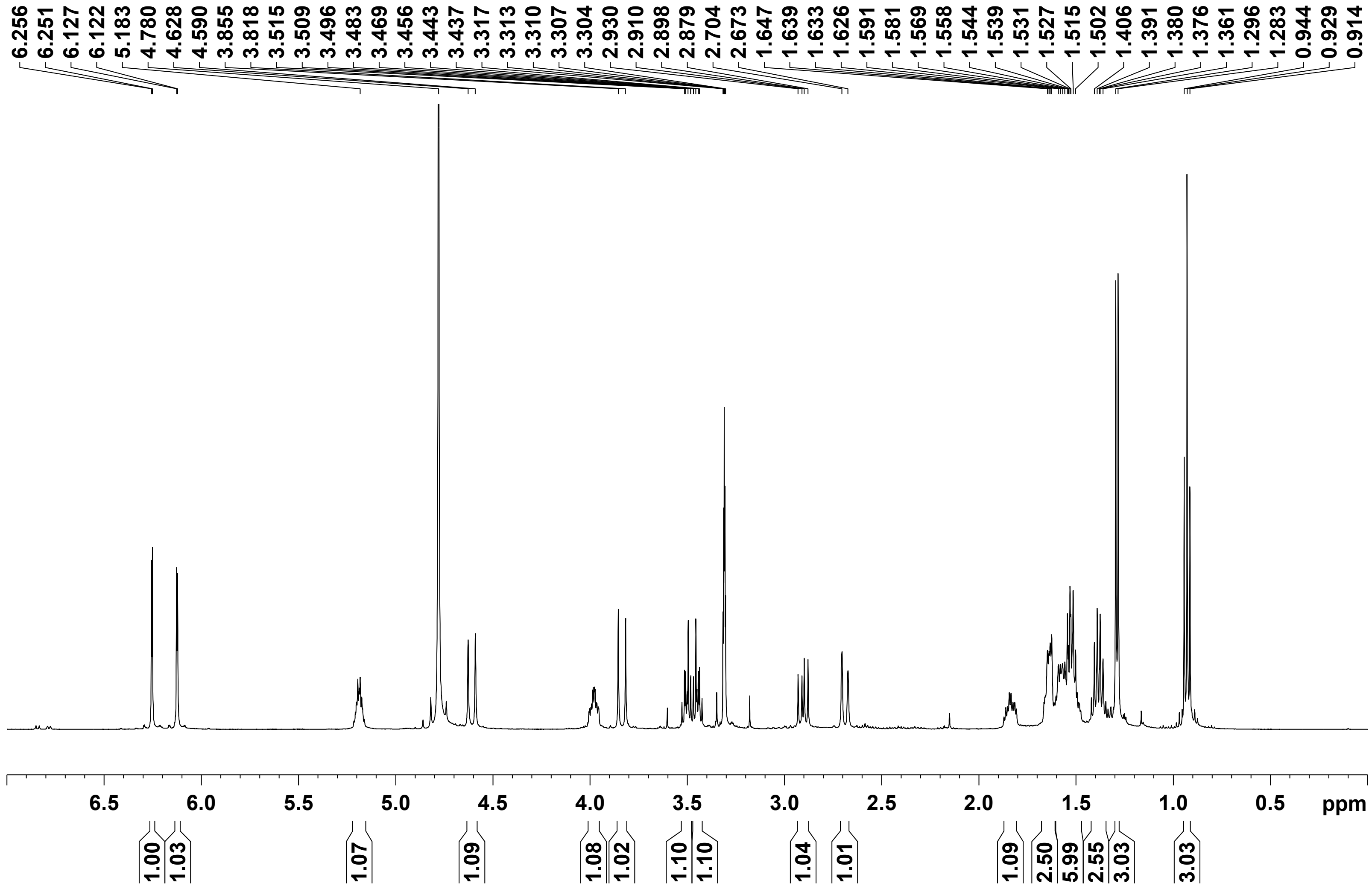

S60. ^{1}H NMR (500 MHz, CD_3OD) spectrum of compound **12**.

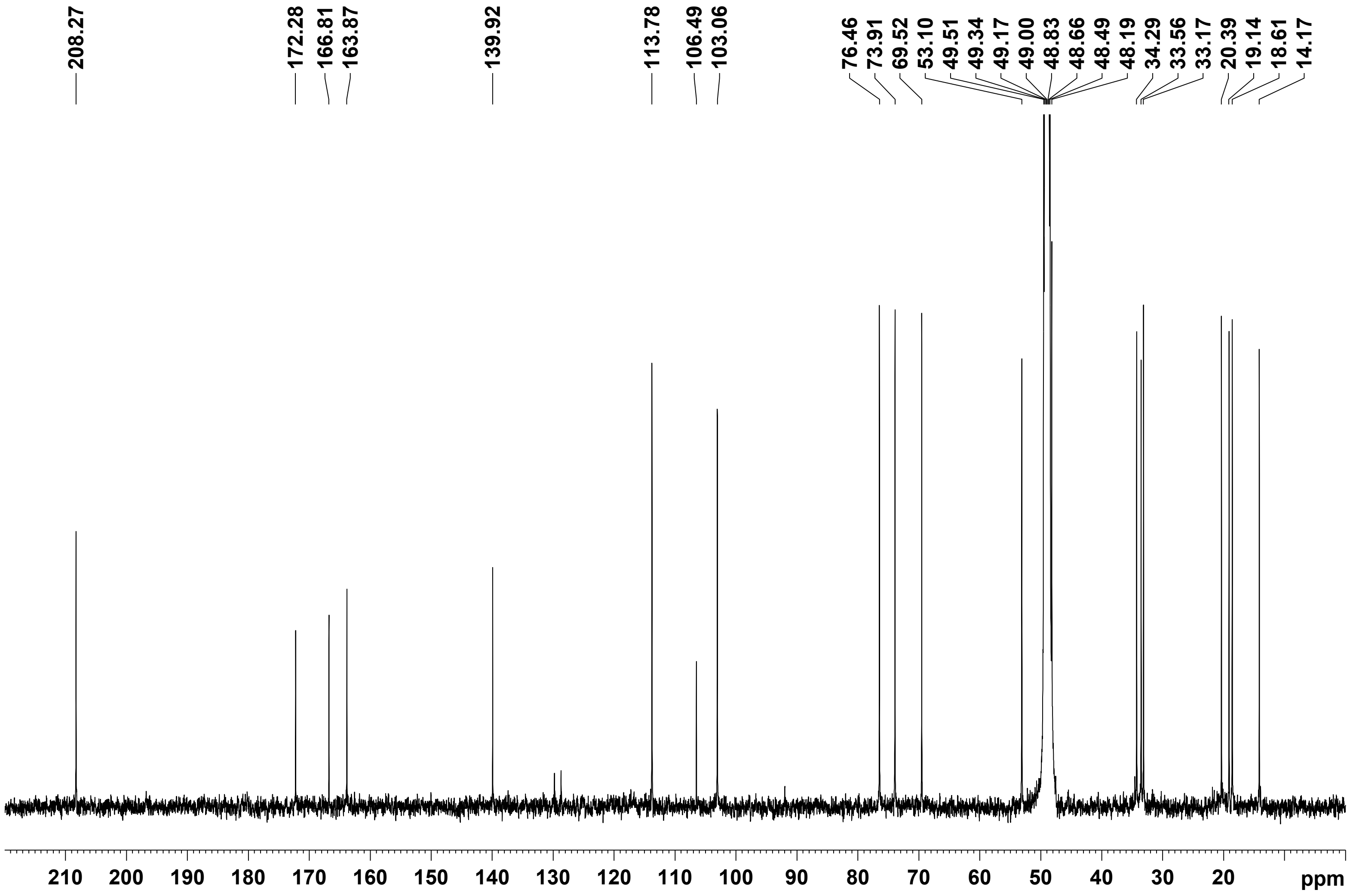

S61. ^{13}C NMR (125 MHz, CD_3OD) spectrum of compound **12**.

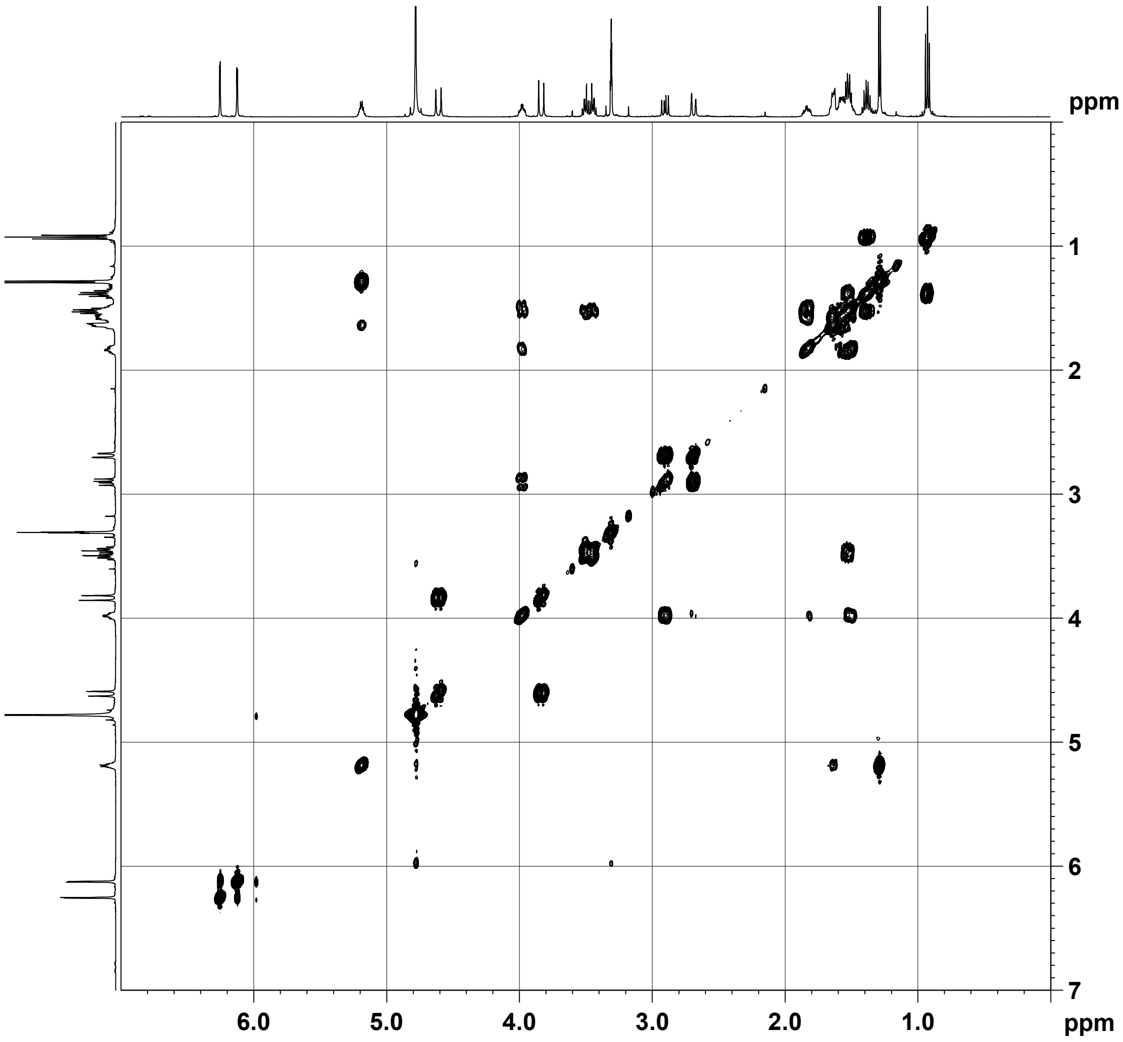

S62. ^{1}H-^{1}H COSY (500 MHz, CD_3OD) spectrum of compound **12**.

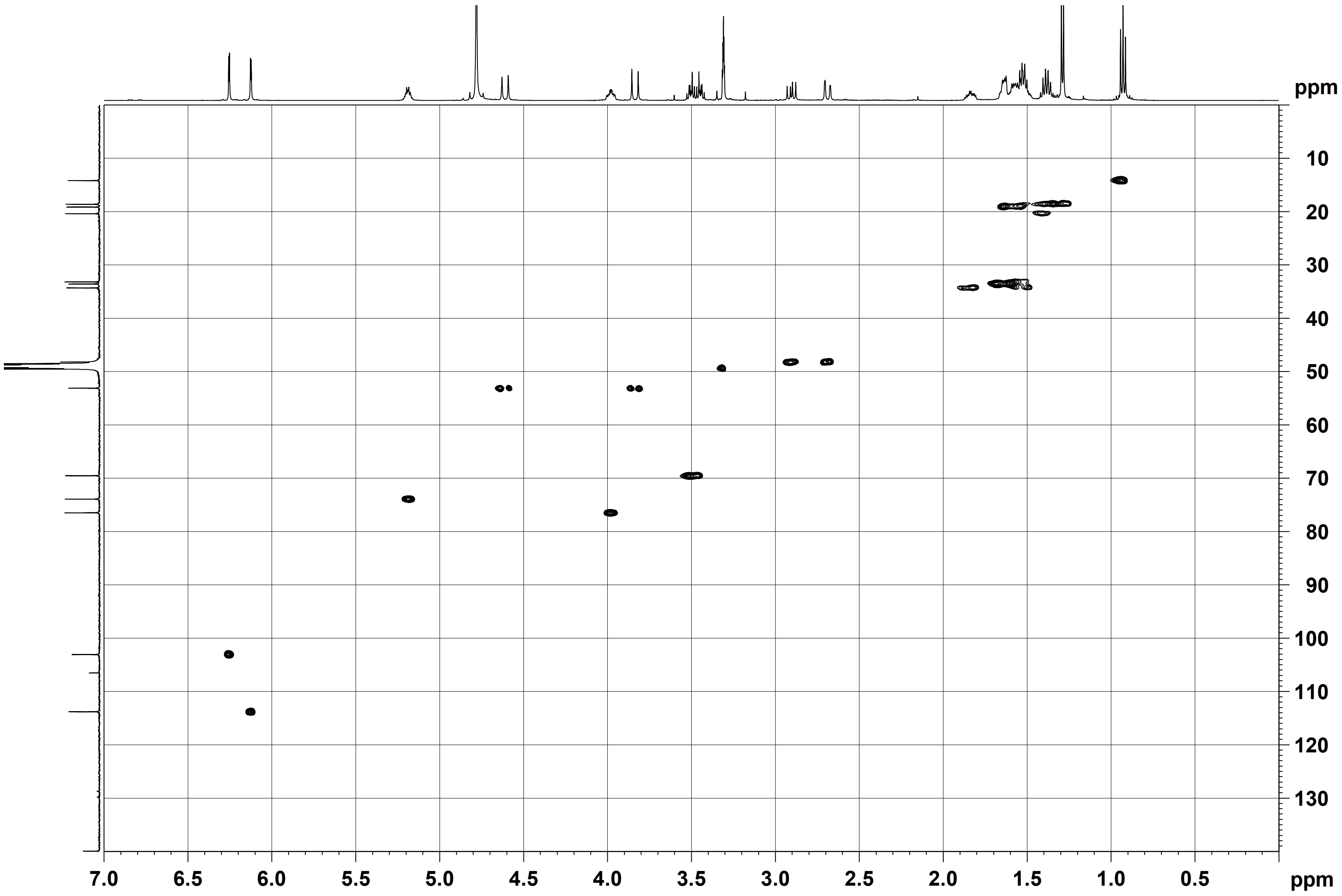

S63. HSQC (500 and 125 MHz, CD_3OD) spectrum of compound **12**.

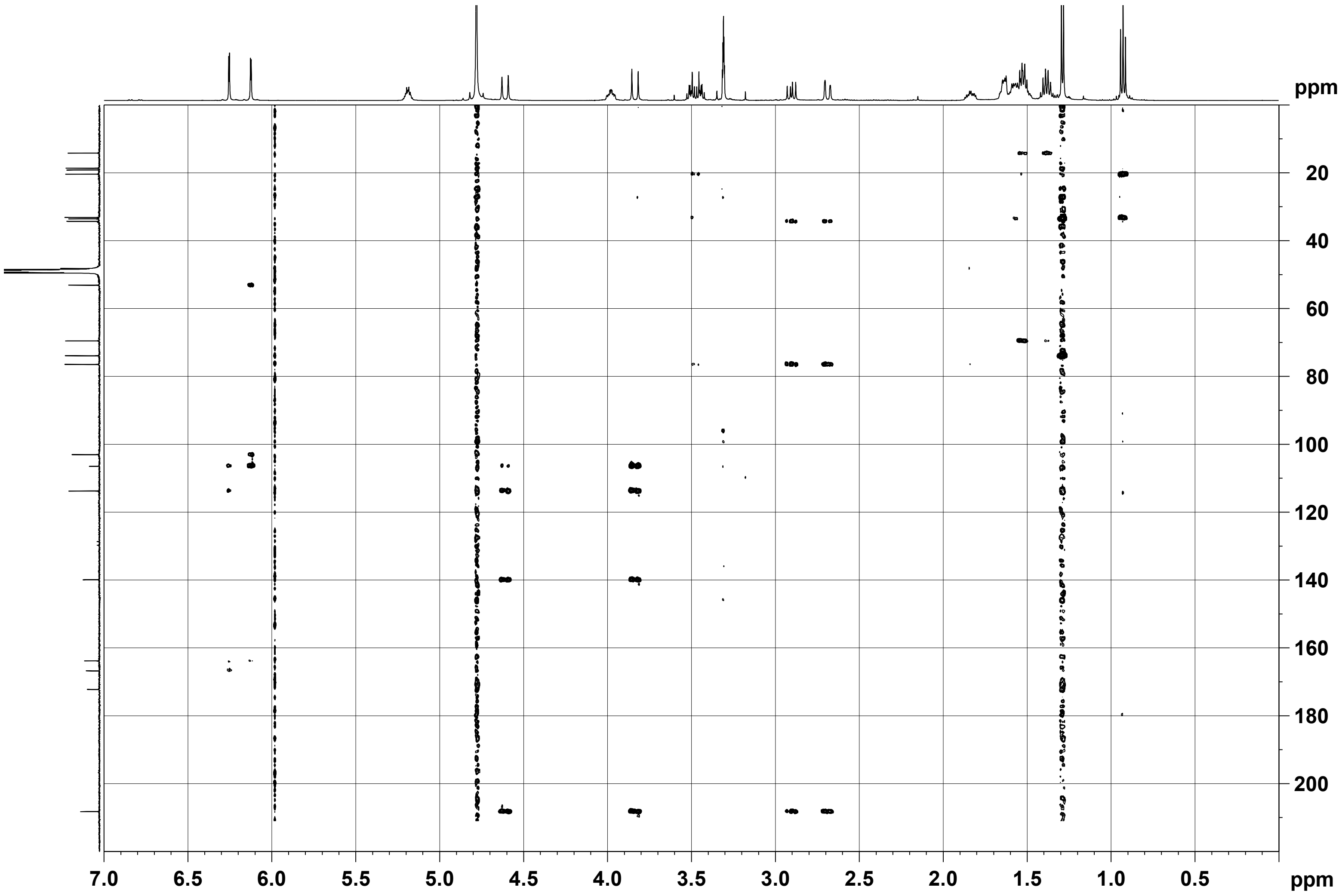

S64. HMBC (500 and 125 MHz, CD_3OD) spectrum of compound **12**.

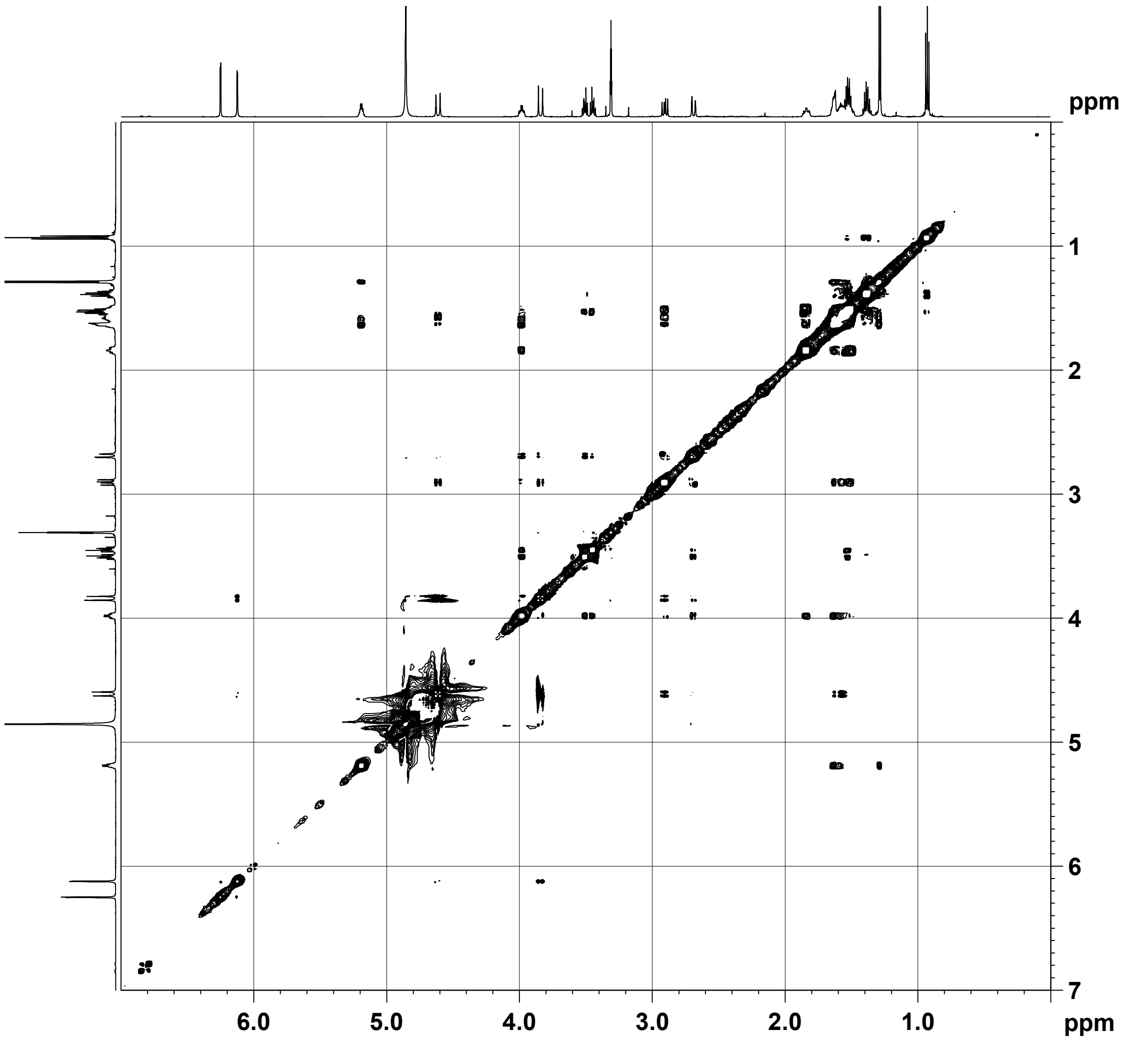

S65. ROESY (500 MHz, CD_3OD) spectrum of compound **12**

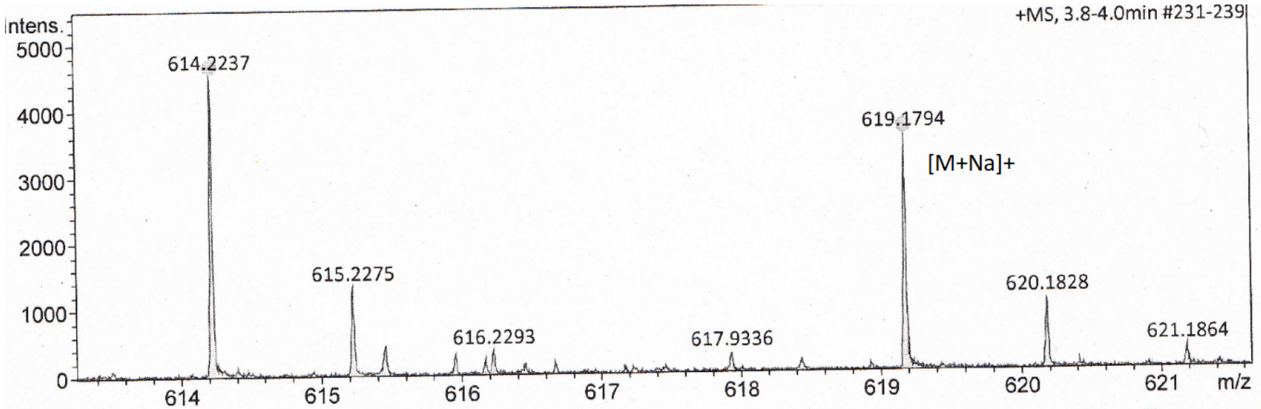

Meas. m/z	#	Ion Formula	m/z	err [ppm]	mSigma	# mSigma	Score	rdb	e⁻ Conf	N-Rule
614.2237	1	C31H36NO12	614.2232	-0.9	27.0	1	100.00	14.5	even	ok
	2	C32H32N5O8	614.2245	1.3	38.3	2	66.21	19.5	even	ok
	3	C29H24N15O2	614.2232	-0.9	38.6	3	74.28	25.5	even	ok
	4	C44H28N3O	614.2227	-1.7	99.7	4	5.94	32.5	even	ok
619.1794	1	C31H32NaO12	619.1786	-1.3	23.6	1	100.00	15.5	even	ok
	[illegible]	[illegible]	[illegible]	[illegible]	[illegible]	[illegible]	[illegible]	[illegible]	[illegible]	[illegible]

S66. HRESIMS spectrum of compound **13**.

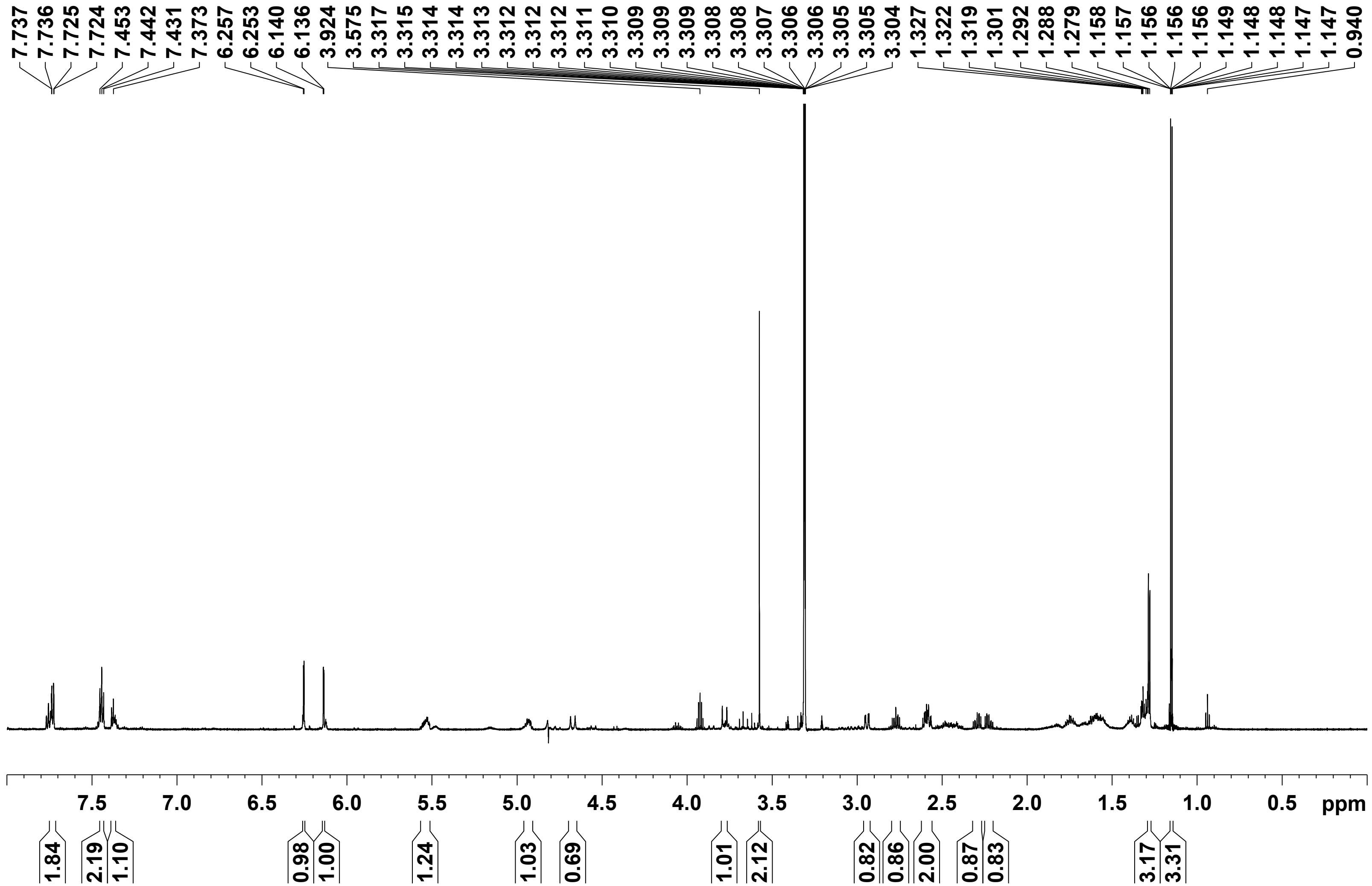

S67. ^{1}H NMR (700 MHz, CD_3OD) spectrum of compound **13**.

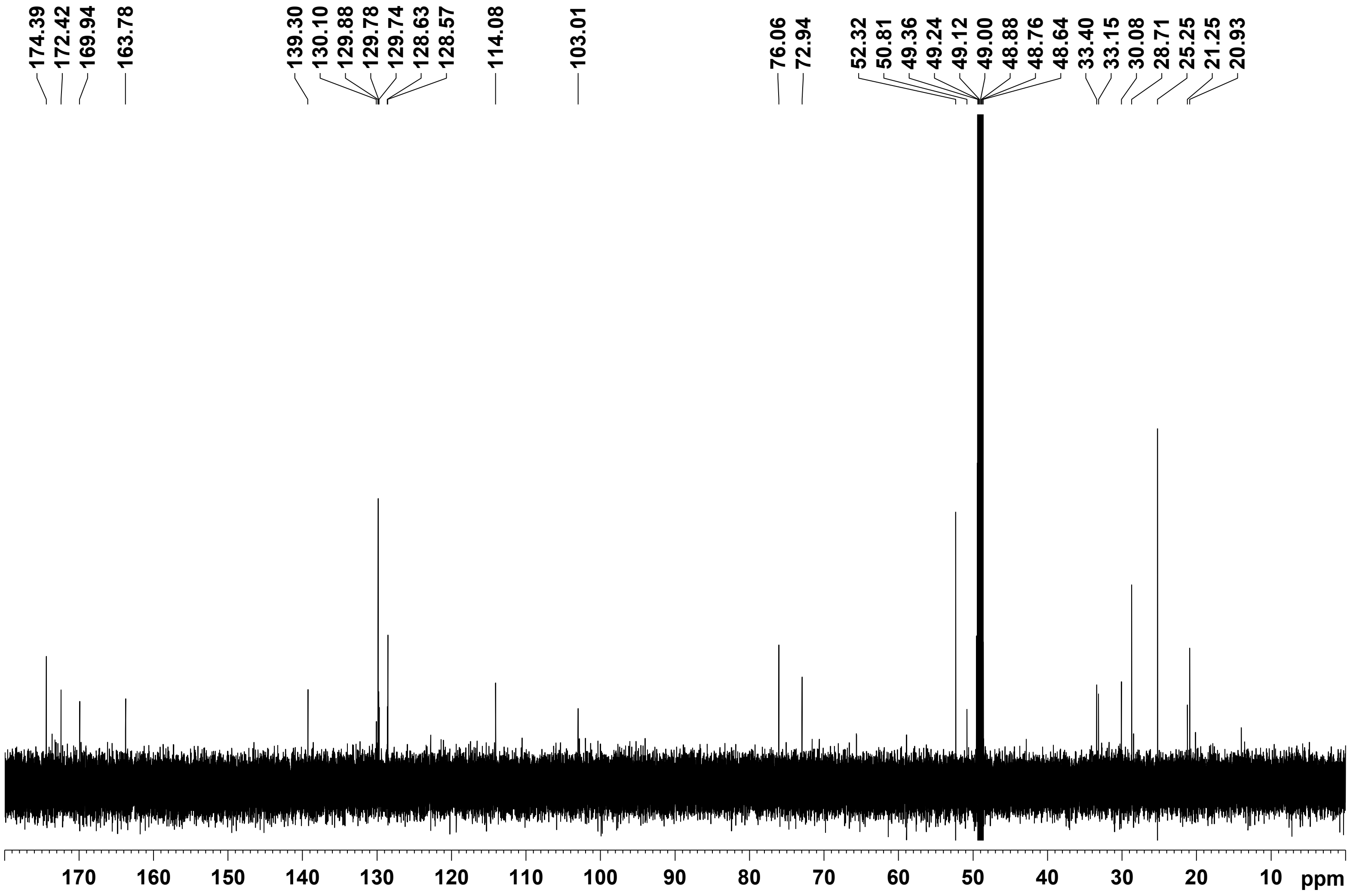

S68. ^{13}C NMR (175 MHz, CD_3OD) spectrum of compound **13**.

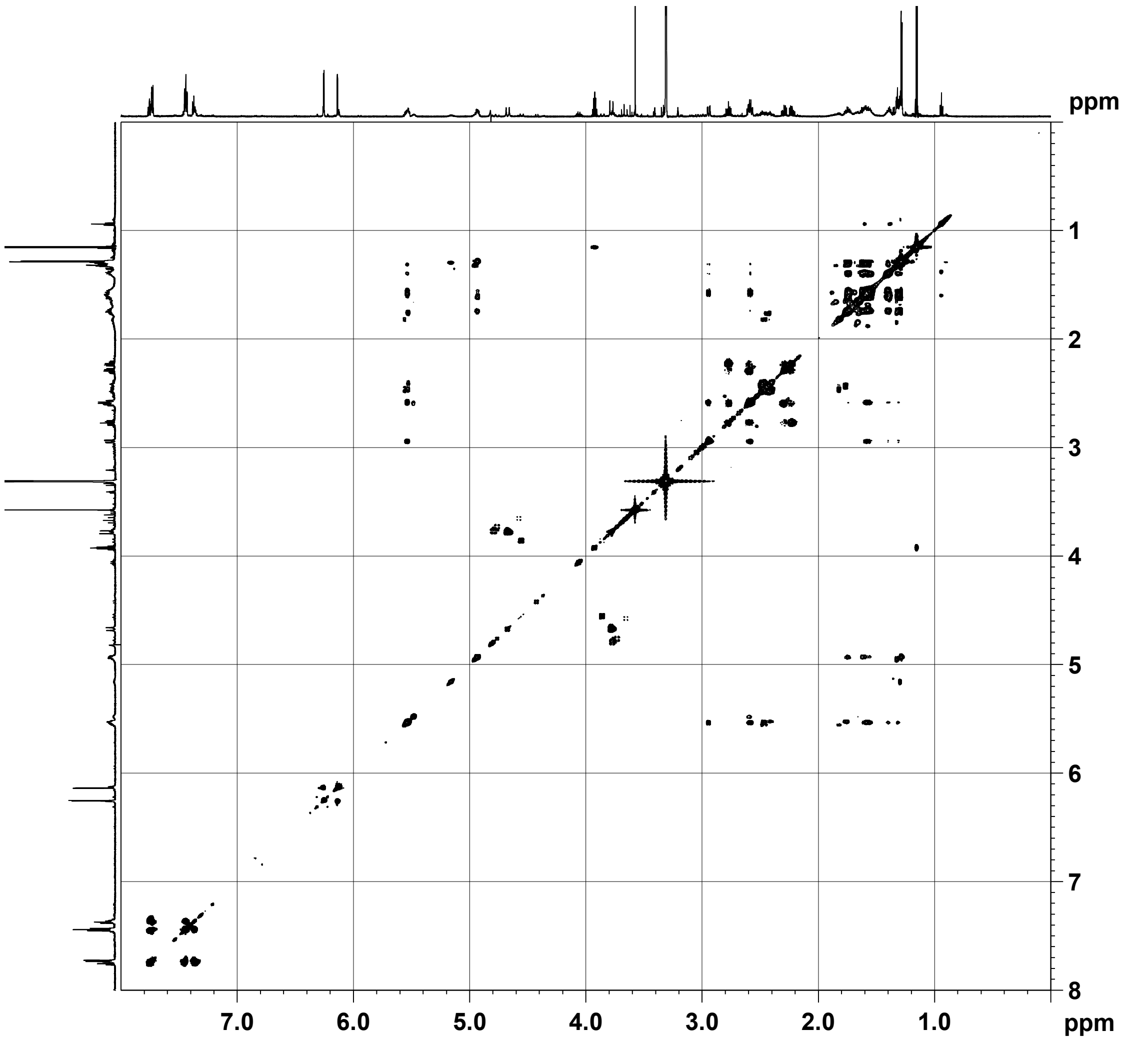

S69. ^{1}H-^{1}H TOCSY NMR (700 MHz, CD_3OD) spectrum of compound **13**.

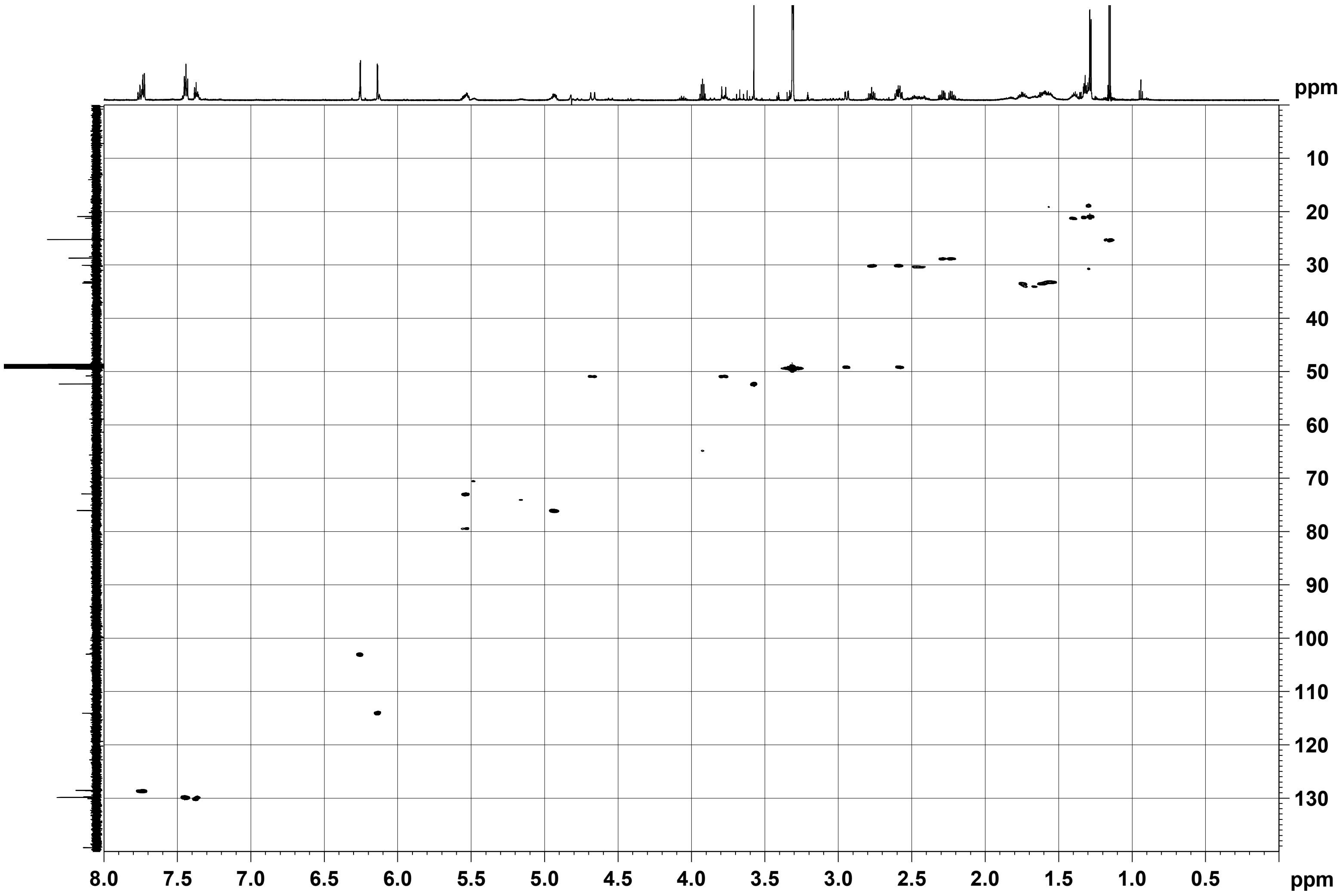

S70. HSQC (700 and 175 MHz, CD_3OD) spectrum of compound **13**.

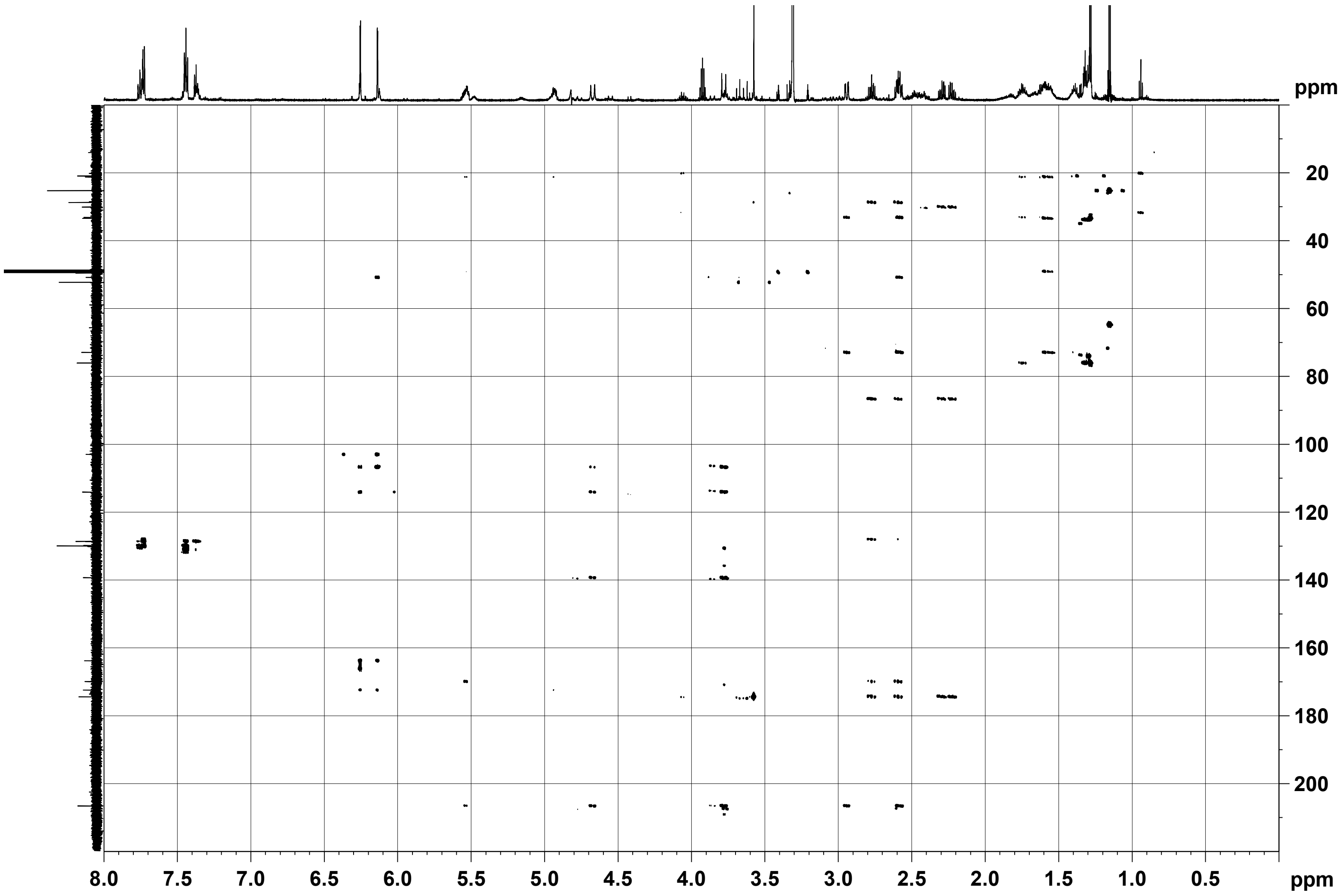

S71. HMBC (700 and 175 MHz, CD_3OD) spectrum of compound **13**.

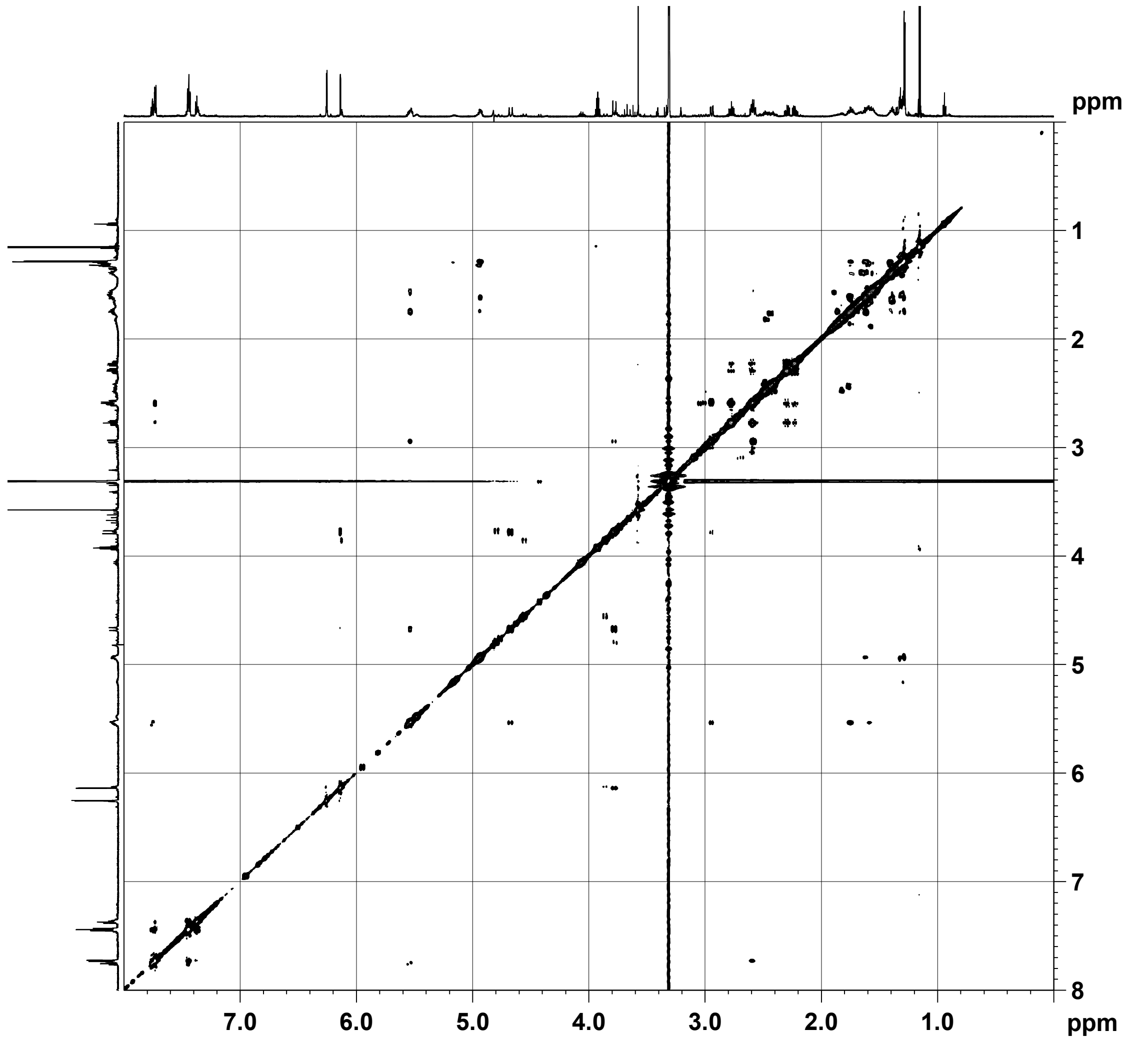

S72. ROESY (700 MHz, CD_3OD) spectrum of compound **13**.

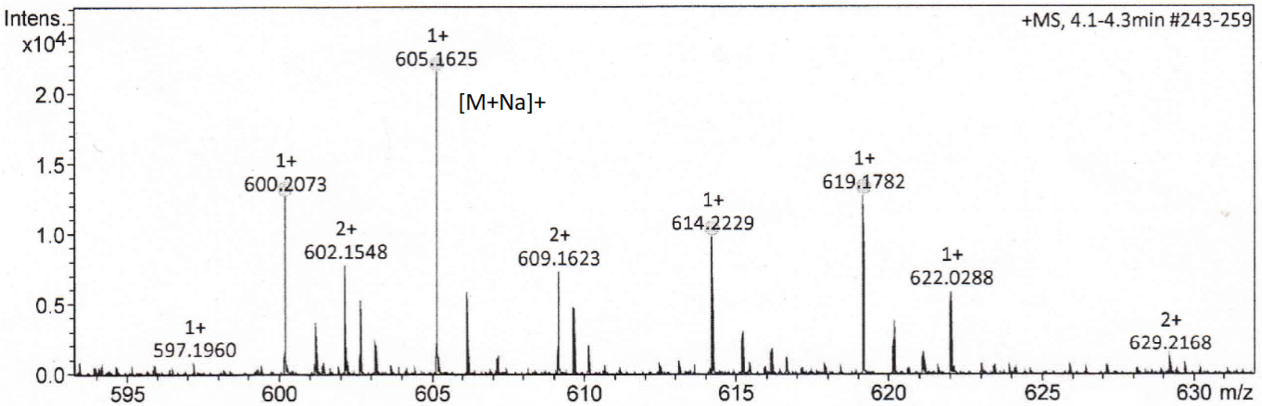

Meas. m/z	#	Ion Formula	m/z	err [ppm]	mSigma	# mSigma	Score	rdb	e⁻ Conf	N-Rule
600.2073	1	$C_{30}H_{34}NO_{12}$	600.2076	0.4	22.1	1	100.00	14.5	even	ok
	2	$C_{27}H_{26}N_{11}O_6$	600.2062	-1.8	23.1	2	62.59	20.5	even	ok
	3	$C_{28}H_{22}N_{15}O_2$	600.2075	0.4	34.7	3	74.53	25.5	even	ok
	4	$C_{43}H_{26}N_3O$	600.2070	-0.5	95.3	4	8.46	32.5	even	ok
605.1625	1	$C_{27}H_{29}N_2O_{14}$	605.1613	-1.9	19.8	1	77.84	14.5	even	ok
	2	$C_{25}H_{17}N_{16}O_4$	605.1613	-1.9	30.5	2	60.55	25.5	even	ok
	3	$C_{28}H_{25}N_6O_{10}$	605.1627	0.3	30.6	3	100.00	19.5	even	ok
	4	$C_{27}H_{22}N_{10}NaO_6$	605.1616	-1.5	31.1	4	70.12	21.5	even	ok
	5	$C_{30}H_{30}NaO_{12}$	605.1629	0.8	31.7	5	85.94	15.5	even	ok

S73. HRESIMS spectrum of compound **14**.

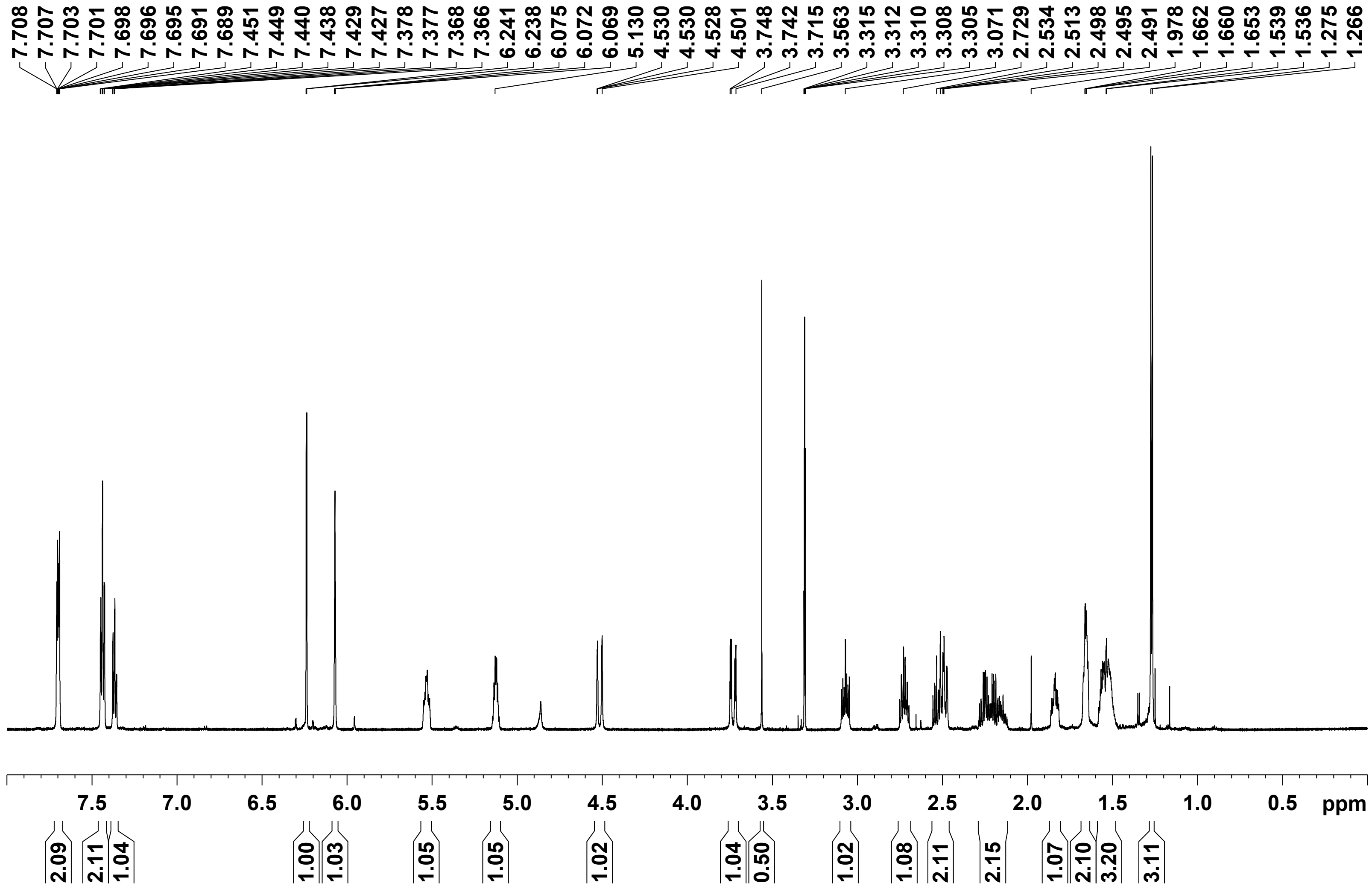

S74. ^{1}H NMR (700 MHz, CD_3OD) spectrum of compound **14**.

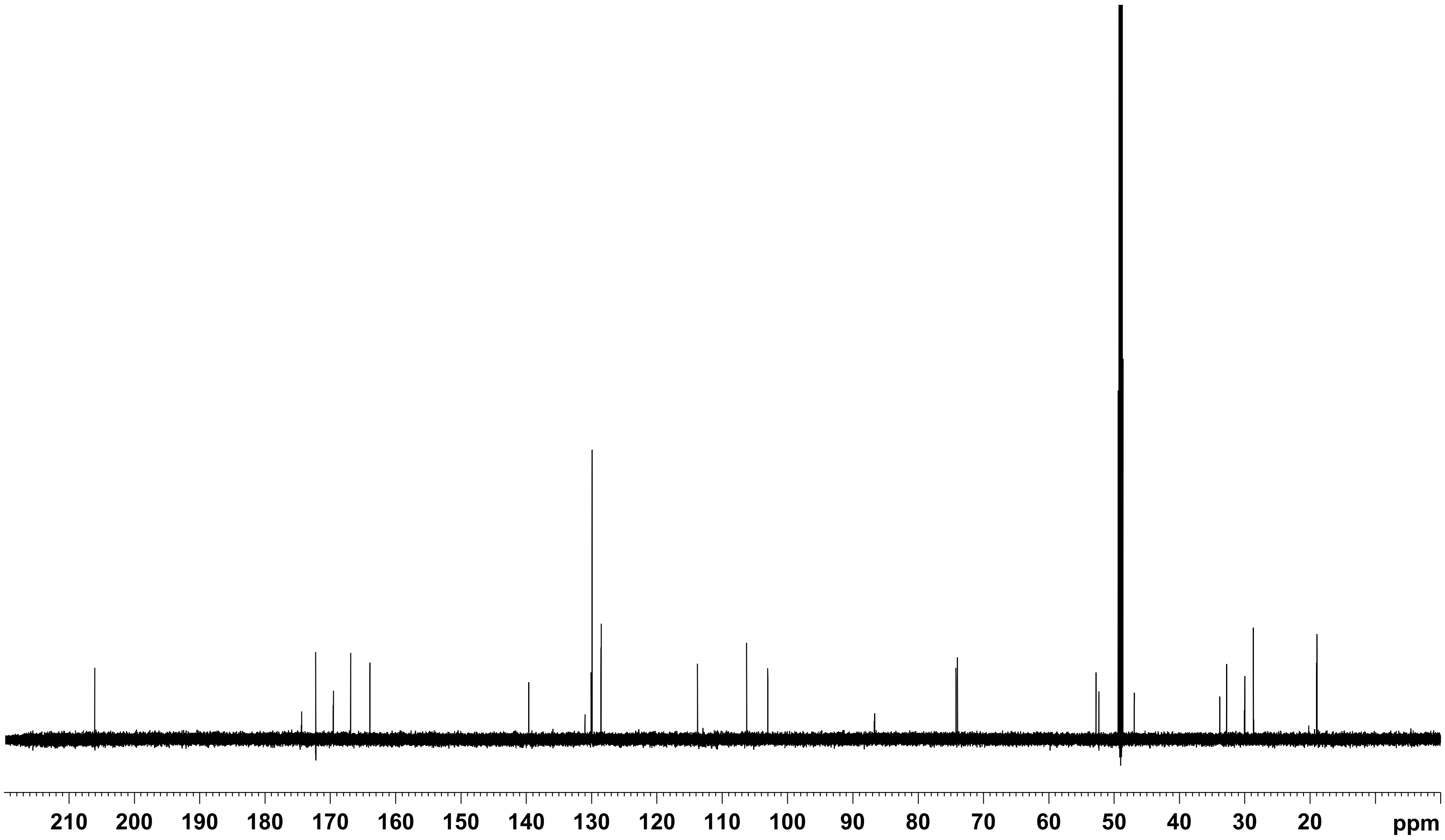

S75. ^{13}C NMR (175 MHz, CD_3OD) spectrum of compound **14**.

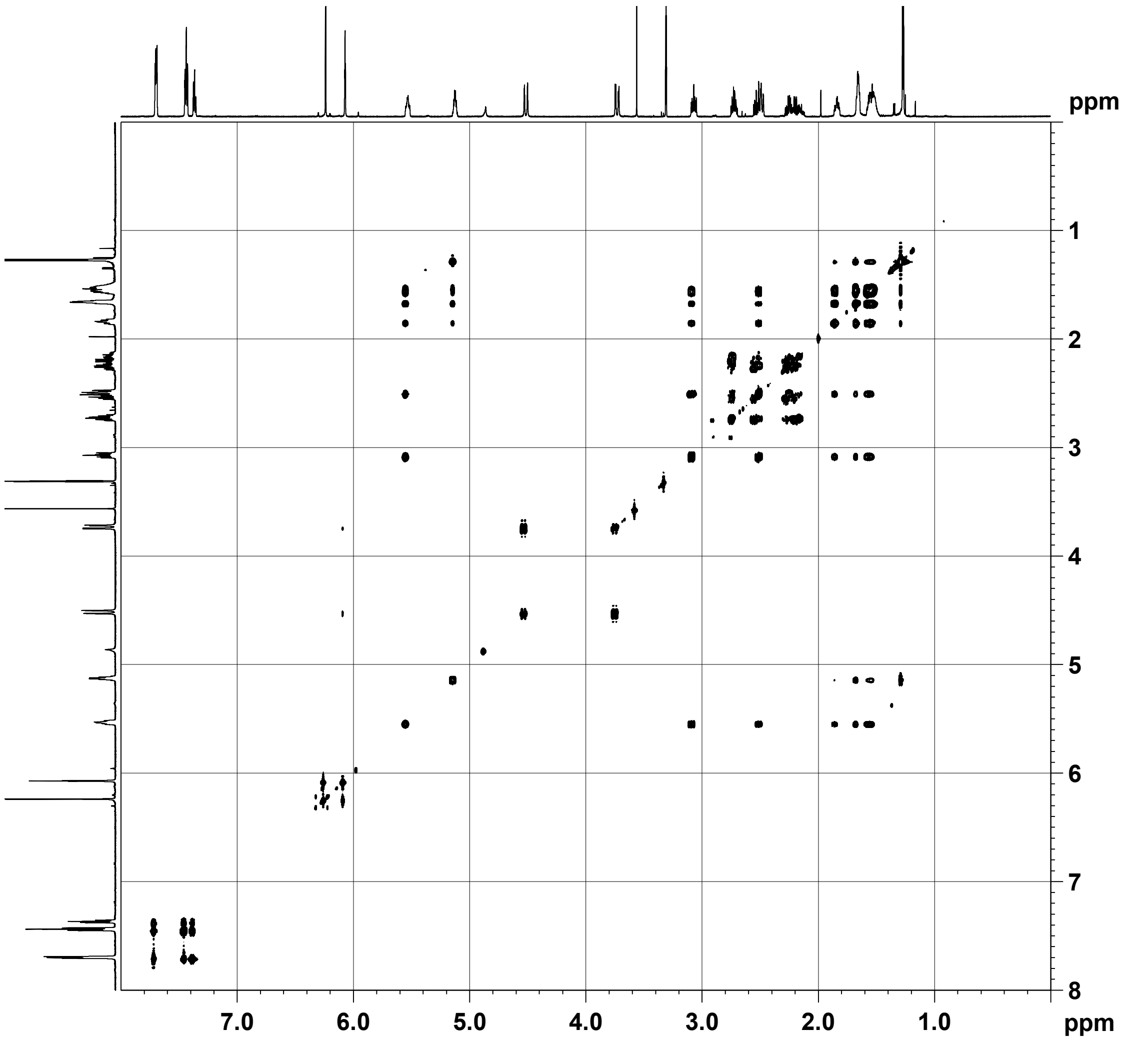

S76. ^{1}H-^{1}HTOCSY (700 MHz, CD_3OD) spectrum of compound **14**.

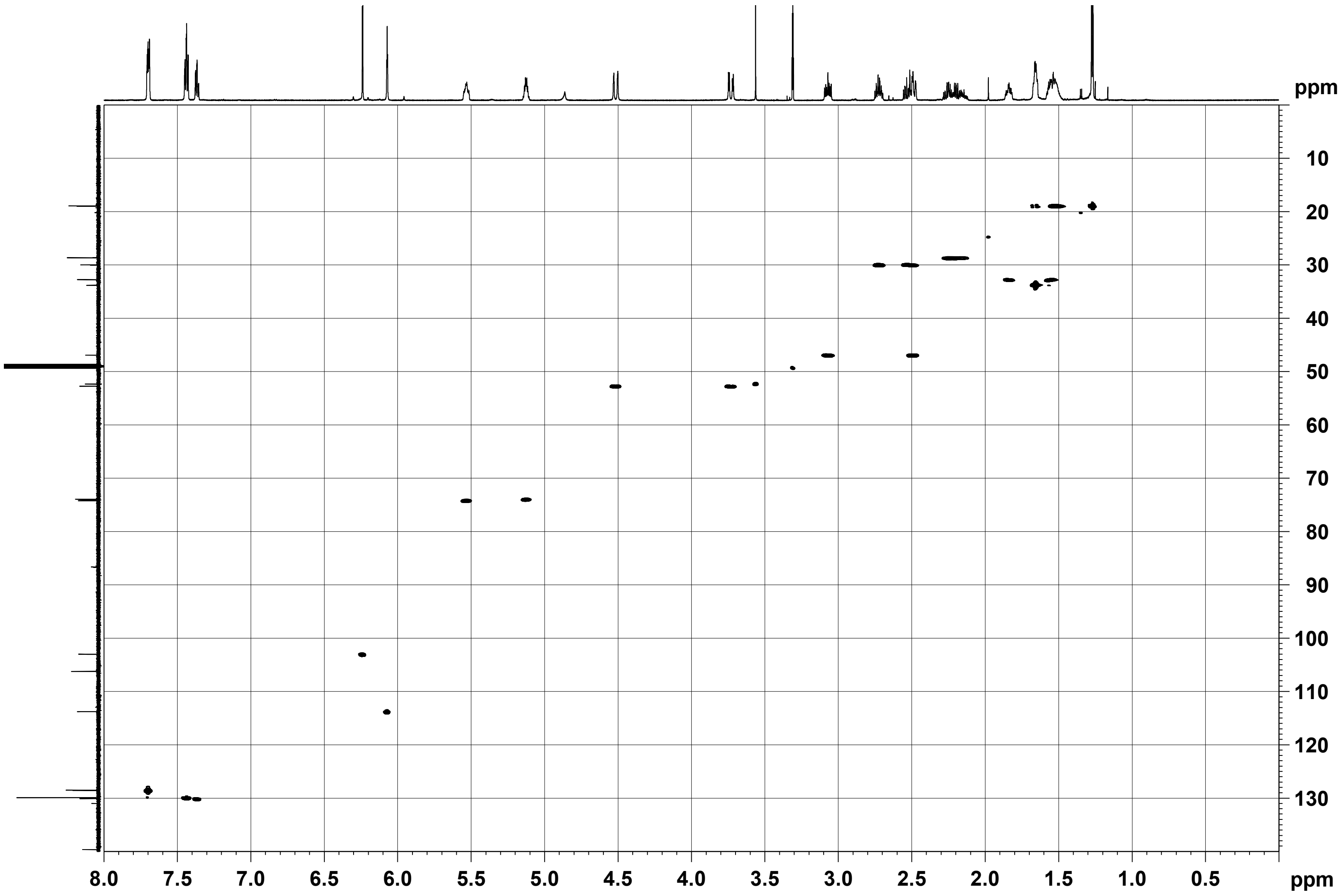

S77. HSQC (700 and 175 MHz, CD_3OD) spectrum of compound **14**.

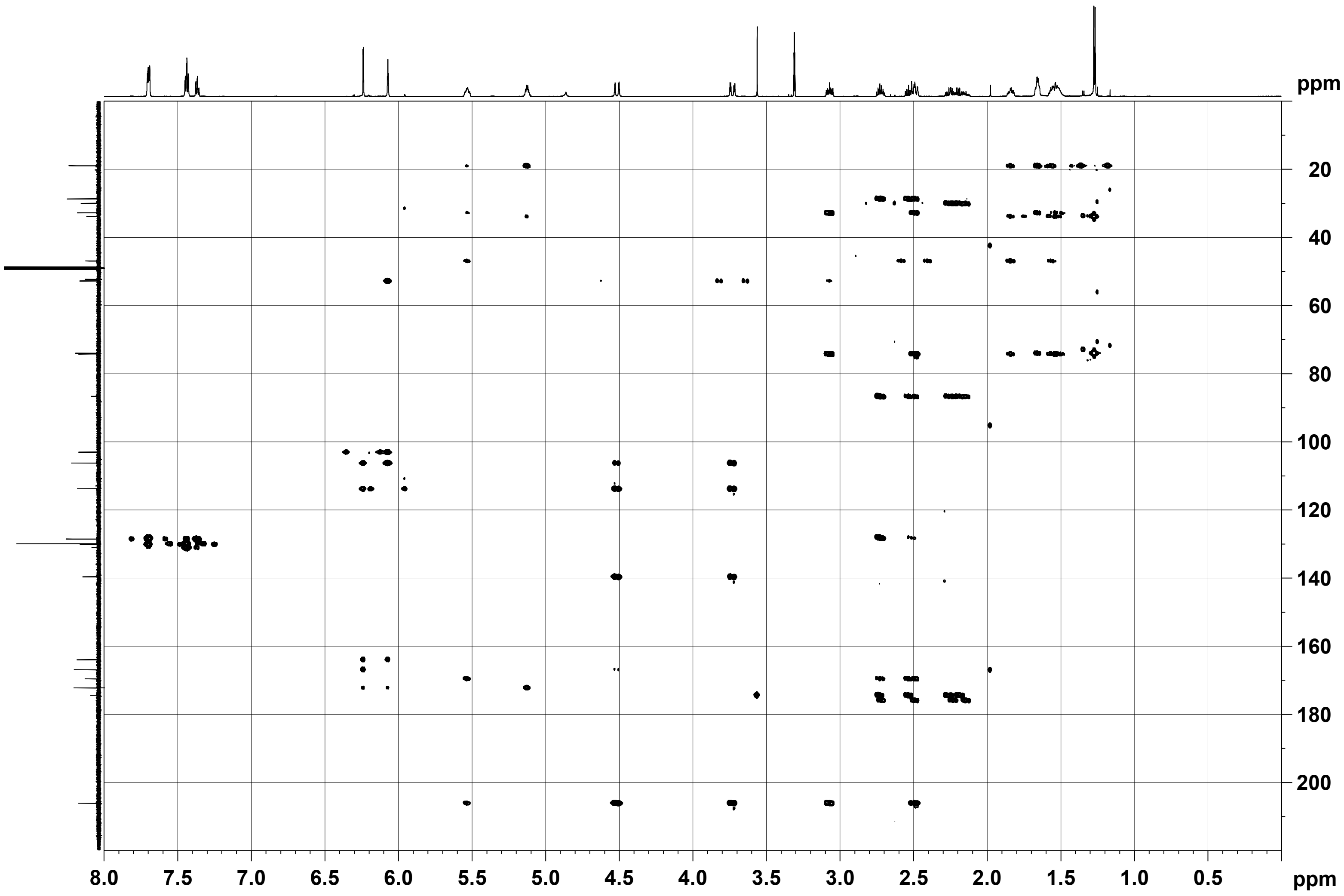

S78. HMBC (700 and 175 MHz, CD_3OD) spectrum of compound **14**.

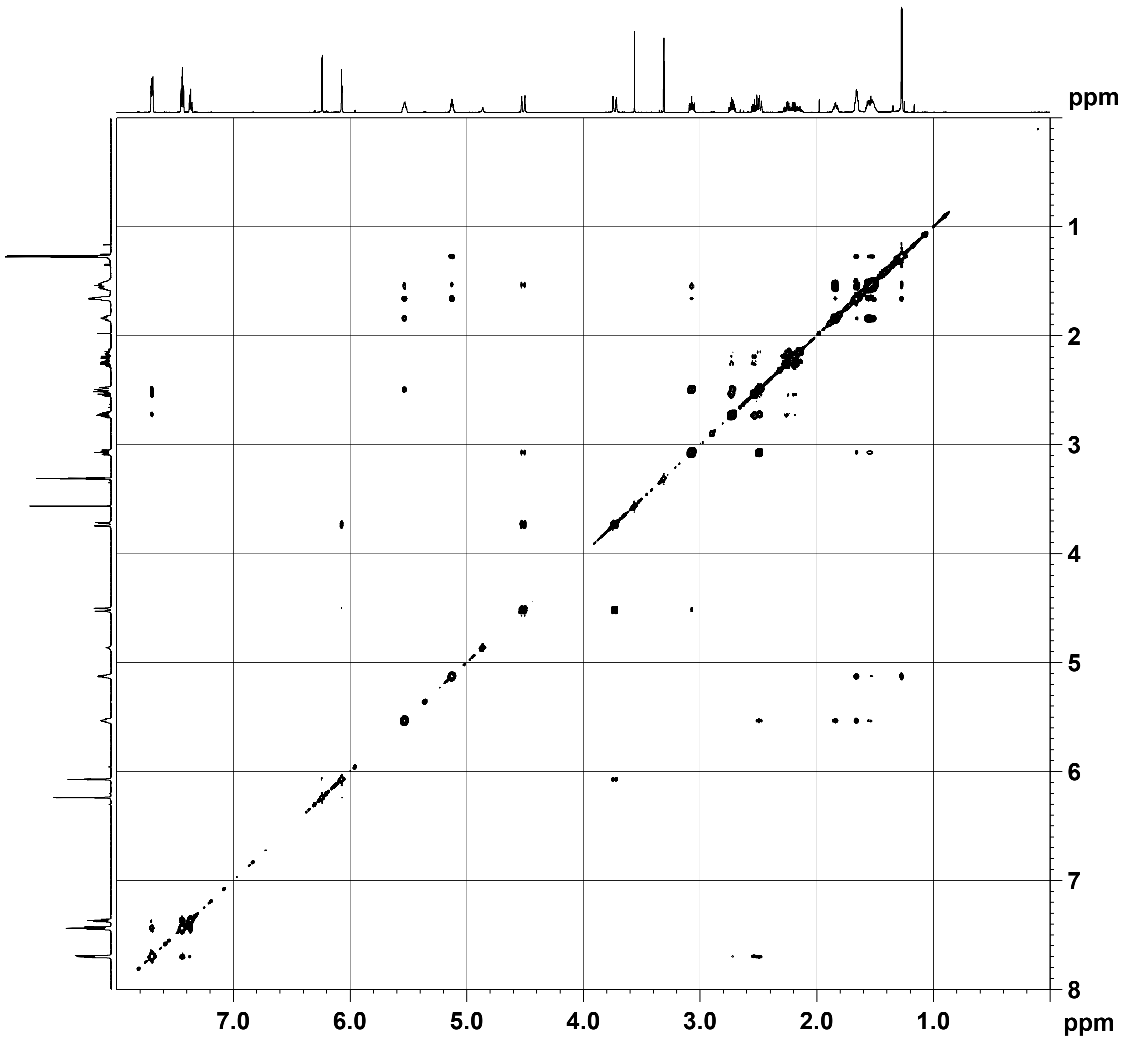

S79. ROESY (700 MHz, CD_3OD) spectrum of compound **14**.

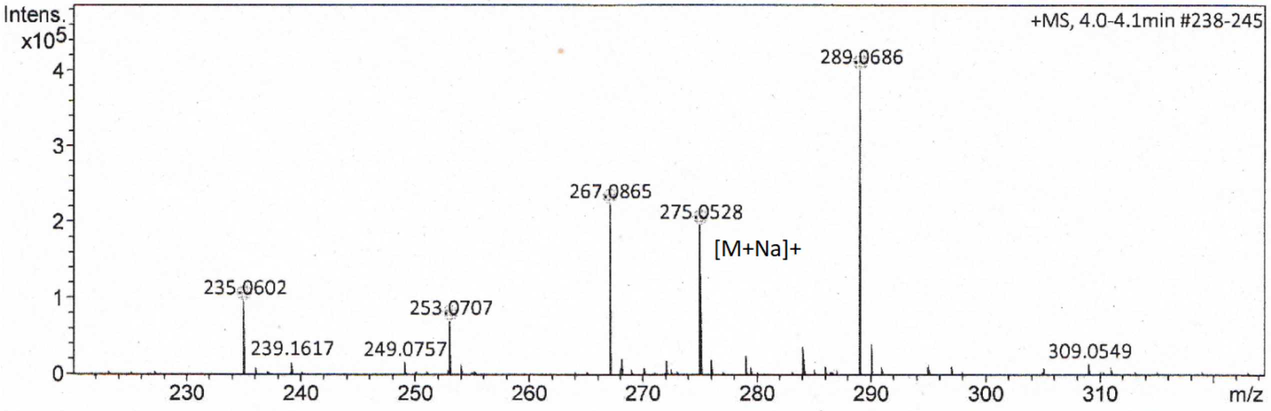

Meas. m/z	#	Ion Formula	m/z	err [ppm]	mSigma	# mSigma	Score	rdb	e¯ Conf	N-Rule
235.0602	1	C12H11O5	235.0601	-0.2	20.8	1	100.00	7.5	even	ok
	2	C11H8N4NaO	235.0590	-4.8	21.8	2	57.43	9.5	even	ok
	3	C13H7N4O	235.0614	5.4	34.1	3	39.02	12.5	even	ok
253.0707	1	C12H13O6	253.0707	-0.2	10.9	1	100.00	6.5	even	ok
	2	C9H5N10	253.0693	-5.5	12.6	2	47.98	12.5	even	ok
	3	C13H9N4O2	253.0720	5.1	24.4	3	39.68	11.5	even	ok
267.0865	1	C13H15O6	267.0863	-0.8	28.2	1	100.00	6.5	even	ok
	2	C10H7N10	267.0850	-5.9	28.4	2	47.28	12.5	even	ok
	3	C12H12N4NaO2	267.0852	-4.8	28.9	3	56.01	8.5	even	ok
	4	C14H11N4O2	267.0877	4.2	41.4	4	44.75	11.5	even	ok
275.0528	1	C9H11N2O8	275.0510	-6.5	7.7	1	51.17	5.5	even	ok
	2	C10H7N6O4	275.0523	-1.7	19.6	2	89.93	10.5	even	ok
	3	C12H12NaO6	275.0526	-0.7	20.5	3	100.00	6.5	even	ok

S80. HRESIMS spectrum of compound **15**.

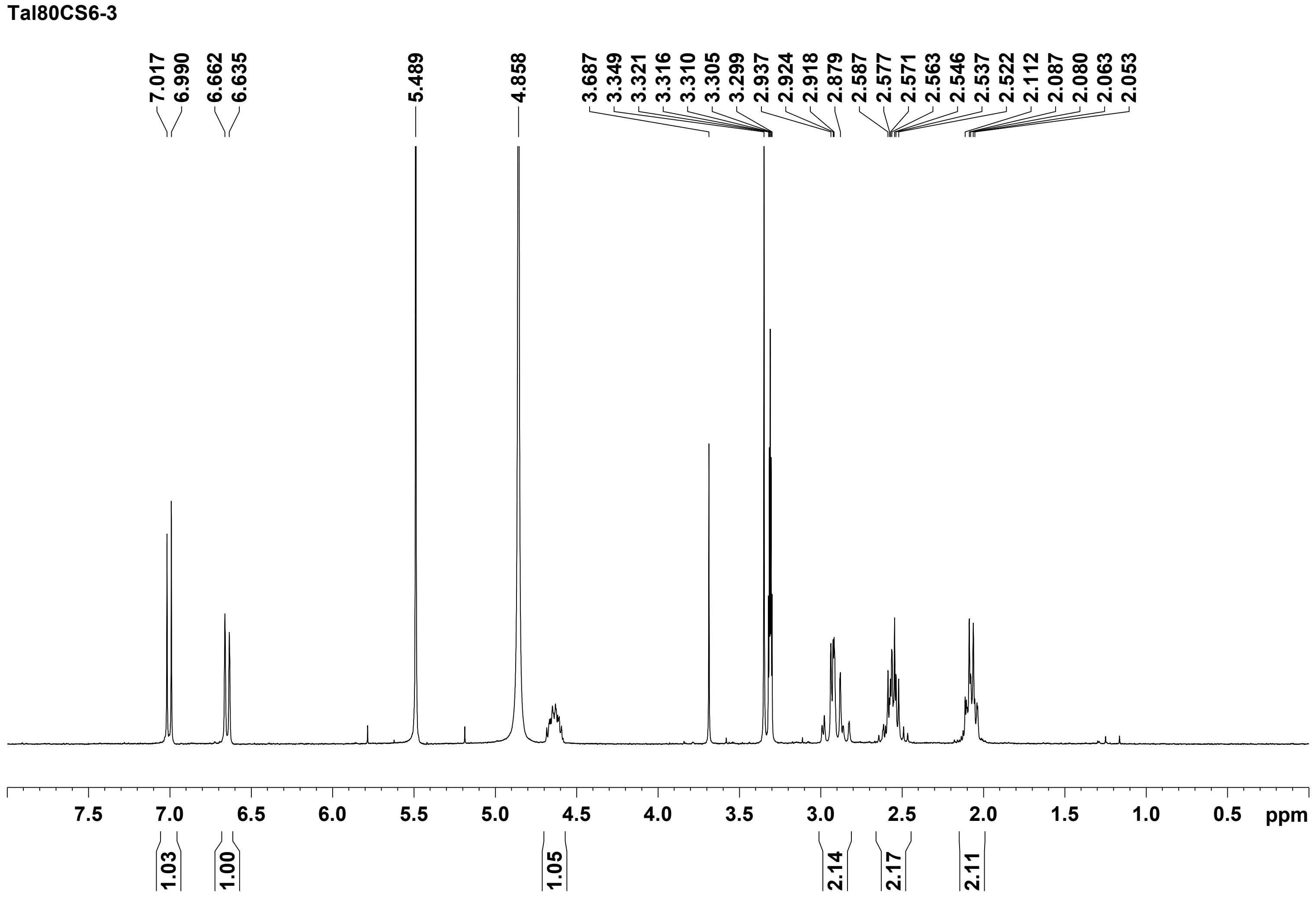

S81. ^{1}H NMR (300 MHz, CD_3OD) spectrum of compound **15**.

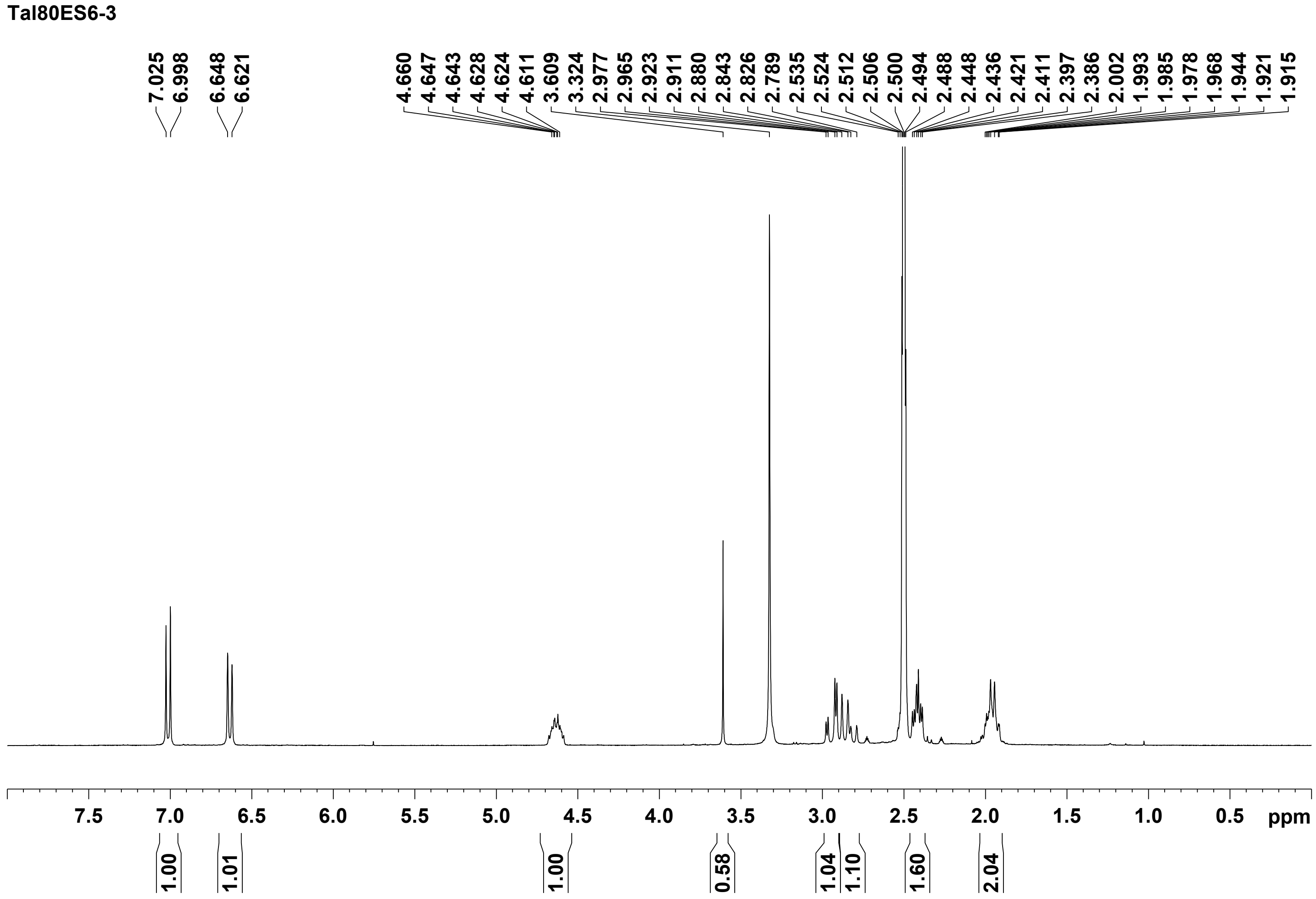

S82. ¹H NMR (300 MHz, DMSO-d_6) spectrum of compound **15**.

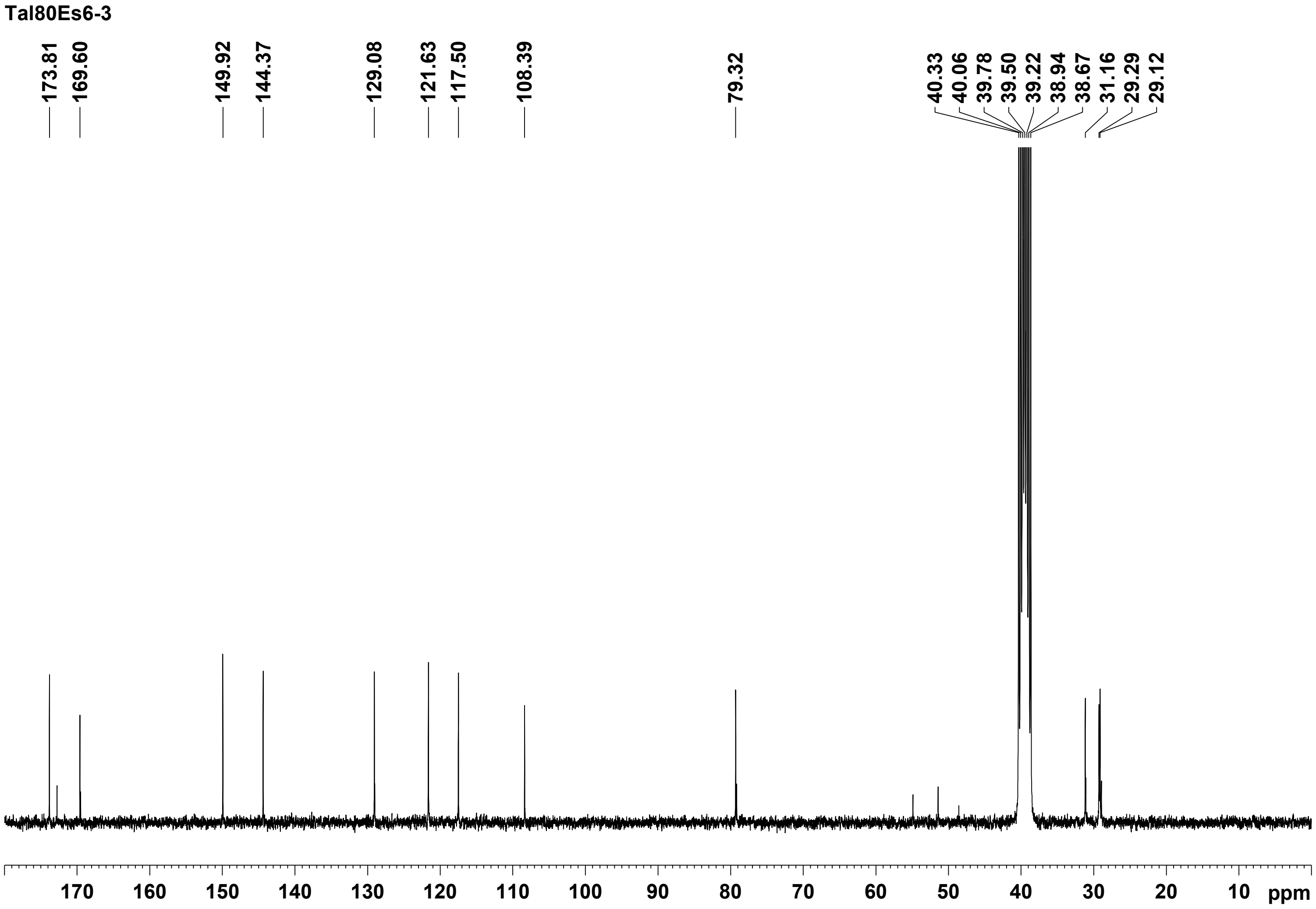

S83. ^{13}C NMR (75 MHz, DMSO-d_6) spectrum of compound **15**.

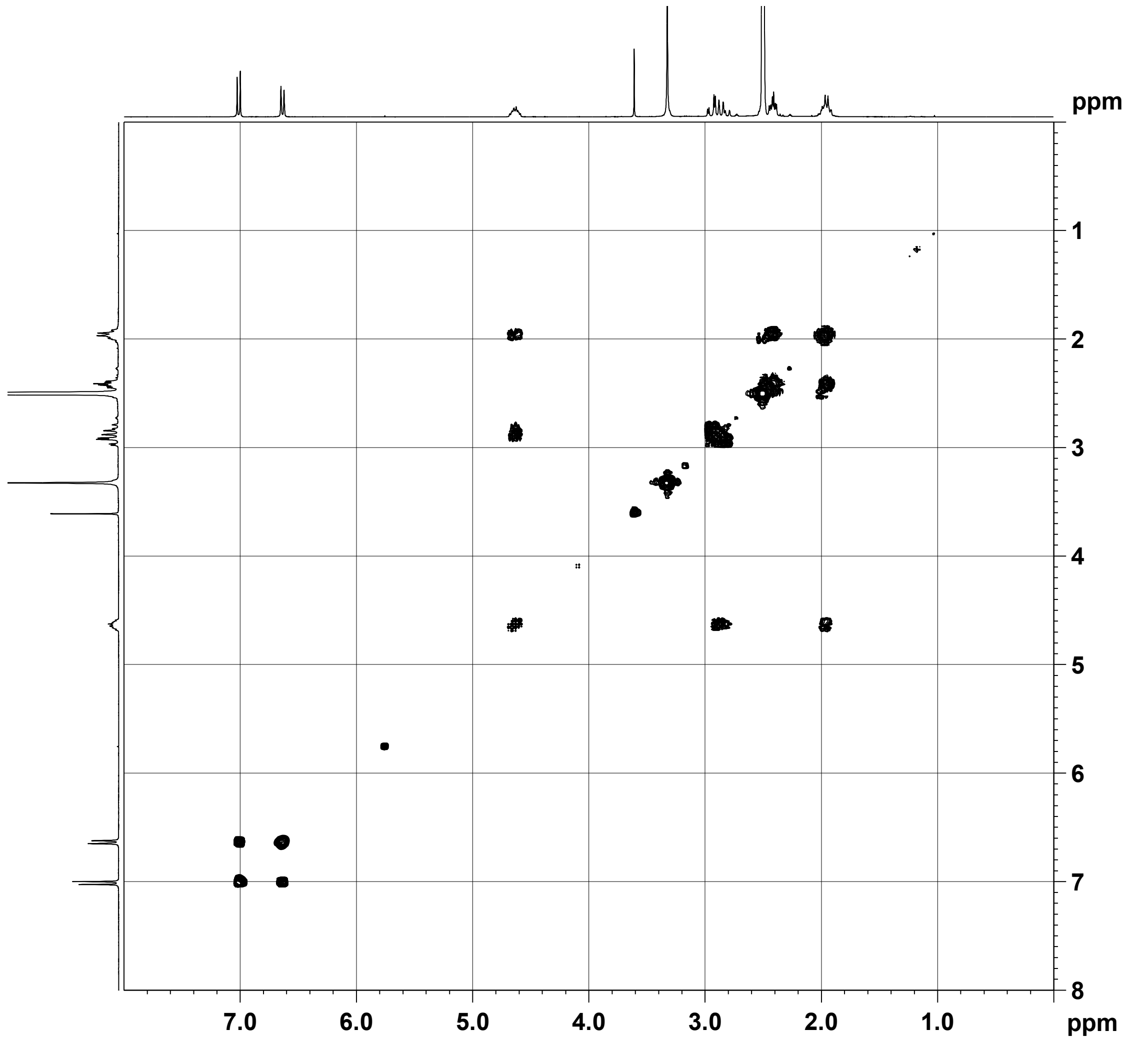

S84. ^{1}H-^{1}H COSY (300 MHz, DMSO-d_6) spectrum of compound **15**.

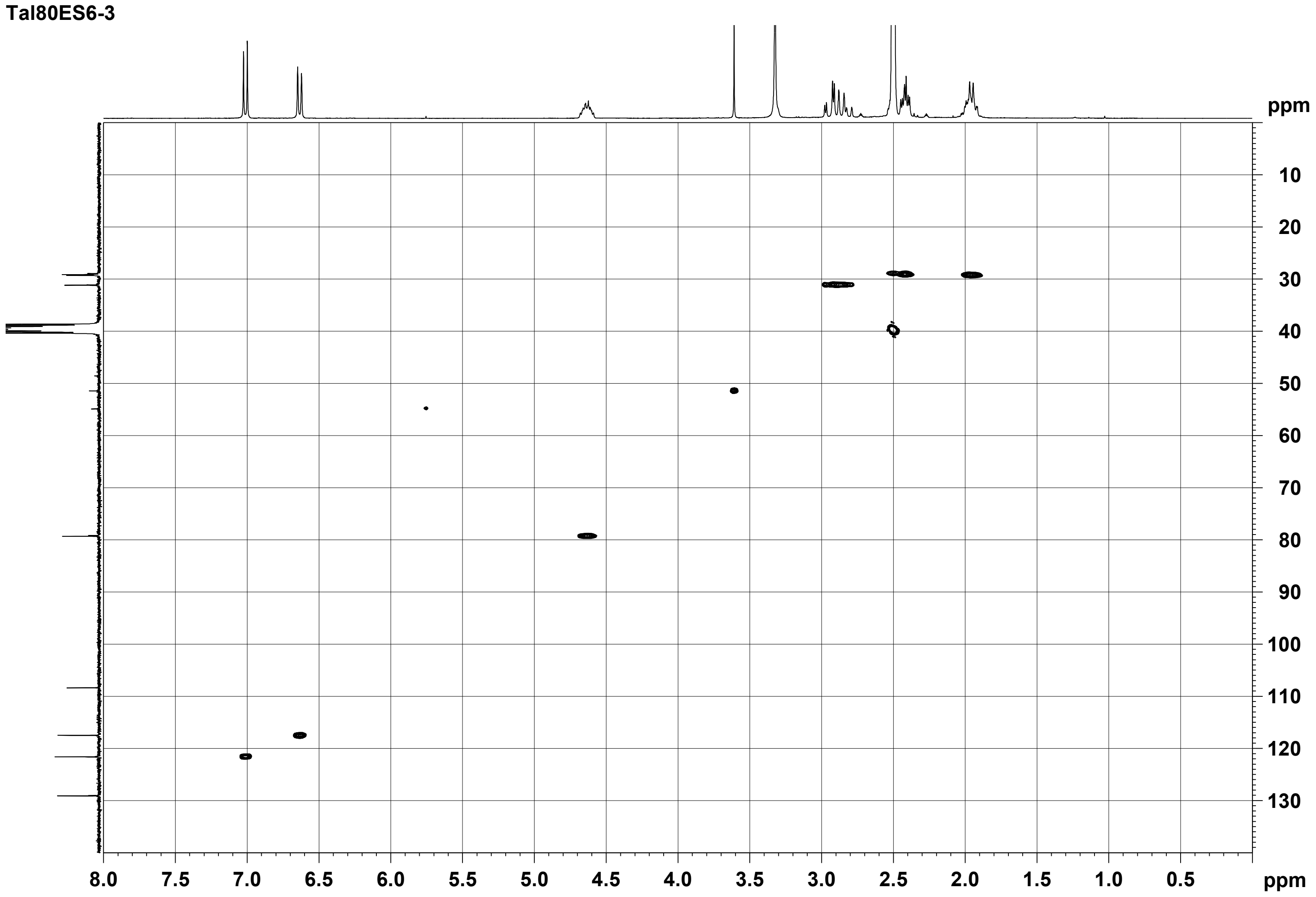

S85. HSQC (300 and 75 MHz, DMSO-d_6) spectrum of compound **15**.

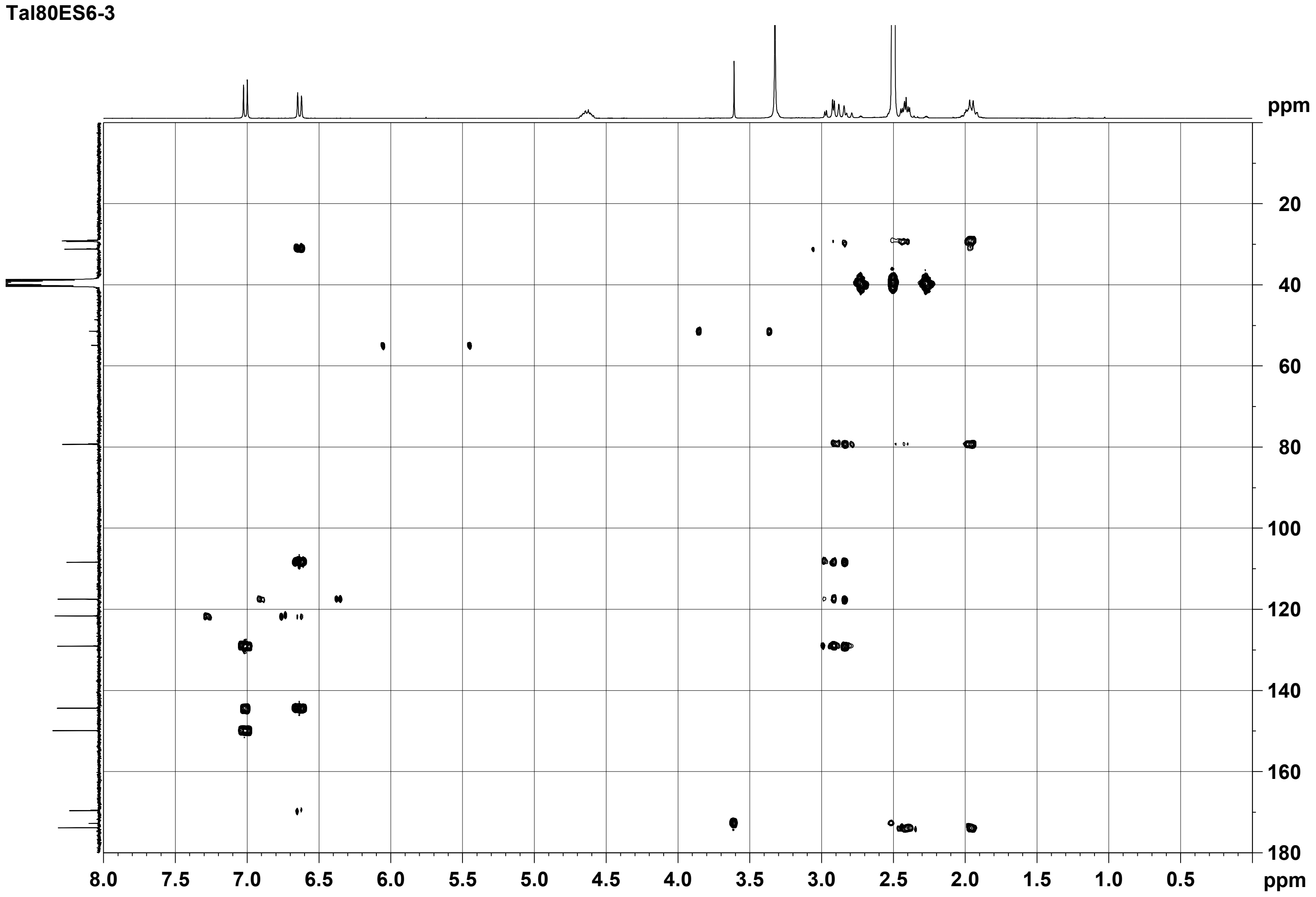

S86. HMBC (300 and 75 MHz, DMSO-d_6) spectrum of compound **15**.

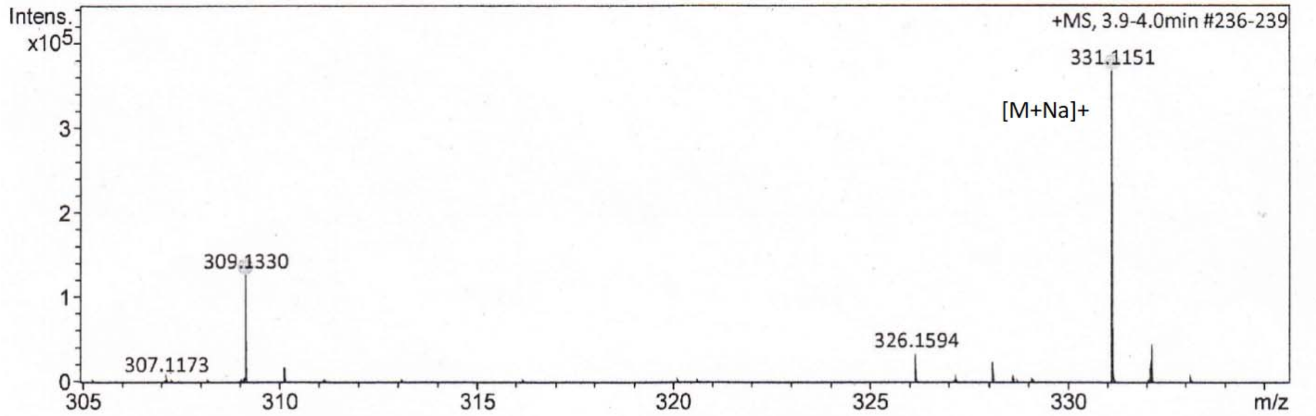

Meas. m/z	#	Ion Formula	m/z	err [ppm]	mSigma	# mSigma	Score	rdb	e⁻ Conf	N-Rule
309.1330	1	$C_{16}H_{21}O_6$	309.1333	0.7	24.6	1	100.00	6.5	even	ok
331.1151	1	$C_{16}H_{20}NaO_6$	331.1152	0.2	33.2	1	100.00	6.5	even	ok

S87. HRESIMS spectrum of compound **16**.

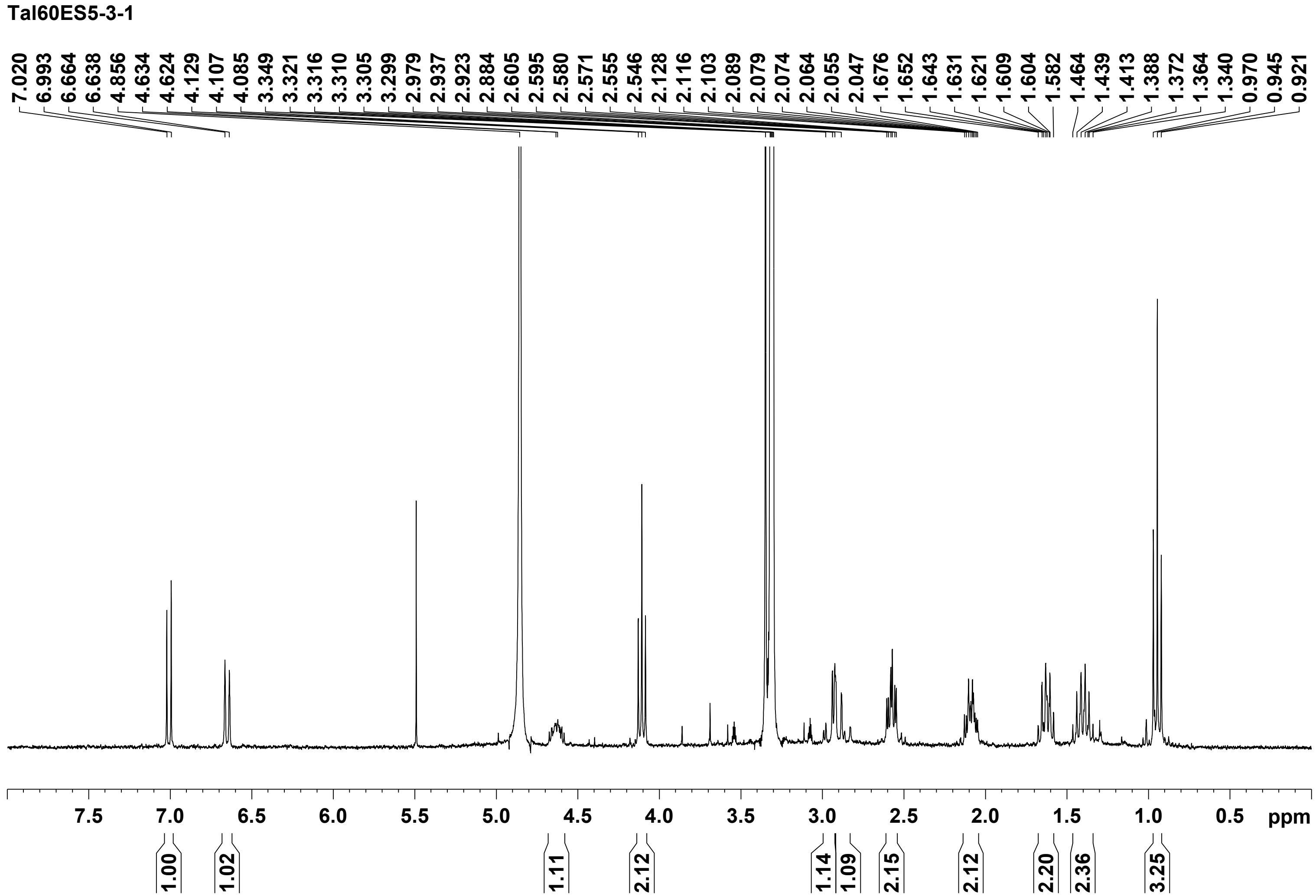

S88. ^{1}H NMR (300 MHz, CD_3OD) spectrum of compound **16**.

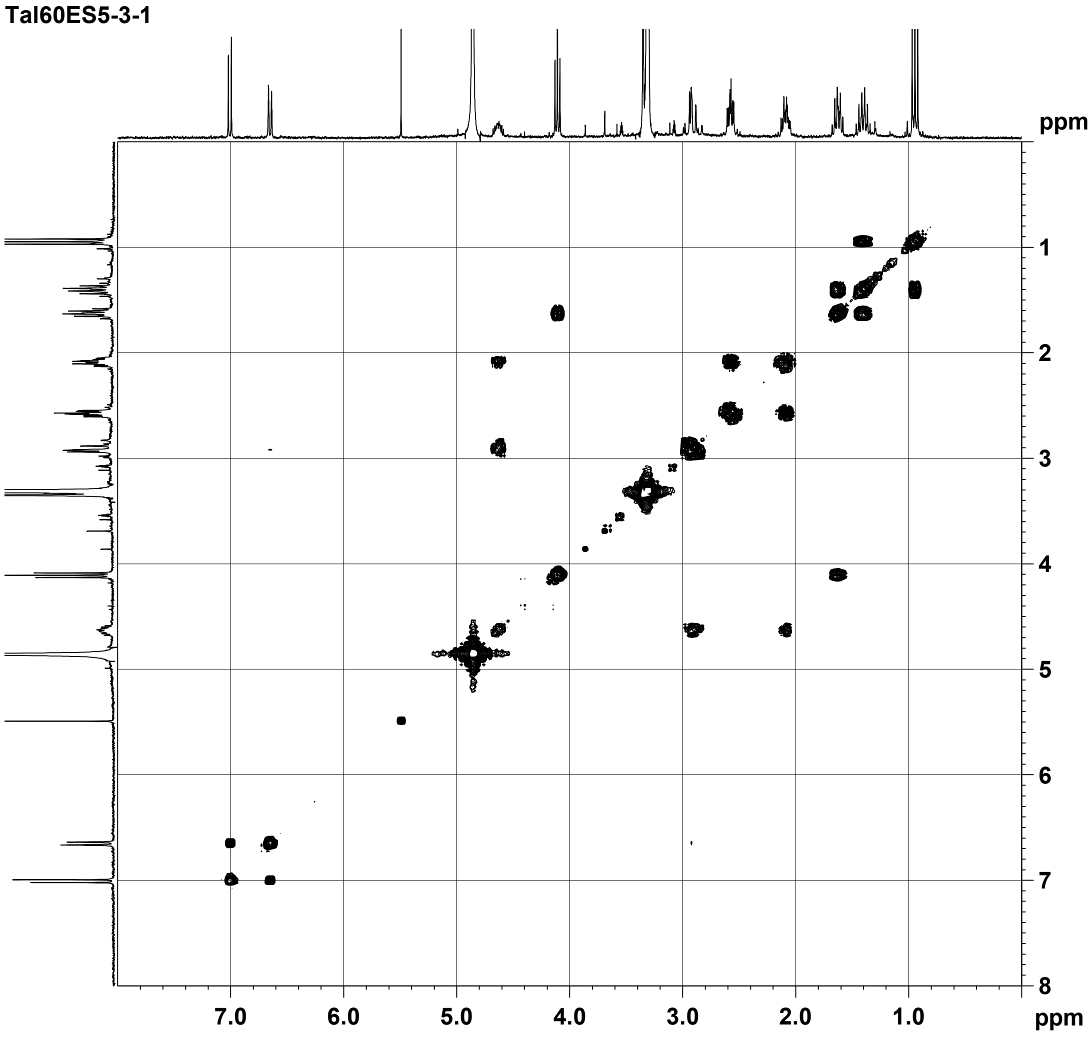

S89. ^{1}H-^{1}H COSY (300 MHz, CD_3OD) spectrum of compound **16**.

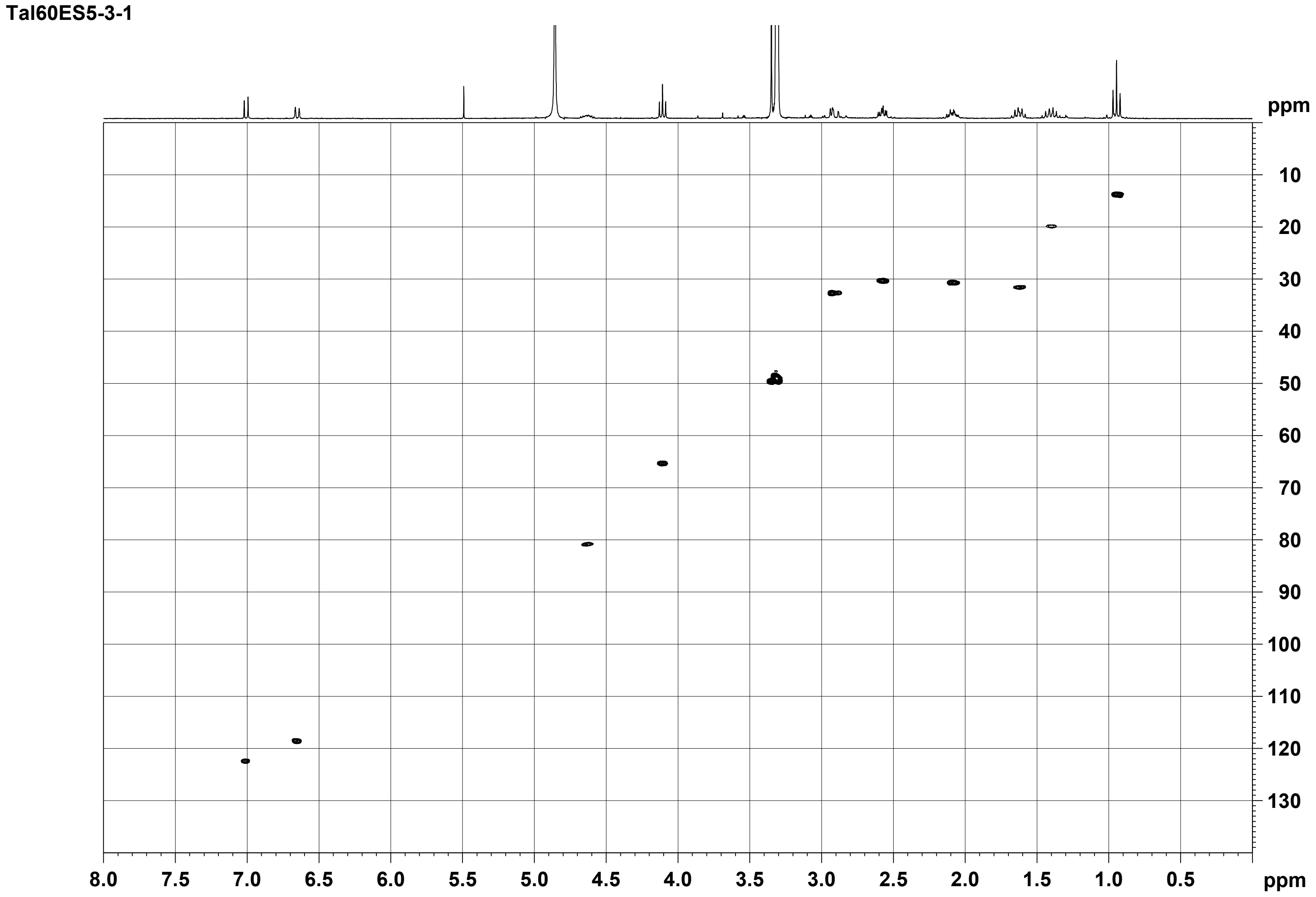

S90. HSQC (300 and 75 MHz, CD_3OD) spectrum of compound **16**.

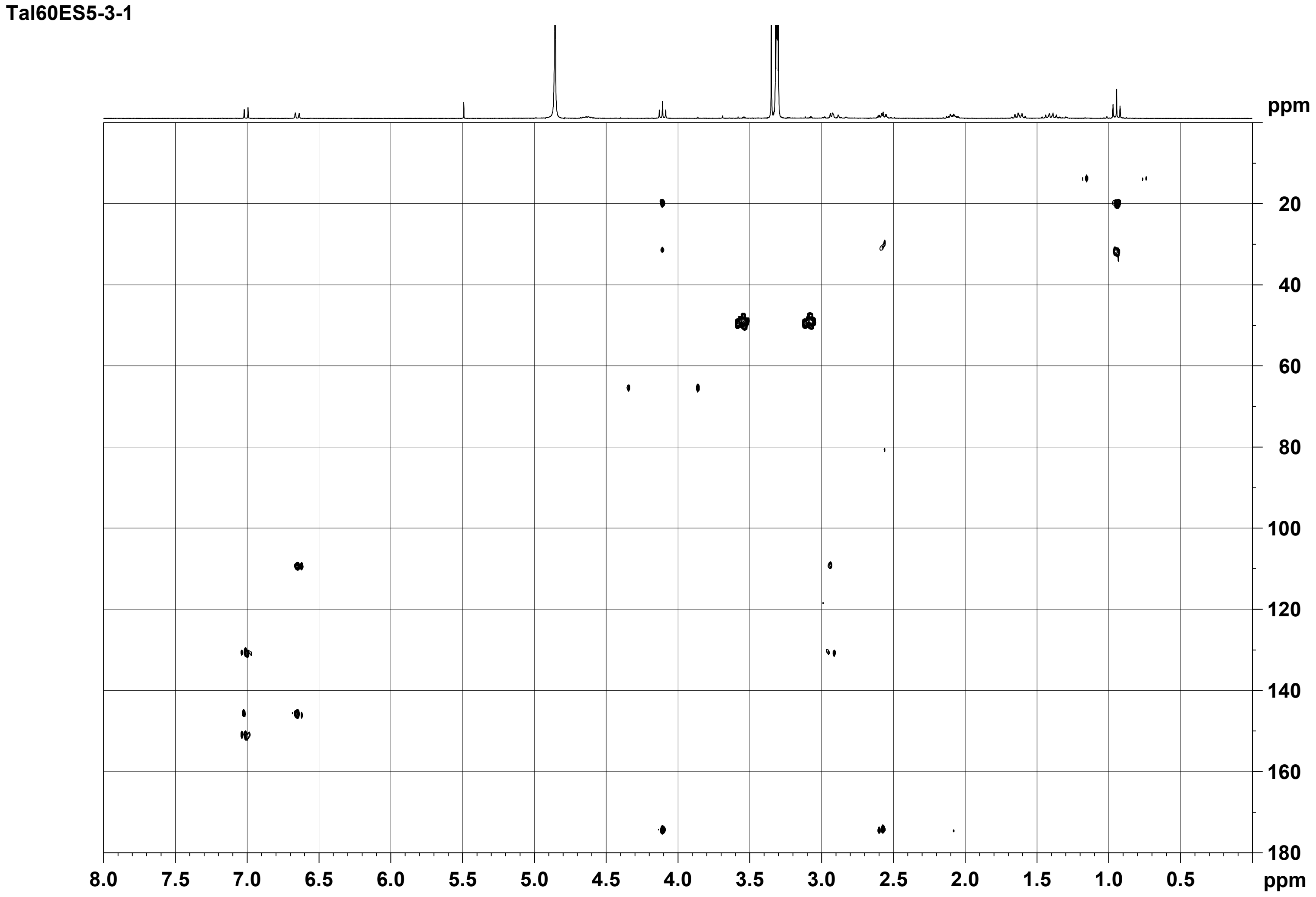

S91. HMBC (300 and 75 MHz, CD_3OD) spectrum of compound **16**.

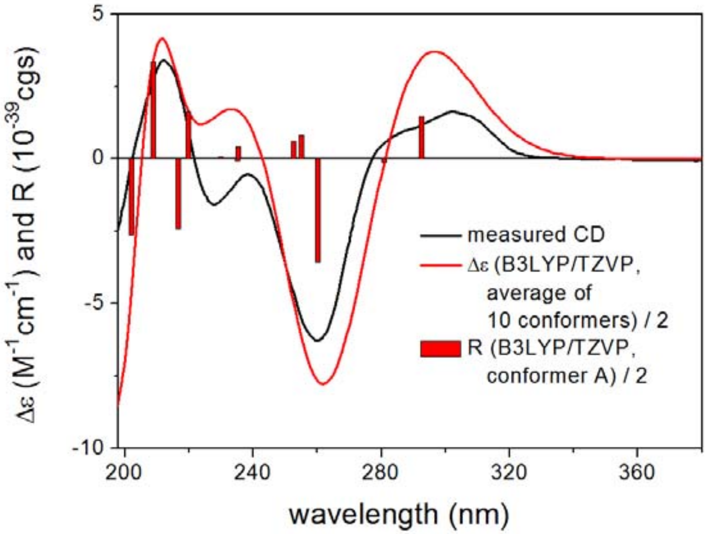

S92. Experimental ECD spectrum of **9** in MeCN compared with the Boltzmann-weighted B3LYP/TZVP PCM/MeCN ECD spectrum of (3*S*,7*S*)-**9** computed for the B97D/TZVP PCM/MeCN conformers. Bars represent the rotational strength values of the lowest-energy conformer.

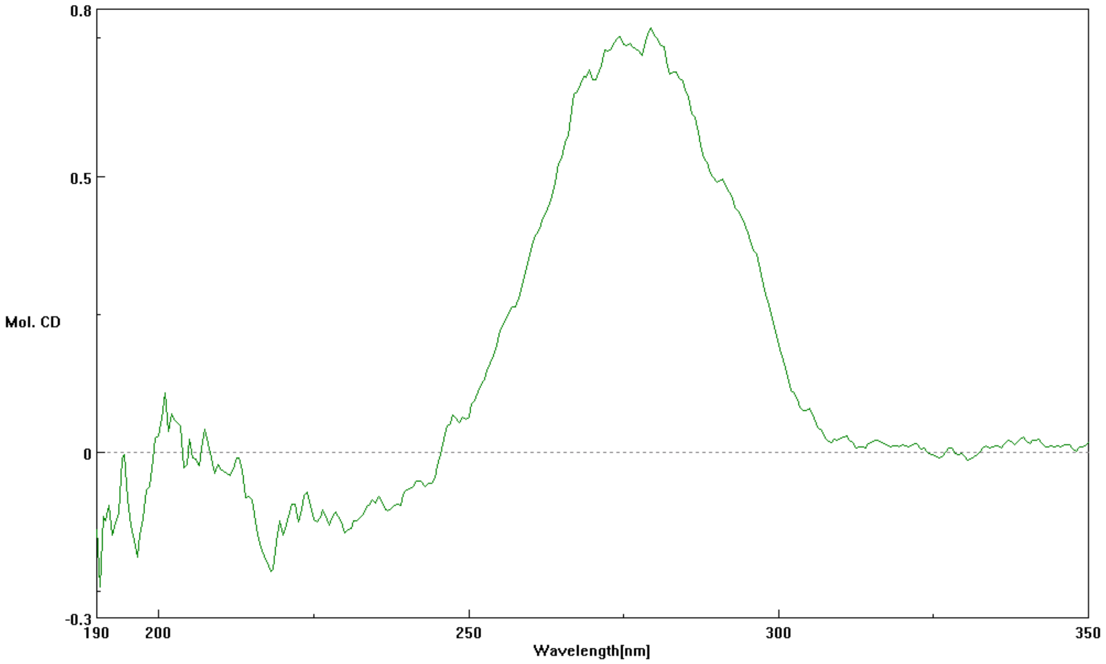

S93. Experimental ECD spectrum of **1** in MeCN

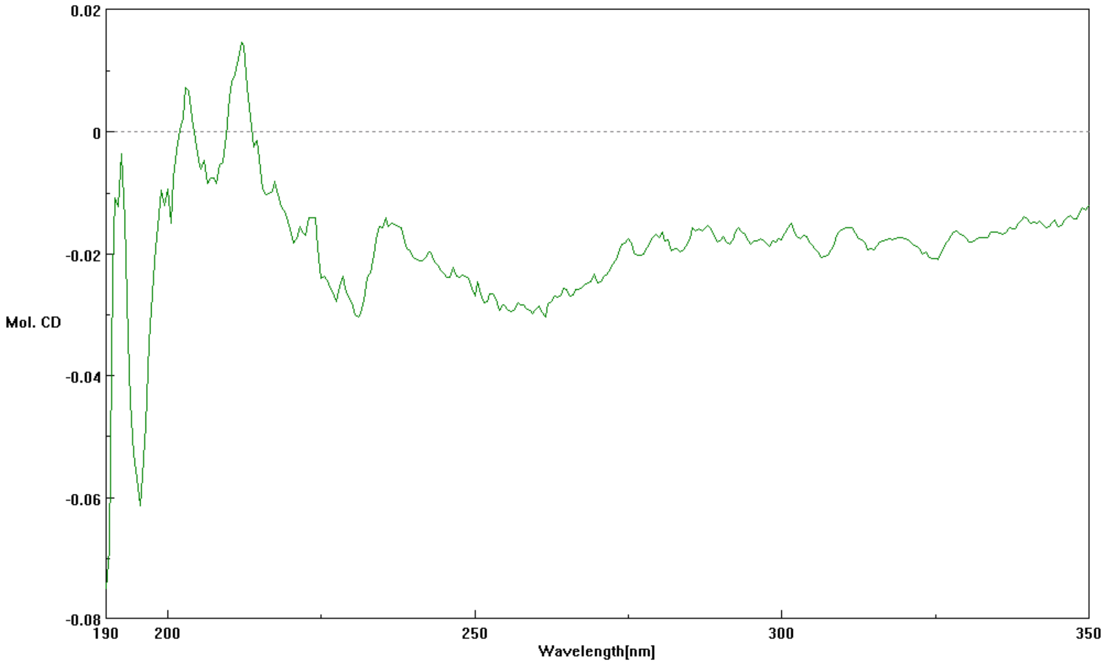

S94. Experimental ECD spectrum of **2** in MeCN

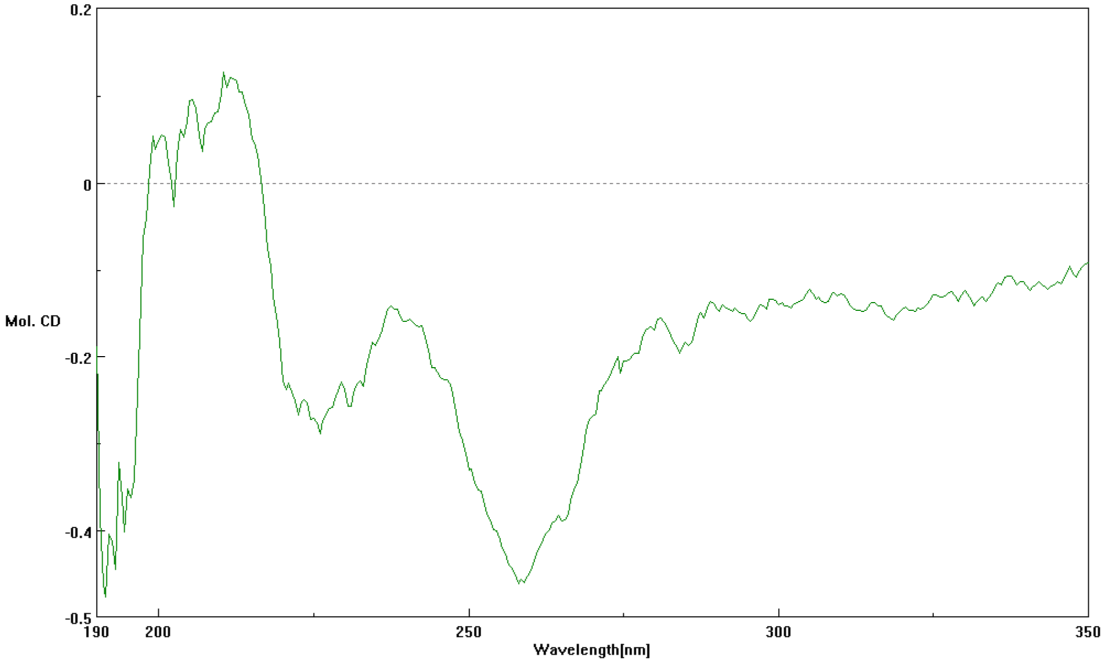

S95. Experimental ECD spectrum of **5** in MeCN

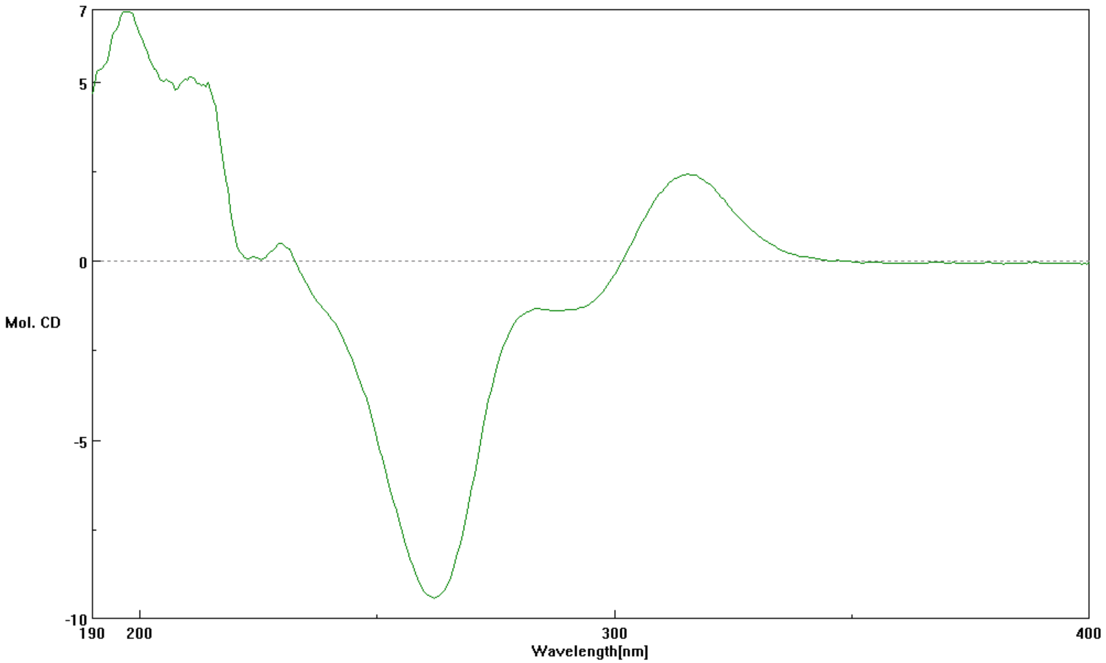

S96. Experimental ECD spectrum of **6** in MeCN

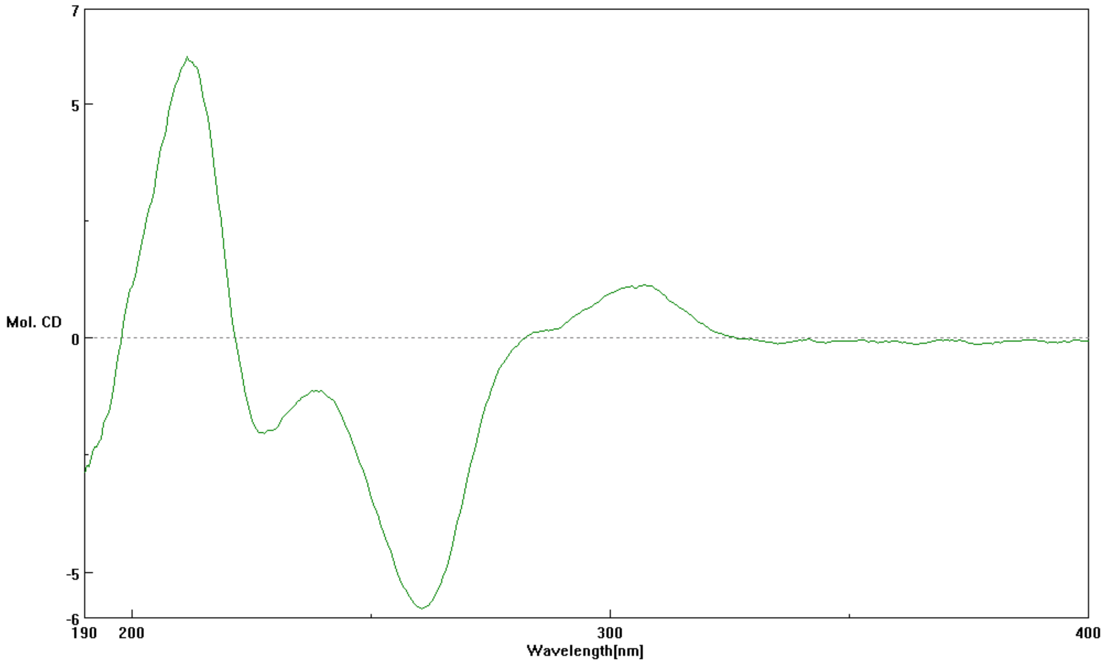

S97. Experimental ECD spectrum of **7** in MeCN

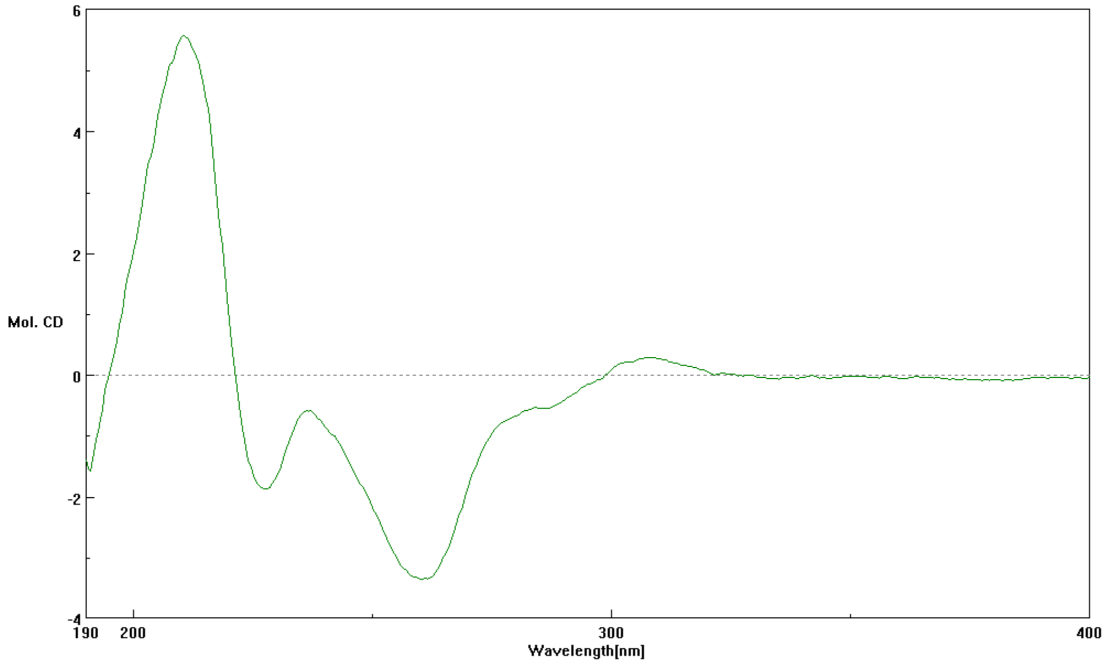

S98. Experimental ECD spectrum of **8** in MeCN

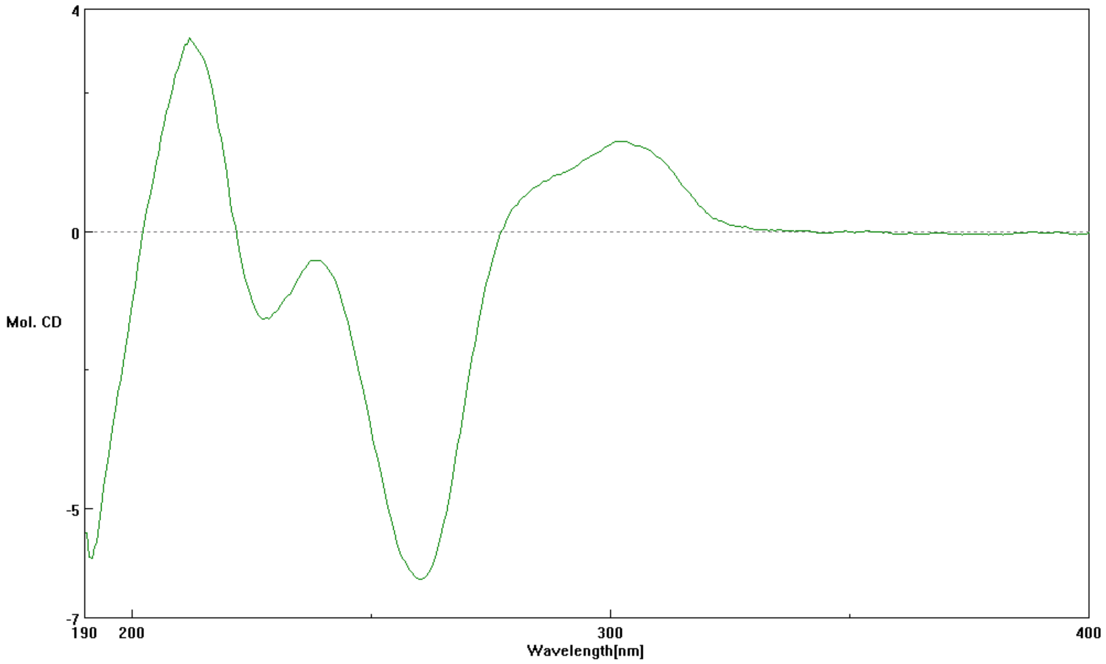

S99. Experimental ECD spectrum of **9** in MeCN

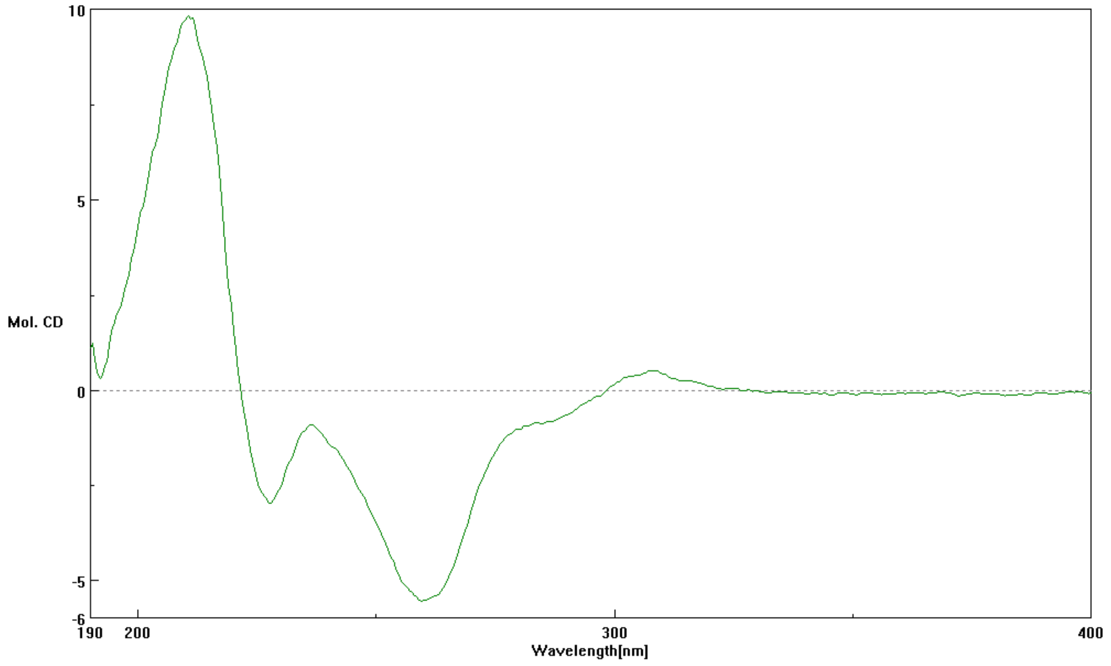

S100. Experimental ECD spectrum of **10** in MeCN

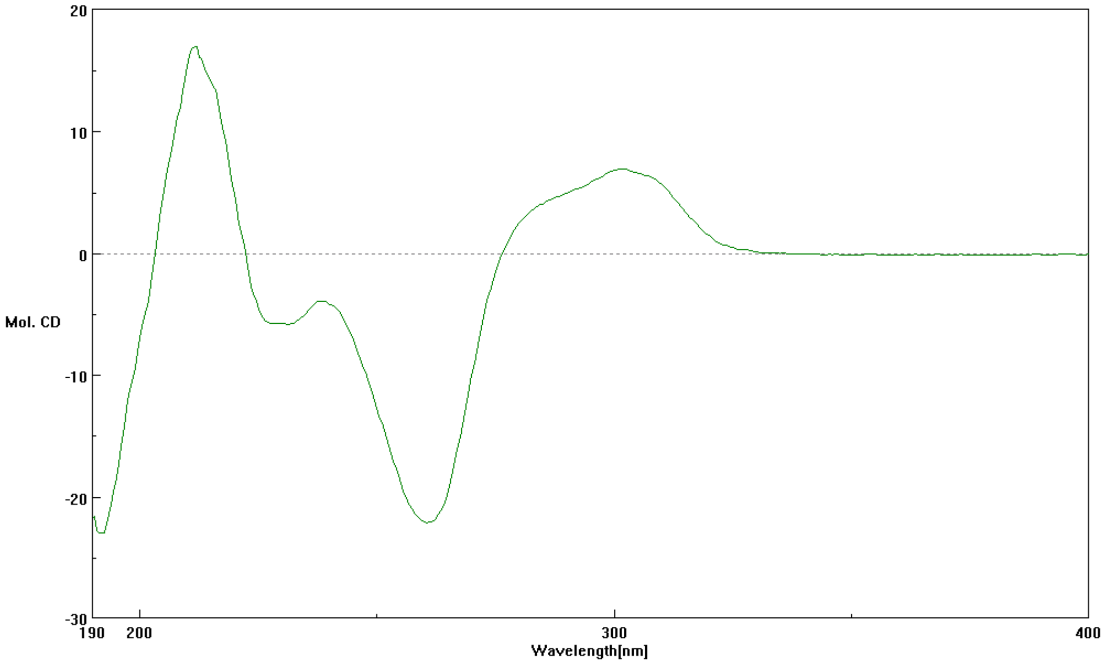

S101. Experimental ECD spectrum of **11** in MeCN

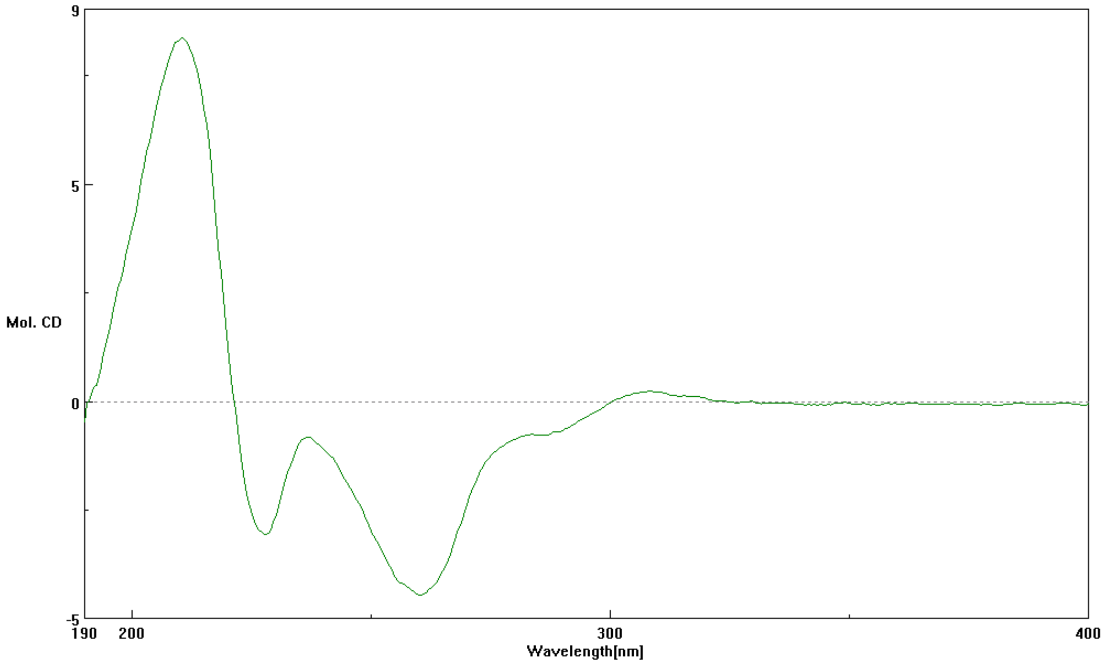

S102. Experimental ECD spectrum of **12** in MeCN

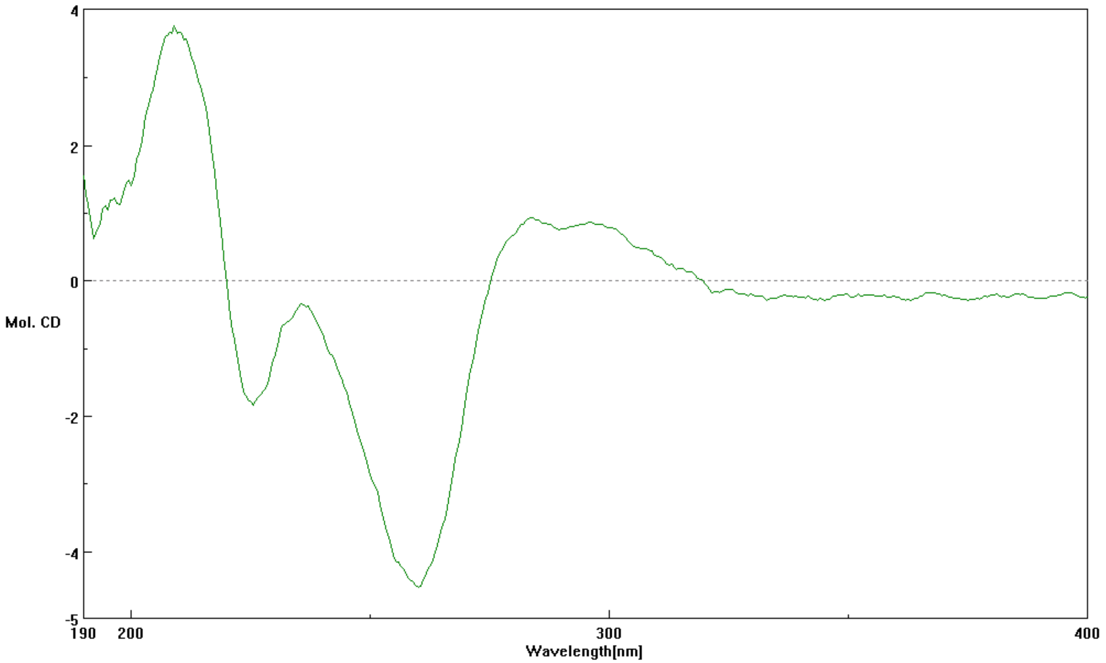

S103. Experimental ECD spectrum of **13** in MeCN

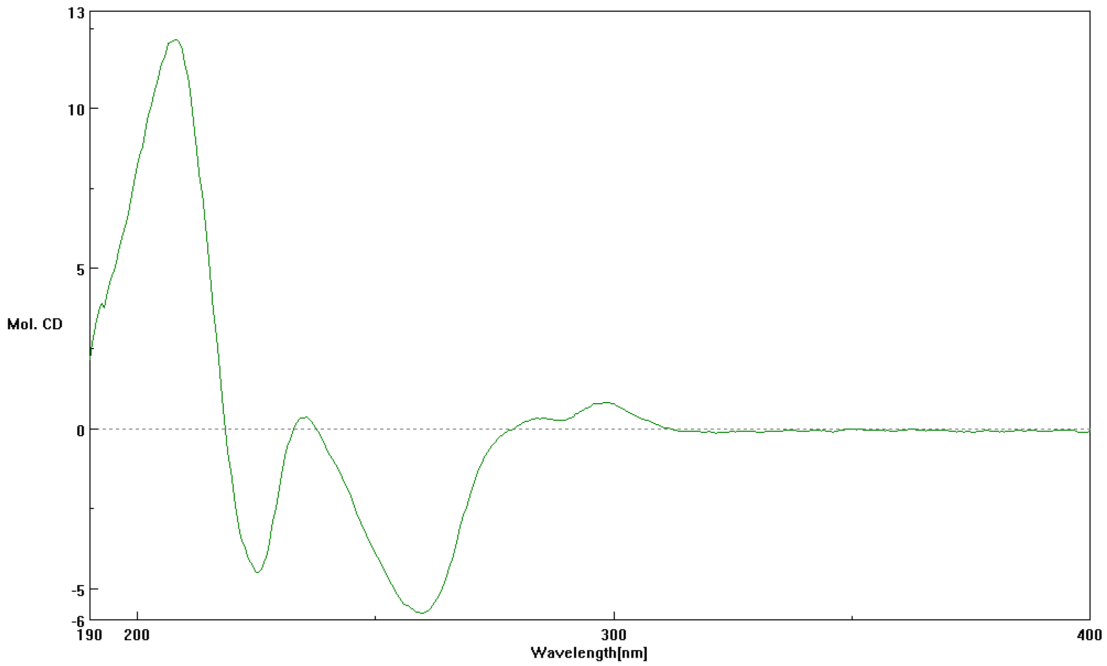

S104. Experimental ECD spectrum of **14** in MeCN

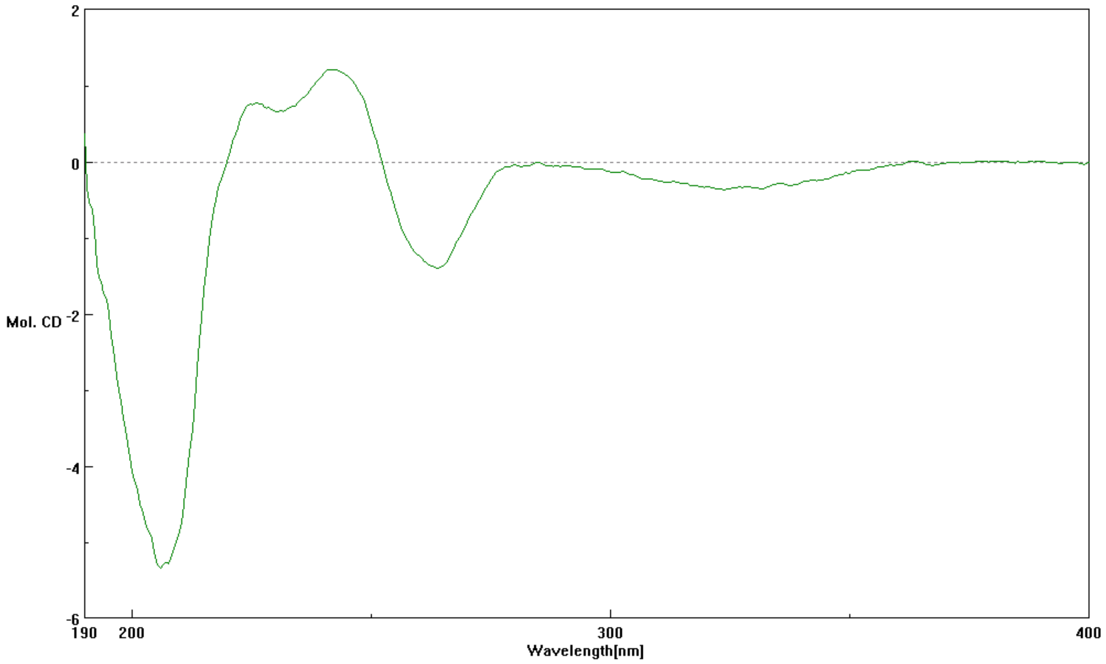

S105. Experimental ECD spectrum of **15** in MeCN

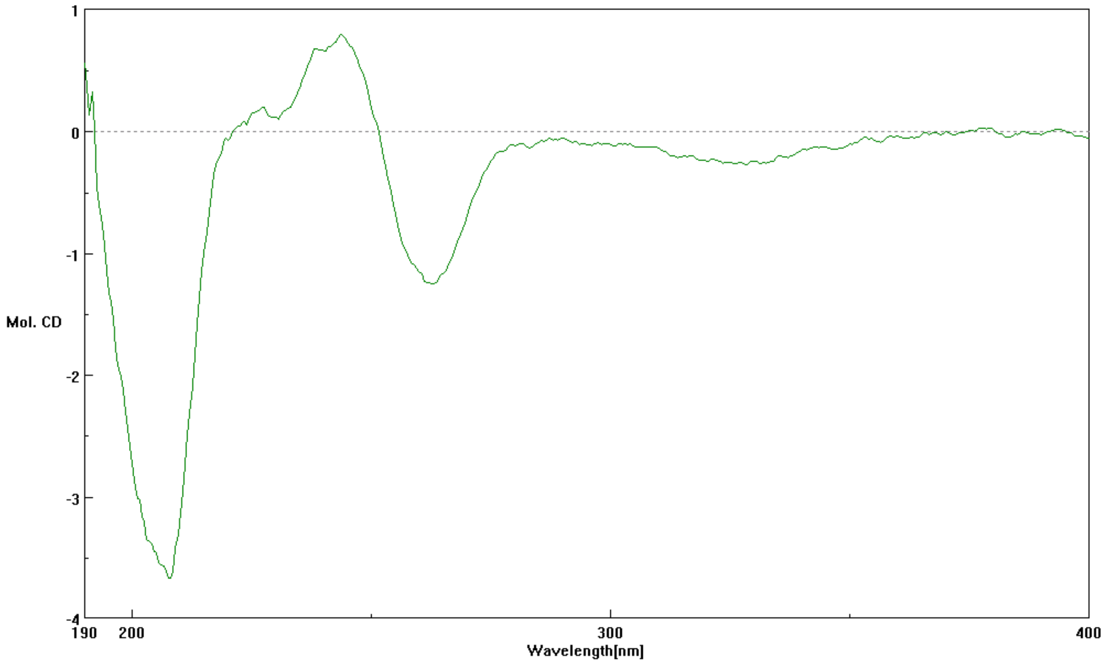

S106. Experimental ECD spectrum of **16** in MeCN
